# Supplementary material for: Changes in blood pressure thresholds for initiating antihypertensive medication in patients with diabetes: a repeated cross-sectional study focusing on the impact of age and frailty
Source: BMJ Open. 2020 Sep 10;10(9):e037694. doi: 10.1136/bmjopen-2020-037694 (PMC7485238; doi:10.1136/bmjopen-2020-037694)
Supplement: Supplementary data [file bmjopen-2020-037694supp001.pdf]

Ambrož M et al. Changes in blood pressure thresholds for initiating antihypertensive medication in diabetes patients with diabetes. BMJ Open 2020

Supplementary material 1

Exclusion of patients in each calendar year

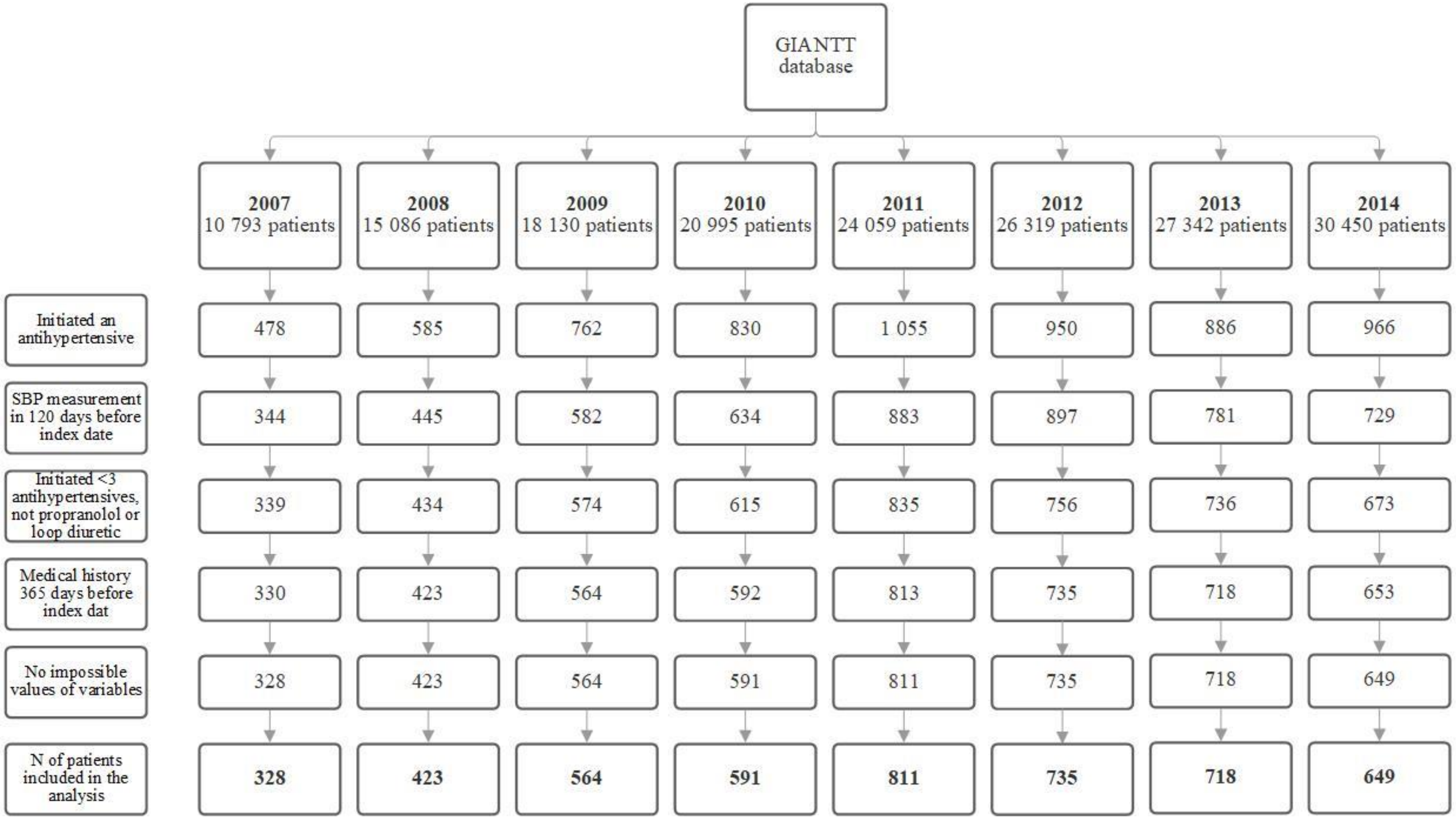

Supplementary Figure 1: Numbers of individuals available at each stage based on the inclusion and exclusion criteria per calendar year

Ambrož M et al. Changes in blood pressure thresholds for initiating antihypertensive medication in diabetes patients with diabetes. BMJ Open 2020

Supplementary Table 1: Characteristics of included patients over the years

	2007	2008	2009	2010	2011	2012	2013	2014
**Number of patients**	328	423	564	591	811	735	718	649
**Females; N (%)**	154 (47)	178 (42)	270 (48)	277 (47)	360 (44)	355 (48)	355 (49)	310 (48)
**Age in years; N (%)**								
< 60	98 (30)	161 (38)	203 (36)	226 (38)	275 (34)	238 (32)	212 (30)	207 (32)
60 – 69	111 (34)	139 (33)	199 (35)	189 (32)	282 (35)	237 (32)	241 (34)	187 (29)
70 - 79	84 (26)	84 (20)	106 (19)	121 (20)	191 (24)	164 (22)	163 (23)	155 (24)
≥ 80	35 (11)	39 (9)	56 (10)	55 (9)	63 (8)	96 (13)	102 (14)	100 (15)
**Frailty in electronic Frailty Index score; N (%)**								
Less frail: 0 – 0.03	193 (59)	233 (55)	303 (54)	275 (47)	372 (46)	306 (42)	231 (32)	157 (24)
Medium frail: 0.06 – 0.09	92 (28)	124 (29)	180 (32)	204 (35)	270 (33)	267 (36)	273 (38)	218 (34)
More frail: 0.11 – 0.40	43 (13)	66 (16)	81 (14)	112 (19)	169 (21)	162 (22)	214 (30)	274 (42)
**Systolic BP at initiation in mmHg; mean ± SD**	157 ± 22	157 ± 21	158 ± 21	157 ± 22	155 ± 21	154 ± 21	152 ± 22	151 ± 22
**Diastolic BP at initiation in mmHg; mean ± SD**	86 ± 12	87 ± 11	87 ± 12	87 ± 11	86 ± 12	85 ± 12	84 ± 12	84 ± 13
**Diabetes duration < 2 years; N (%)**	96 (29)	137 (32)	139 (25)	168 (28)	236 (29)	198 (27)	154 (21)	131 (20)
**HbA1c < 7%; N (%)**	150 (59)	232 (61)	308 (59)	323 (60)	497 (64)	454 (65)	418 (60)	335 (55)
**BMI in kg/m^2; N (%)**								
< 24.9	49 (19)	67 (19)	66 (14)	79 (15)	114 (15)	132 (19)	126 (18)	118 (19)
25 – 29.9	100 (39)	142 (39)	218 (46)	222 (42)	312 (40)	280 (40)	283 (41)	231 (38)
> 30	108 (42)	151 (42)	191 (40)	229 (43)	345 (45)	284 (41)	283 (41)	262 (43)
**Dyslipidaemia; N (%)**	92 (46)	161 (50)	232 (55)	269 (58)	418 (60)	403 (62)	366 (57)	322 (58)
**eGFR ≤ 60 ml/min/1.73m2; N (%)**	37 (17)	45 (14)	69 (15)	44 (9)	70 (10)	93 (14)	100 (15)	84 (14)
**Albuminuria; N (%)**	5 (4)	9 (4)	12 (4)	10 (3)	15 (4)	16 (4)	18 (5)	16 (4)
**History of cardiovascular disease; N (%)**								
Myocardial disease	11 (3)	21 (5)	16 (3)	21 (4)	31 (4)	50 (7)	55 (8)	58 (9)
Heart failure	5 (2)	4 (1)	6 (1)	6 (1)	20 (2)	12 (2)	15 (2)	22 (3)
Stroke	13 (4)	11 (3)	19 (3)	20 (3)	25 (3)	34 (5)	45 (6)	45 (7)
**N of chronic medication at initiation; mean ± SD**	3.8 ± 2.3	3.3 ± 2.1	3.4 ± 2.4	3.5 ± 2.2	3.4 ± 2.3	3.7 ± 2.6	3.6 ± 2.5	4.0 ± 2.8

Ambrož M et al. Changes in blood pressure thresholds for initiating antihypertensive medication in diabetes patients with diabetes. BMJ Open 2020

**Glucose lowering medication at initiation; N (%)**								
No medication	77 (23)	90 (21)	149 (26)	137 (23)	200 (25)	228 (31)	217 (30)	171 (26)
1 oral	129 (39)	179 (42)	199 (35)	247 (42)	363 (45)	271 (37)	283 (39)	266 (41)
2 oral	71 (22)	103 (24)	144 (26)	139 (24)	161 (20)	143 (19)	121 (17)	100 (15)
3 oral or more and/or insulin	51 (16)	51 (12)	72 (13)	68 (12)	87 (11)	93 (13)	97 (14)	112 (17)
**Treated with a lipid lowering drug; N (%)**	219 (67)	255 (60)	298 (53)	343 (58)	447 (55)	417 (57)	408 (57)	362 (56)
**Initiated drug class; N (%)**								
Renin-angiotensin-aldosterone system inhibitor	185 (60)	241 (61)	330 (63)	319 (60)	453 (62)	415 (63)	379 (60)	323 (56)
Diuretic	59 (19)	73 (18)	101 (19)	104 (20)	123 (17)	108 (16)	89 (14)	105 (18)
Beta blocker	51 (17)	59 (15)	75 (14)	80 (15)	117 (16)	93 (14)	116 (18)	107 (19)
Calcium channel blocker	14 (5)	22 (6)	21 (4)	29 (5)	32 (4)	40 (6)	44 (7)	38 (7)
Combination of antihypertensives	19 (6)	28 (7)	37 (7)	59 (10)	86 (11)	79 (11)	90 (13)	76 (12)

Ambrož M et al. Changes in blood pressure thresholds for initiating antihypertensive medication in diabetes patients with diabetes. BMJ Open 2020

Supplementary material 2

Sensitivity analysis using mean of last two blood pressure measurements (N =2 947)

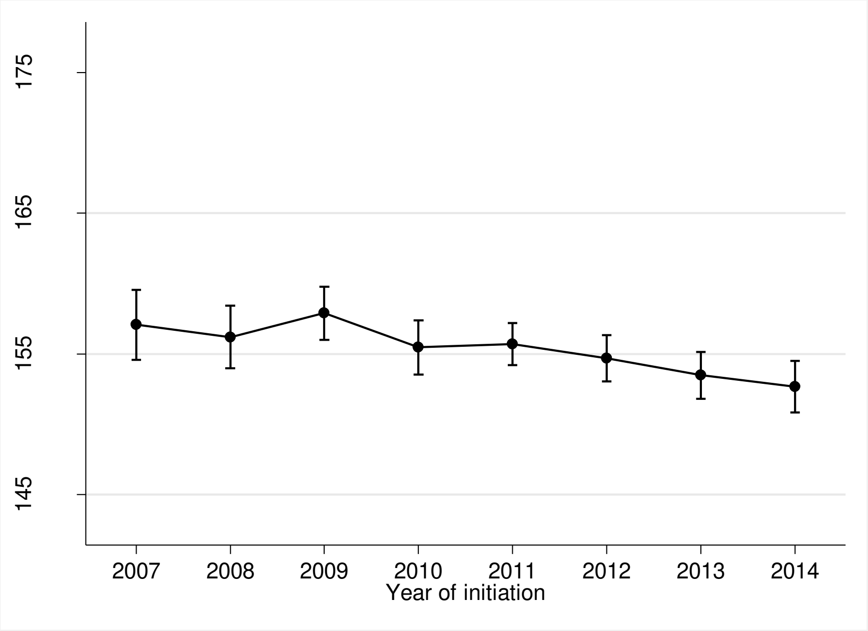

(A)

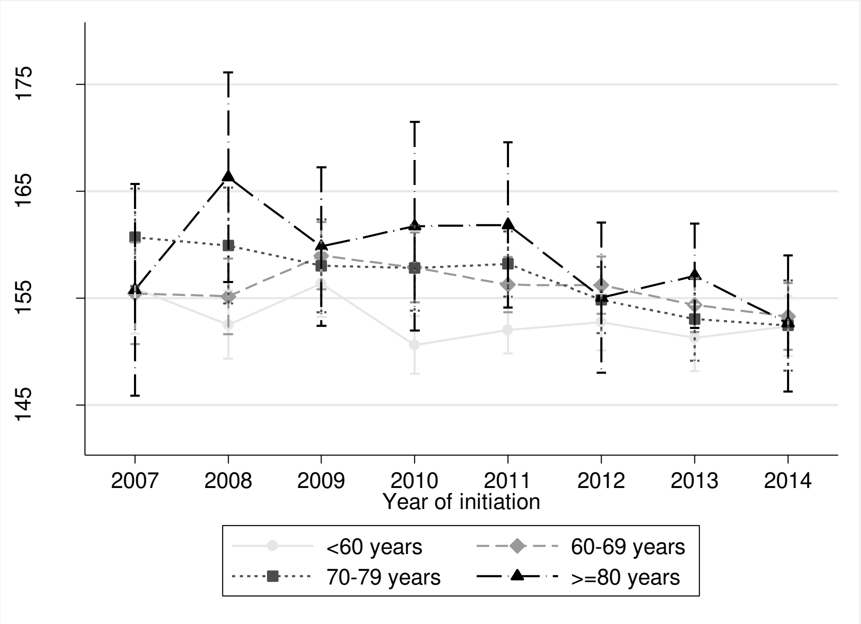

(B)

Ambrož M et al. Changes in blood pressure thresholds for initiating antihypertensive medication in diabetes patients with diabetes. BMJ Open 2020

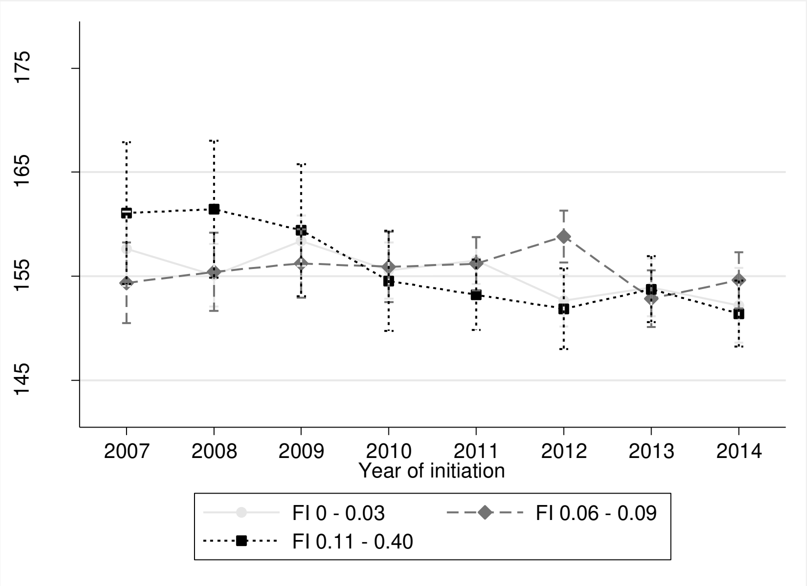

(C)

Supplementary Figure 2: Mean of last two systolic blood pressure (BP) levels with 95% CIs before/at antihypertensive treatment initiation (A) through the years; (B) through the years in different age groups and (C) through the years in different frailty groups.

Supplementary Table 2: Influence of calendar year and age or frailty on blood pressure thresholds (multilevel analysis) using the mean of last two systolic blood pressure measurements

	β	95% CI	P	
**AGE**[*]				
Calendar year	-0.894	-2.307, 0.519	0.215	<0.001[‡]
Calendar year^2	-0.001	-0.146, 0.148	0.993	
Age <60 years	-8.367	-10.910, -5.824	<0.001	
Age 60 – 69 years	-4.271	-6.714, -1.828	0.001	
Age 70 – 79 years	-2.921	-5.349, -0.492	0.018	
Age ≥80 years		reference group		
Interaction year*age		none are significant		
**FRAILTY**[†]				
Calendar year	-0.867	-2.290, 0.556	0.232	<0.001[‡]
Calendar year^2	-0.013	-0.161, 0.136	0.866	
Frailty 0 – 0.03	-3.501	-5.390, -1.611	0.000	
Frailty 0.06 – 0.09	-1.455	-3.231, 0.322	0.109	
Frailty 0.11 – 0.40		reference group		
Interaction year*frailty		none are significant		

The intraclass correlation coefficient (ICC) calculated from the empty model was 0.050.

[*] the age model was adjusted for sex, duration of diabetes, number of chronic medication at initiation, number and/or type of glucose lowering therapy, lipid lowering therapy, presence of albuminuria, presence of dyslipidaemia, haemoglobin A1C, history of cardiovascular events, estimated glomerular filtration rate and BMI

[†] the frailty model was adjusted for sex, duration of diabetes, number and/or type of glucose lowering therapy, lipid lowering therapy and haemoglobin A1C

[‡] joint significance of calendar year and calendar year^2 using Wald test

Ambrož M et al. Changes in blood pressure thresholds for initiating antihypertensive medication in diabetes patients with diabetes. BMJ Open 2020

Supplementary material 3

Sensitivity analysis using electronic frailty index as a continuous variable

There were no statistically significant differences in blood pressure thresholds between patients with different frailty (Supplementary table 3).

Supplementary Table 3: Influence of calendar year and frailty on blood pressure thresholds (multilevel analysis), using frailty index as a continuous variable

	**β**	**95% CI**	**P**	
**FRAILTY**[†]				
Calendar year	0.211	-1.135, 1.557	0.759	<0.001[‡]
Calendar year^2	-0.147	-0.288, -0.007	0.039	
Frailty	-9.324	-20.747, 2.098	0.110	
Interaction year*frailty		none are significant		

[†] the frailty model was adjusted for sex, duration of diabetes, number and/or type of glucose lowering therapy, lipid lowering therapy and haemoglobin A1C
[‡] joint significance of calendar year and calendar year^2 using Wald test

Ambrož M et al. Changes in blood pressure thresholds for initiating antihypertensive medication in diabetes patients with diabetes. BMJ Open 2020

Supplementary material 4

Supplementary Table 4: Characteristics of included patients: comparison of complete cases and cases with missing values

	complete cases	cases with missings
Patients; N (%)	3 545 (74)	1 274 (26)
Females; N (%)	1 602 (45)	675 (52)
Age in years; mean ± SD	64 ± 12	65 ± 13
Frailty index; median (Q1 - Q3)	0.08 (0.06 – 0.11)	0.08 (0.03 – 0.11)
SBP at initiation in mmHg; mean ± SD	154 ± 21	158 ± 23
DBP at initiation in mmHg; mean ± SD	85 ± 12	87 ± 13
Diabetes duration in years; mean ± SD	5.4 ± 5.3	5.4 ± 5.5
N of chronic medication at initiation; mean ± SD	3.6 ± 2.4	3.5 ± 2.6
Treated with a lipid lowering drug; N (%)	2 134 (60)	615 (48)
Initiated with one antihypertensive; N (%)	3 215 (91)	1 130 (89)

Ambrož M et al. Changes in blood pressure thresholds for initiating antihypertensive medication in diabetes patients with diabetes. BMJ Open 2020

Supplementary material 5

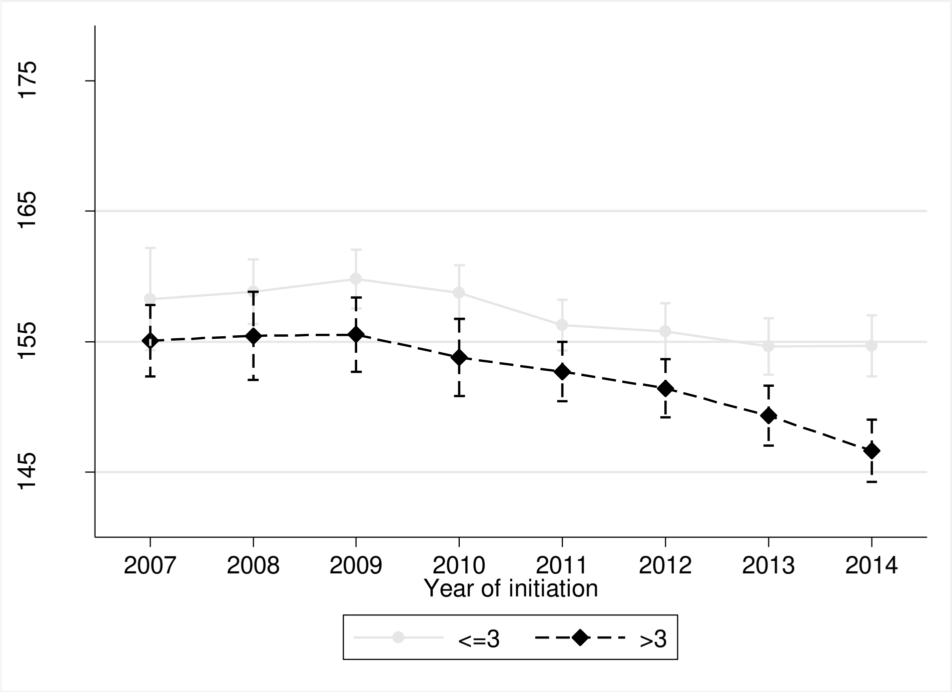

Supplementary Figure 3: Mean last systolic blood pressure value with 95% CIs before/at antihypertensive treatment initiation through the years in patients with different number of chronic medication at initiation.

Supplementary table 5: Multilevel analysis of number of chronic medication at initiation

	**β**	**95% CI (min, max)**	**P**	
**Calendar year**	-0.206	-1.543, 1.130	0.762	<0.001[‡]
**Calendar year^2**	-0.101	-0.240, 0.037	0.152	
**3 or less chronic medication**		reference group		
**More than 3 chronic medication**	-4.107	-5.428, -2.785	<0.001	
**Interaction year*N of medication**		none are significant		

The model was adjusted for sex, duration of diabetes, number and/or type of glucose lowering therapy, lipid lowering therapy, presence of albuminuria, presence of dyslipidaemia, haemoglobin A1C, estimated glomerular filtration rate and BMI.

[‡] joint significance of calendar year and calendar year^2 using Wald test
