# Supplementary material for: Changes in blood pressure thresholds for initiating antihypertensive medication in patients with diabetes: a repeated cross-sectional study focusing on the impact of age and frailty
Source: BMJ Open. 2020 Sep 10;10(9):e037694. doi: 10.1136/bmjopen-2020-037694 (PMC7485238; doi:10.1136/bmjopen-2020-037694)
Supplement: Author's manuscript [file bmjopen-2020-037694.draft_revisions.pdf]

BMJ Open is committed to open peer review. As part of this commitment we make the peer review history of every article we publish publicly available.

When an article is published we post the peer reviewers' comments and the authors' responses online. We also post the versions of the paper that were used during peer review. These are the versions that the peer review comments apply to.

The versions of the paper that follow are the versions that were submitted during the peer review process. They are not the versions of record or the final published versions. They should not be cited or distributed as the published version of this manuscript.

BMJ Open is an open access journal and the full, final, typeset and author-corrected version of record of the manuscript is available on our site with no access controls, subscription charges or pay-per-view fees (http://bmjopen.bmj.com).

If you have any questions on BMJ Open's open peer review process please email info.bmjopen@bmj.com

BMJ Open

Changes in blood pressure thresholds for initiating antihypertensive medication in diabetes patients: A repeated cross-sectional study

Journal:	*BMJ Open*
Manuscript ID	bmjopen-2020-037694
Article Type:	Original research
Date Submitted by the Author:	13-Feb-2020
Complete List of Authors:	Ambrož, Martina; University Medical Centre Groningen,<br>de Vries, Sieta; University Medical Centre Groningen, Clinical Pharmacy and Pharmacology<br>Sidorenkov, Grigory; University Medical Centre Groningen, Epidemiology<br>Hoogenberg, Klaas; Martini Ziekenhuis<br>Denig, Petra; University Medical Centre Groningen, Clinical Pharmacy & Pharmacology
Keywords:	General diabetes < DIABETES & ENDOCRINOLOGY, PRIMARY CARE, Hypertension < CARDIOLOGY, EPIDEMIOLOGY, GERIATRIC MEDICINE, Protocols & guidelines < HEALTH SERVICES ADMINISTRATION & MANAGEMENT

SCHOLARONE™
Manuscripts

I, the Submitting Author has the right to grant and does grant on behalf of all authors of the Work (as defined in the below author licence), an exclusive licence and/or a non-exclusive licence for contributions from authors who are: i) UK Crown employees; ii) where BMJ has agreed a CC-BY licence shall apply, and/or iii) in accordance with the terms applicable for US Federal Government officers or employees acting as part of their official duties; on a worldwide, perpetual, irrevocable, royalty-free basis to BMJ Publishing Group Ltd ("BMJ") its licensees and where the relevant Journal is co-owned by BMJ to the co-owners of the Journal, to publish the Work in this journal and any other BMJ products and to exploit all rights, as set out in our licence.

The Submitting Author accepts and understands that any supply made under these terms is made by BMJ to the Submitting Author unless you are acting as an employee on behalf of your employer or a postgraduate student of an affiliated institution which is paying any applicable article publishing charge ("APC") for Open Access articles. Where the Submitting Author wishes to make the Work available on an Open Access basis (and intends to pay the relevant APC), the terms of reuse of such Open Access shall be governed by a Creative Commons licence – details of these licences and which Creative Commons licence will apply to this Work are set out in our licence referred to above.

Other than as permitted in any relevant BMJ Author's Self Archiving Policies, I confirm this Work has not been accepted for publication elsewhere, is not being considered for publication elsewhere and does not duplicate material already published. I confirm all authors consent to publication of this Work and authorise the granting of this licence.

For peer review only

Changes in blood pressure thresholds for initiating antihypertensive medication in diabetes patients: A repeated cross-sectional study

Martina Ambrož, MPharm[a]; Sieta T. de Vries, PhD[a]; Grigory Sidorenkov, PhD[b]; Klaas Hoogenberg, MD, PhD[c]; Petra Denig, PhD[a]

[a] Department of Clinical Pharmacy and Pharmacology, University of Groningen, University Medical Center Groningen, Hanzeplein 1, 9713 GZ, Groningen, The Netherlands

[b] Department of Epidemiology, University of Groningen, University Medical Center Groningen, Hanzeplein 1, 9713 GZ, Groningen, The Netherlands

[c] Department of Internal Medicine, Martini Hospital, Van Swietenplein 1, 9728 NT, Groningen, the Netherlands

Declarations of interest: none

Corresponding author: Martina Ambrož, PO Box 30.001, 9700 RB Groningen, +31 613914263, m.ambroz@umcg.nl

ORCID:

Martina Ambrož https://orcid.org/0000-0003-1319-4898

Sieta T. de Vries https://orcid.org/0000-0001-6090-2434

Grigory Sidorenkov https://orcid.org/0000-0002-1926-8862

Petra Denig https://orcid.org/0000-0002-7929-4739

Word count: 2 489

For peer review only - http://bmjopen.bmj.com/site/about/guidelines.xhtml

ABSTRACT

Objective

To assess trends in systolic blood pressure (SBP) thresholds at initiation of antihypertensive treatment in type 2 diabetes (T2D) patients and the impact of age and frailty on these trends.

Study Design and Setting

A repeated cross-sectional cohort study (2007-2013) using the Groningen Initiative to Analyze Type 2 diabetes Treatment (GIANTT) database was conducted. The influence of calendar year, age or frailty and the interaction between year and age or frailty on SBP thresholds were assessed using multilevel regression analyses adjusted for potential confounders.

Results

We included 4,170 patients. The mean SBP at treatment initiation was 155mmHg in 2007, rising to 158mmHg in 2009 and decreasing to 152mmHg in 2013. This quadratic trend was significant ($p<0.002$). Older patients initiated treatment at higher SBP, but similar decreasing trends after 2009 were observed in all age groups. There were no significant differences in SBP thresholds between patients with different frailty. The association between year and SBP threshold was not influenced by age or frailty.

Conclusion

After an initial rise, the observed SBP thresholds decreased over time and were not influenced by age or frailty. This is in contrast with guideline recommendations and indicates that changing prescribing practice may take considerable time.

Key words: diabetes mellitus, type 2; blood pressure; primary health care; drug utilization; elderly; frail

For peer review only - http://bmjopen.bmj.com/site/about/guidelines.xhtml

INTRODUCTION

Treatment of hypertension in patients with type 2 diabetes (T2D) reduces their cardiovascular risk, but guideline recommendations on when to initiate antihypertensive treatment to best balance the benefits and risks of treatment have changed over time. In general, the recommended systolic blood pressure (SBP) threshold for treatment initiation lies between 130 and 140mmHg (1-7). A personalized approach, however, has been advocated in the last decade for older and/or frail patients, who are at increased risk of adverse outcomes related to low BP levels (8). Since 2011, treatment guidelines recommend higher SBP thresholds in these patients (Figure 1).

A recent interview study showed that Dutch general practitioners were indeed somewhat reluctant to initiate antihypertensive treatment in older and/or frail patients (13). Studies showing at which SBP thresholds physicians initiate antihypertensive treatment in older or frail patients are lacking.

A Danish study observed an average SBP level in the general population of 148mmHg before they received antihypertensive treatment in the period from 1976 to 2004 (14). Trend studies on antihypertensive medication use and hypertension control in individuals with T2D show that the percentage of people achieving the recommended SBP target of <140mmHg increased over the last twenty years (15,16). An observational study conducted in the Netherlands showed that the mean achieved SBP decreased from 155mmHg in 1998 to 140mmHg in 2008 in all age groups, with a mean SBP being lower in younger patients. No relevant differences in trends were observed between different age groups (17). This indicates that risk factor control in patients with T2D has generally improved over time. The extent to which the more recent personalized guideline recommendations are followed is not clear. This may depend on physician or practice characteristics (18,19), resulting in variability between treatment decisions (20).

For peer review only - http://bmjopen.bmj.com/site/about/guidelines.xhtml

The aim of this study was to assess trends in SBP thresholds for initiating antihypertensive medication in patients with T2D and the impact of changed treatment recommendations for older and frail patients. We looked at the period between 2007 and 2013, for which we hypothesized that SBP thresholds would remain similar among young and non-frail patients but would increase among older and/or frail patients. Our secondary aim was to assess to what extent SBP thresholds for treatment initiation varied across general practices.

METHODS

Study design and population

This is a repeated cross-sectional dynamic cohort study for the years 2007 to 2013. The Groningen Initiative to Analyse Type-2 diabetes Treatment (GIANTT; www.giantt.nl) database was used, which contains anonymous electronic medical records data of patients with T2D treated in primary care in the north of the Netherlands.

Patients were included per calendar year when they had a diagnosis of T2D, were ≥18 years, were included in the database for at least 365 days before antihypertensive treatment initiation, and initiated treatment with an antihypertensive (Anatomical Therapeutic Chemical (ATC) codes C03, C04, C07, C08, C09) in that year. Antihypertensive treatment initiation was defined as an initial prescription without a prescription of any antihypertensive drug in the preceding 365 days. Patients were excluded when they did not have a documented SBP level within 120 days before or at the day of treatment initiation or when they initiated treatment with three or more drug classes, since it is unlikely that this was a true initiation. Moreover, we excluded patients initiating propranolol or a loop diuretic (furosemide, bumetanide), since these are commonly prescribed for other indications (i.e migraine prophylaxis or short term use in patients with edema, respectively). No approval from an ethics committee is needed for studies using data from anonymous medical records in the

For peer review only - http://bmjopen.bmj.com/site/about/guidelines.xhtml

Netherlands. An exemption letter from University Medical Center Groningen Medical Ethics Review Board was obtained (reference number M19.235285).

Outcome variable

The outcome was the patient's most recent office SBP level in the 120 days before or on the day of antihypertensive treatment initiation.

Explanatory variables

The following explanatory variables were included: calendar year, age or frailty of the patient and the interaction between year and age or frailty. Age was calculated on January 1 of each year and was categorized in four groups (<60 years, 60–69 years, 70–79 years and ≥80 years) related to cut-off values mentioned in several guidelines (7,12,21). Frailty was calculated using a previously developed electronic frailty index (eFI) based on International Classification of Primary Care (ICPC) coded diagnoses from the medical history (22). This eFI ranges from 0 to 1, with a higher number indicating a more frail patient. Since there are no validated clinical cut-off values for the eFI, we categorized the scores in tertiles to compare low-, medium- and high frailty patients.

Confounders

The following patient characteristics were included as possible confounders: sex, diabetes duration (<2 years or ≥2 years), presence of dyslipidemia (LDL ≥2.5 mmol/L), glycated hemoglobin level (HbA_{1c} <7% or ≥7%), estimated glomerular filtration rate (eGFR; ≤60 mL/min or >60 mL/min), presence of albuminuria (albumin creatinine ratio ≥30 mg/g or albumin in 24h urine ≥300 mg), history of cardiovascular events (presence yes/no of myocardial disease, heart failure or stroke), body mass index (BMI; <24.9 kg/m^2, 25–29.9 kg/m^2 or ≥30 kg/m^2), number of prescribed chronic medication (continuous variable), number and type of glucose lowering treatment (none, one oral, two oral, three or more oral and/or insulin), and lipid lowering treatment (none or one/more drug classes). The most recent

For peer review only - http://bmjopen.bmj.com/site/about/guidelines.xhtml

laboratory values available in the 365 days before or seven days after treatment initiation were used. BMI was calculated from weight and height or extracted from the database in case these were not available. The eGFR was calculated from serum creatinine using the Modification of Diet in Renal Disease-4 equation for the years 2007 to 2009, and using the Chronic Kidney Disease Epidemiology Collaboration equation from 2010 onwards, since the standard way of calculating eGFR in the Netherlands changed during the study period (23). In case serum creatinine was not available, the eGFR was extracted from the database when available. Prescribed medication was assessed in the 120 days before or at the day of treatment initiation.

Missing data

There were no missing data for the explanatory variables. Values of confounders with <20% of missing values were imputed using multiple imputation by chained equation (24). For patients without albuminuria measurements (47%), we assumed that they did not have albuminuria, since urine samples were less likely to be collected in our study period for patients without suspected renal function problems.

Analyses

Descriptive statistics were performed to examine patient characteristics per calendar year.

We conducted multilevel regression analyses with a two-level random intercept model to account for patients being nested within general practices. First, using the empty model, which includes only the outcome variable, we calculated the intraclass correlation coefficient to assess the variance that is attributed to general practices. Second, we added the confounders to assess the overall trend over the years. We compared a linear and a quadratic model using the Wald test to choose the best fitting final model. In the final model we assessed the effect

For peer review only - http://bmjopen.bmj.com/site/about/guidelines.xhtml

of age or frailty on the trends by adding the explanatory variables and the interaction between year and age or frailty on SBP levels at treatment initiation.

Additional subgroup analyses were conducted for each age and frailty group to assess changes over time in these subpopulations using the final model. After applying Bonferroni correction for multiple testing, significance levels were set at p<0.0125 (per age group) and p<0.0167 (per frailty group).

Furthermore, we conducted sensitivity analyses using the average of the last two SBP levels instead of a single SBP measurement and using eFI as a continuous variable in the final model.

The analyses were conducted in Stata version 14 (Stata Corp., College Station, TX).

Patient and public involvement

Patients and public were not involved in this study.

RESULTS

A total of 4,170 patients initiating antihypertensive treatment in the period 2007 to 2013 were included (Table 1). Patient characteristics were generally similar throughout the years (Supplementary Table1). Seventy-two percent of included patients had no missing values.

Table 1: Characteristics of included patients (N = 4,170)

**Number of patients in source population**		**Included patients**
	2007 (N = 10 793)	328
	2008 (N = 15 086)	423
	2009 (N = 18 130)	564
	2010 (N = 20 995)	591
	2011 (N = 24 059)	811
	2012 (N = 26 319)	735
	2013 (N = 27 342)	718
**Females; N (%)**		1,949 (47)
**Age in years; N (%)**		
	< 60	1 413 (34)
	60 – 69	1 398 (34)
	70 – 79	913 (22)
	≥ 80	446 (11)
**Frailty in electronic Frailty Index score; N (%)**		
	0 – 0.06	1 954 (47)

For peer review only - http://bmjopen.bmj.com/site/about/guidelines.xhtml

	0.08 – 0.11	1 383 (33)
	0.14 – 0.36	833 (20)
**Systolic BP at initiation in mmHg; mean ± SD**		155 ± 22
**Diastolic BP at initiation in mmHg; mean ± SD**		86 ± 12
**Diabetes duration <2 years; N (%)**		1 128 (27)
**HbA_{1c} < 7%; N (%)** †		2 382 (62)
**BMI in kg/m^2; N (%)** ‡		
	< 24.9	633 (17)
	25 – 29.9	1 557 (41)
	≥ 30	1 591 (42)
**Dyslipidaemia; N (%)** §		1 941 (57)
**eGFR ≤ 60 ml/min/1.73m2; N (%)** ¶		458 (13)
**Albuminuria (%)** ¦¦		85 (4)
**History of cardiovascular disease (N (%))**		
	Myocardial disease[1]	205 (5%)
	Heart failure[2]	68 (2%)
	Stroke[3]	167 (4%)
**Number of chronic medication at initiation; mean ± SD**		3.5 ± 2.4
**Glucose lowering medication at initiation (N (%))**		
	No medication	1 098 (26)
	1 oral	1 671 (40)
	2 oral	882 (21)
	3 oral or more and/or insulin	519 (12)
**Treated with a lipid lowering drug; N (%)**		2 387 (57)
**Initiated drug class; N (%)**		
	Renin-angiotensin-aldosterone system inhibitor	2 322 (56)
	Diuretic	657 (16)
	Beta blocker	591 (14)
	Calcium channel blocker	202 (5)
	Combination of antihypertensives	398 (10)

†hemoglobin A1C (HbA_{1c}): 314 (7.5 %) missing values; ‡BMI: 389 (9.3 %) missing values; § LDL-cholesterol: 783 (18.8 %) missing values; ¶ estimated glomerular filtration rate (eGFR): 619 (14.8 %) missing values; ¦¦ albuminuria: 1 958 (47.0%) missing values; [1] acute myocardial infarction (ICPC code k75) in the last year or other/chronic ischaemic heart disease (ICPC code k76) anytime in history; [2] heart failure (ICPC code k77) anytime in history; [3] transient cerebral ischemia (ICPC code K89) in the last year or stroke/cerebrovascular incident (ICPC code K90) anytime in history

Trends in SBP thresholds

The mean SBP level at antihypertensive treatment initiation significantly changed over time from 155mmHg (SD 22mmHg) in 2007, rising to 158mmHg (SD 21mmHg) in 2009 and

For peer review only - http://bmjopen.bmj.com/site/about/guidelines.xhtml

thereafter decreasing to 152mmHg (SD 22mmHg) in 2013 (Figure 2A). This quadratic trend was statistically significant (p=0.001).

Age and frailty

Older patients initiated treatment at significantly higher SBP thresholds than younger patients but age did not significantly influence the relationship between calendar year and SBP threshold (Table 2, Figure 2B). In the analyses per age group, the SBP level at initiation changed significantly (quadratic model) over the years in patients younger than 60 years (p=0.014) and between 70 to 79 years (p=0.009).

Table 2: Influence of calendar year and age or frailty on blood pressure thresholds (multilevel analysis)

	β	95% CI	P	
**AGE**†				
Calendar year	1.903	-1.472, 3.278	0.456	<0.001§
Calendar year^2	-0.214	-0.488, 0.059	0.125	
Age <60 years	-10.042	-13.487, -6.597	0.000	
Age 60 – 69 years	-5.803	-9.177, -2.428	0.001	
Age 70 – 79 years	-3.431	-6.932, 0.070	0.055	
Age ≥80 years		reference group		
Interaction year*age		none are significant		
**FRAILTY**‡				
Calendar year	0.838	-0.854, 2.530	0.332	<0.001§
Calendar year^2	-0.237	-0.434, -0.040	0.019	
Frailty 0 – 0.06	-0.110	-1.950, 1.730	0.907	
Frailty 0.08 – 0.11	-0.144	-1.991, 1.703	0.879	
Frailty 0.14 – 0.36		reference group		
Interaction year*frailty		none are significant		

The intraclass correlation coefficient (ICC) calculated from the empty model was 0.037

† the age model was adjusted for sex, duration of diabetes, number of chronic medication at initiation, number and/or type of glucose lowering therapy, lipid lowering therapy, presence of albuminuria, presence of dyslipidaemia, haemoglobin A1C, history of cardiovascular events, estimated glomerular filtration rate and BMI

‡ the frailty model was adjusted for sex, duration of diabetes, number and/or type of glucose lowering therapy, lipid lowering therapy and HbA_{1c}

§ joined significance of calendar year and calendar year^2 using Wald test

Frailty did not influence SBP thresholds for treatment initiation and it did not significantly influence the relationship between calendar year and SBP threshold (Table 2, Figure 2C). In the analyses per frailty group, the SBP level at initiation changed significantly

For peer review only - http://bmjopen.bmj.com/site/about/guidelines.xhtml

(quadratic model) over the years in the least frail (FI 0-0.06; p=0.01) and the most frail (FI 0.14-0.36; p=0.009) patients.

The sensitivity analyses using the mean of the last two SBP levels (Supplementary Figure 1A-C and Supplementary Table 2) and using eFI as a continuous variable (Supplementary Table 3) showed similar results.

Variation between general practices

Of the total variation in SBP level at antihypertensive treatment initiation, 3.7% could be explained by differences between general practices (Table 2, ICC=0.037).

DISCUSSION

Summary

This study shows that, after an initial rise up to 2009, SBP thresholds for antihypertensive treatment initiation decreased over time in a large cohort of patients with T2D treated in primary care. This trend occurred regardless of age and frailty, which was in contrast to our hypothesis given the changes in guideline recommendations. The variation in SBP thresholds for treatment initiation that could be attributed to general practices was small.

Strengths and limitations

The strength of our study is the large number of patients included using real-world data. Furthermore, it is a first study investigating trends in SBP thresholds at initiation of antihypertensive treatment. We focussed on the period from 2007 to 2013, expecting that the shift towards more personalized diabetes care around 2011 could be observed during this period. It is possible that changes occurred in more recent years. First limitation is the fluctuating number of general practices over the years, with different numbers of practices included in each year cohort. Since little variation was explained at practice level, it is unlikely that this affected our primary findings. Second, there were some demographic differences between the patients with and without missing data (Supplementary Table 4). This

For peer review only - http://bmjopen.bmj.com/site/about/guidelines.xhtml

bias was reduced by multiple imputation (25). We could not include smoking as a confounder, however, due to high variability in the amount of missing values over the years. Third, SBP levels show intra-individual variability and may include higher values caused by 'white coat' hypertension (26). However, analysis of the mean last two SBP levels did not change our main findings. Fourth, incomplete coding of ICPC diagnoses in electronic medical records may result in underestimation of frailty.

Comparison with existing literature

The observed trends are not in line with changes in treatment guidelines, where higher thresholds were recommended in the older and/or frail patients, particularly in the later years. Several reasons can explain this discrepancy between our hypothesis and findings. First, Dutch healthcare practitioners may have felt pressured to initiate antihypertensive treatment at lower SBP levels in all diabetes patients after the introduction of performance indicators in 2007 (27). From 2008 onwards, they received yearly feedback on the percentage of patients achieving SBP levels of <140mmHg in their own practice as compared to others practices in the region. Although a previous study in this population did not show an increase in overtreatment after the introduction of performance indicators (28), concerns about the negative impact of such measures have been raised (29). In addition, nurse practitioners increasingly became the pivot of diabetes care (27) and their educational material still recommended a unified SBP target of 140mmHg until 2013 (30). It is also possible that the practitioners did not adhere to treatment guidelines either due to lack of familiarity, understanding or agreement with them (31-33). An interview study conducted in 2015 and 2016, however, suggests that Dutch general practitioners did support the idea of using a higher threshold for initiation of antihypertensive treatment in older and frail patients (13). Nevertheless, we demonstrated that guideline changes were not yet implemented more than two years after being published.

For peer review only - http://bmjopen.bmj.com/site/about/guidelines.xhtml

Antihypertensive treatment was initiated at relatively high SBP levels in patients of all ages but started to decline after 2009, suggesting that it took many years before the United Kingdom Prospective Diabetes Study (UKPDS) recommended threshold of 140 mmHg for treatment initiation was implemented in practice (2). Other studies showed that the percentage of patients achieving an SBP <140mmHg increased in the last two decades but that this increase was smaller in older patients (16). A recent U.S. study showed that the trends in SBP levels in patients ≥60 years decreased until 2010 and remained relatively stable in the six years thereafter (34). Although this study was not restricted to patients with T2D, this seems in contrast with our findings where SBP levels after 2009 decreased in all age groups.

To our surprise, frailty did not influence the SBP threshold for treatment initiation. The frailty range in our study was rather low, which could indicate that antihypertensive treatment is initiated when the patients are still relatively fit. On the other hand, frailty can easily be overlooked due to subtle manifestations, lack of time, or a lack of consensus on the best way to assess it (35). Although the eFI was previously able to identify frailty comparable to the Groningen Frailty Index (22), it might not be in line with the practitioner's perception of a patient's frailty. Therefore, we conducted a post-hoc analysis using the number of chronic medication a patient was receiving at initiation as a proxy for frailty. We observed that the patients being treated with more than three chronic medication initiated treatment at lower SBP levels than those being treated with three or less (Supplementary Figure 2 and Supplementary Table 5). This finding suggests that lower instead of higher thresholds are used for frail patients.

Only a small part of the variation in our study could be attributed to differences between practices. This suggests that patient characteristics determine the threshold to a greater extent than practice characteristics. We only looked at variation between practices which may

For peer review only - http://bmjopen.bmj.com/site/about/guidelines.xhtml

include decisions of two or more general practitioners within one practice. Unfortunately, we could not conduct analyses at the level of individual practitioners.

Conclusion

The observed SBP thresholds at initiation of antihypertensive treatment decreased after 2009. This trend was not influenced by age or frailty, which is in contrast to changes in treatment recommendations. Our study illustrates that changing prescribing practice may take considerable time. Future research is needed to assess the underlying reasons and extent of the delay in the implementation of personalized diabetes care.

For peer review only

ACKNOWLEDGMENTS

Contributorship statement. M.A. contributed to the development and formulation of the research question, conducted the analysis, contributed to the interpretation of data, wrote the

For peer review only - http://bmjopen.bmj.com/site/about/guidelines.xhtml

manuscript, and reviewed and edited the manuscript. S.T.d.V. contributed to the development and formulation of the research question, conducted the analysis, contributed to the interpretation of data, and reviewed and edited the manuscript. G.S. contributed to the development of the analysis, the interpretation of data, and reviewed and edited the manuscript. K.H. contributed to the development and formulation of the research question, the interpretation of data, and reviewed and edited the manuscript P.D. contributed to the development and formulation of the research question, development of the analysis, the interpretation of data, and reviewed and edited the manuscript. M.A. is the guarantor of this work and, as such, takes responsibility for the integrity of the data and the accuracy of the data analysis.

Competing interests. The authors have no conflicts of interest to disclose.

Funding. This project has received funding from the European Union's Horizon 2020 research and innovation programme under the Marie Skłodowska-Curie grant agreement No 754425. The funding source had no involvement in the study.

Data sharing statement. Data are available upon reasonable request.

REFERENCES

(1) Kwaliteitsinstituut voor de Gezondheidszorg, CBO. Multidisciplinaire richtlijn: Cardiovaculair risicomanagement 2006 [Multidisciplinary guidelines: Cardiovasular risk management 2006]. 2006.

For peer review only - http://bmjopen.bmj.com/site/about/guidelines.xhtml

(2) King P, Peacock I, Donnelly R. The UK Prospective Diabetes Study (UKPDS): clinical and therapeutic implications for type 2 diabetes. Br J Clin Pharmacol 1999;48(5):643-648.

(3) American Diabetes Association. Standards of medical care in diabetes 2011. Diabetes Care 2011;34 Suppl 1:11.

(4) Aronow WS, Fleg JL, Pepine CJ, et al. ACCF/AHA 2011 expert consensus document on hypertension in the elderly: a report of the American College of Cardiology Foundation Task Force on Clinical Expert Consensus Documents. Circulation 2011;123(21):2434-2506.

(5) Mancia G, Laurent S, Agabiti-Rosei E, et al. Reappraisal of European guidelines on hypertension management: a European Society of Hypertension Task Force document. J Hypertens 2009;27(11):2121-2158.

(6) Mancia G, Fagard R, Narkiewicz K, et al. 2013 ESH/ESC guidelines for the management of arterial hypertension: the Task Force for the management of arterial hypertension of the European Society of Hypertension (ESH) and of the European Society of Cardiology (ESC). J hypertens 2013;31(7):1281-1357.

(7) Verenso. Multidisciplinaire richtlijn diabetes. Verantwoorde diabeteszorg bij kwetsbare ouderen thuis en in verzorgings of Verpleeghuizen. Deel 1. [Multidisciplinary guideline diabetes. Responsible diabetes care in vulnerable elderly at home and in residential care or nursing homes. Part 1]. Utrecht, the Netherlands: Verenso; 2011.

(8) Hoogendijk E, Theou O, Rockwood K, et al. Development and validation of a frailty index in the Longitudinal Aging Study Amsterdam. Aging Clin Exp Res 2017;29(5):927-933.

(9) Warnes CA, Williams RG, Bashore TM, et al. ACC/AHA 2008 guidelines for the management of adults with congenital heart disease: a report of the American College of Cardiology/American Heart Association task force on practice guidelines (writing committee to develop guidelines on the management of adults with congenital heart disease). Circulation 2008;118(23):e714-e833.

(10) American Diabetes Association. Standards of medical care in diabetes. Diabetes Care 2013;36(1):S11.

(11) Mancia G, De Backer G, Dominiczak A, et al. 2007 guidelines for the management of arterial hypertension: the task force for the management of arterial hypertension of the European Society of Hypertension (ESH) and of the European Society of Cardiology (ESC). J Hypertens 2007;25(6):1105-1187.

(12) Wiersma T, Smulders YM, Stehouwer CDA, et al. Samenvatting van de multidisciplinaire richtlijn 'Cardiovasculair risicomanagement' (herziening 2011) [Recap of the multidisciplinary guidelines Cardiovascular risk management; review 2011] . Nederlands Huisartsen Genootschap 2012;156.

(13) van Middelaar T, Ivens SD, van Peet PG, et al. Prescribing and deprescribing antihypertensive medication in older people by Dutch general practitioners: a qualitative study. BMJ Open 2018;8(4):e020871.

For peer review only - http://bmjopen.bmj.com/site/about/guidelines.xhtml

(14) Andersen UO. Trends in population blood pressure and determinant factors for population blood pressure. Danish medical journal 2017;64(3):B5353.

(15) Nilsson PM, Cederholm J, Zethelius B, et al. Trends in blood pressure control in patients with type 2 diabetes - Data from the Swedish National Diabetes Register (NDR). Blood Press 2011;20(6):348-354.

(16) Sarganas G, Knopf H, Grams D, et al. Trends in antihypertensive medication use and blood bressure control among adults with hypertension in Germany. Am J Hypertens 2016;29(1):104-113.

(17) van Hateren KJ, Drion I, Kleefstra N, et al. A prospective observational study of quality of diabetes care in a shared care setting: trends and age differences (ZODIAC-19). BMJ Open 2012;2(4):10.1136/bmjopen-001387. Print 2012.

(18) Cho YY, Sidorenkov G, Denig P. Role of patient and practice characteristics in variance of treatment quality in type 2 diabetes between general practices. PLoS ONE 2016;11(11):e0166012.

(19) Tran AT, Bakke Å, Berg TJ, et al. Are general practitioners characteristics associated with the quality of type 2 diabetes care in general practice? Results from the Norwegian ROSA4 study from 2014. Scand J Prim Health Care 2018;36(2):170-179.

(20) Cahn A, Raz I, Kleinman Y, et al. Clinical assessment of individualized glycemic goals in patients with type 2 diabetes: formulation of an algorithm based on a survey among leading worldwide diabetologists. Diabetes care 2015;38(12):2293-2300.

(21) Perk J, De Backer G, Gohlke H, et al. European Guidelines on Cardiovascular Disease Prevention in Clinical Practice (Version 2012). Int J Behav Med 2012;19(4):403-488.

(22) Drubbel I, Bleijenberg N, Kranenburg G, et al. Identifying frailty: do the Frailty Index and Groningen Frailty Indicator cover different clinical perspectives? a cross-sectional study. BMC Fam Pract 2013;14(1):64.

(23) Medlon Medischne Diagnostiek. Geschatte GFR: van MDRD naar CKD-EPI [Estimated GFR: from MDRD to CKD-EPI]. 2019; Available at: https://www.medlon.nl/professional/MDRD-naar-CKD-EPI/.

(24) White IR, Royston P, Wood AM. Multiple imputation using chained equations: Issues and guidance for practice. Stat Med 2011;30(4):377-399.

(25) Nguyen DC, John BC, Lee JK. Model checking in multiple imputation: an overview and case study. Emerg Themes Epidemiol 2017;14(1):8-12.

(26) Kerr EA, Zikmund-Fisher BJ, Klamerus ML, et al. The role of clinical uncertainty in treatment decisions for diabetic patients with uncontrolled blood pressure. Ann Intern Med 2008;148(10):717.

(27) Struijs JN, van Til JT, Baan CA. Experimenteren met de keten-dbc diabetes: de eerste zichtbare effecten [Experimenting with a bundled payment system for

For peer review only - http://bmjopen.bmj.com/site/about/guidelines.xhtml

diabetes care in the Netherlands: The first tangible effects]. Bilthoven, the Netherlands: National Institute for Public Health and the Environment (RIVM); 2009.

(28) de Vries ST, Voorham J, Haaijer-Ruskamp FM, et al. Potential overtreatment and undertreatment of diabetes in different patient age groups in primary care after the introduction of performance measures. Diabetes Care 2014;37(5):1312-1320.

(29) O'Connor PJ, Bodkin NL, Fradkin J, et al. Diabetes performance measures: current status and future directions. Diabetes care 2011;34(7):1651-1659.

(30) Houweling ST, Kleefstra N, Holleman F, et al Protocollaire diabeteszorg 2013/2014, mogelijkheden voor taakdelegatie [Protocol for diabetes care 2013/2014, opportunities for task delegation]. 5th ed.: Langerhans; 2013.

(31) Hyman DJ, Pavlik VN. Self-reported hypertension treatment practices among primary care physicians: blood pressure thresholds, drug choices, and the role of guidelines and evidence-based medicine. Arch Intern Med 2000;160(15):2281-2286.

(32) Mehta SS, Wilcox CS, Schulman KA. Treatment of hypertension in patients with comorbidities: Results from the study of hypertensive prescribing practices (SHyPP). Am J Hypertens 1999;12(4):333-340.

(33) Virani SS, Pokharel Y, Steinberg L, et al. Provider understanding of the 2013 ACC/AHA cholesterol guideline. J Clin Lipidol 2015;10(3):497-504.e4.

(34) Dorans KS, Mills KT, Liu Y, et al. Trends in prevalence and control of hypertension according to the 2017 American College of Cardiology/American Heart Association (ACC/AHA) guideline. J Am Heart Assoc 2018;7(11):n/a.

(35) Lee L, Patel T, Hillier LM, et al. Identifying frailty in primary care: A systematic review. Geriatr Gerontol Int 2017;17(10):1358-1377.

FIGURE LEGENDS

Figure 1: American, European, and Dutch guideline recommended systolic blood pressure (BP) values for initiation of antihypertensive treatment in type 2 diabetes patients over the years (1 = American College of Cardiology/American Heart Association (ACC/AHA)

For peer review only - http://bmjopen.bmj.com/site/about/guidelines.xhtml

guidelines (9); 2 = ACC/AHA Expert Consensus Document on Hypertension in the Elderly (4); 3 = American Diabetes Association (ADA) Standards of Medical care in Diabetes (3,10); 4 = European Society of Cardiology/Euro Heart Care (ESC/EHC) guidelines for hypertension management (5,6,11); 5 = Dutch college of General practitioners (NHG) cardiovascular risk management guidelines (1,12); 6 = Verenso multidisciplinary guidelines for the management of diabetes (7))

Figure 2: Mean last systolic blood pressure (BP) value with 95% CIs before/at antihypertensive treatment initiation (A) through the years; (B) through the years in different age groups; (C) through the years in different frailty groups. eFI = electronic frailty index

For peer review only

For peer review only - http://bmjopen.bmj.com/site/about/guidelines.xhtml

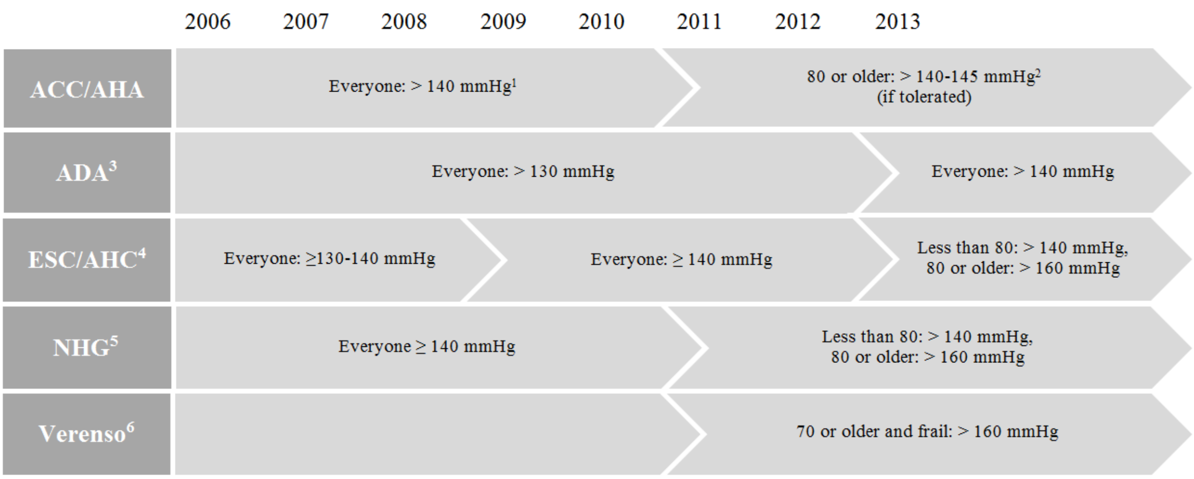

For peer review only

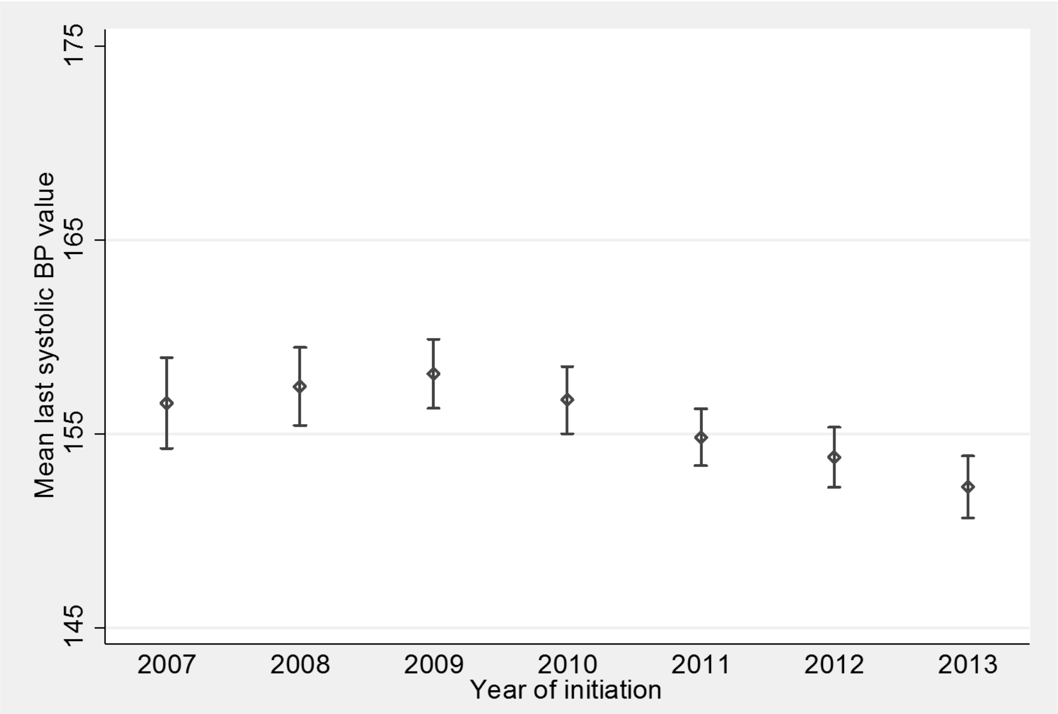
Mean last systolic BP value
145
155
165
175
2007
2008
2009
2010
2011
2012
2013
Year of initiation

(A)

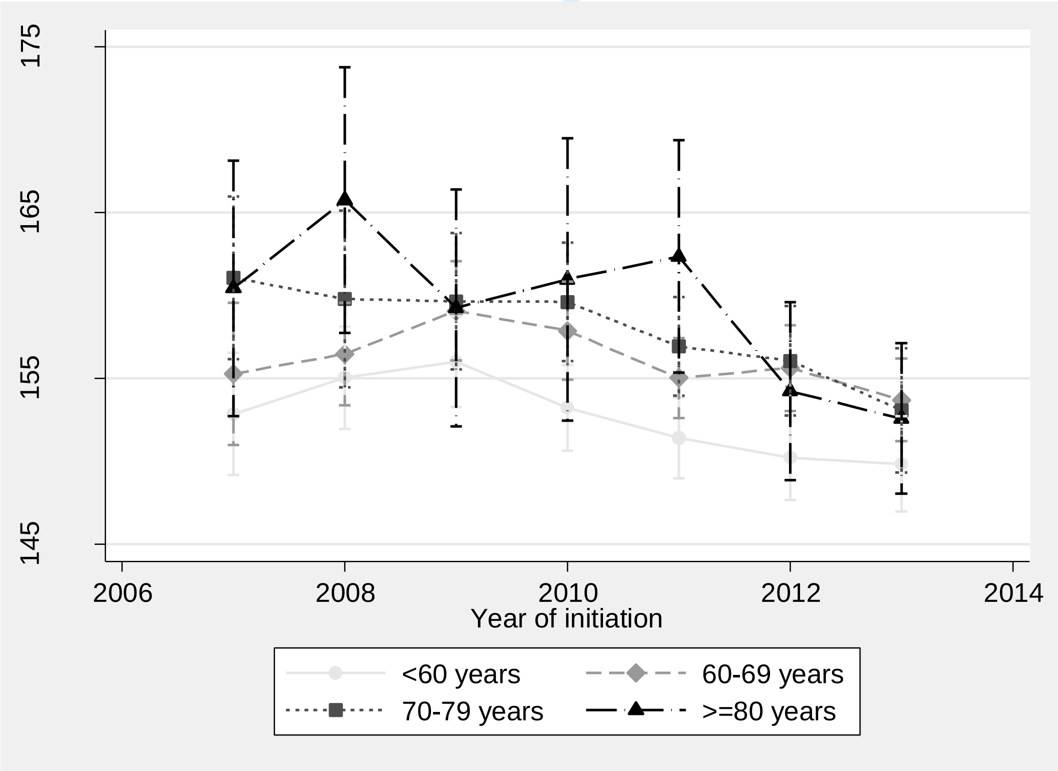
145
155
165
175
2006
2008
2010
2012
2014
Year of initiation
<60 years
60-69 years
70-79 years
>=80 years

(B)

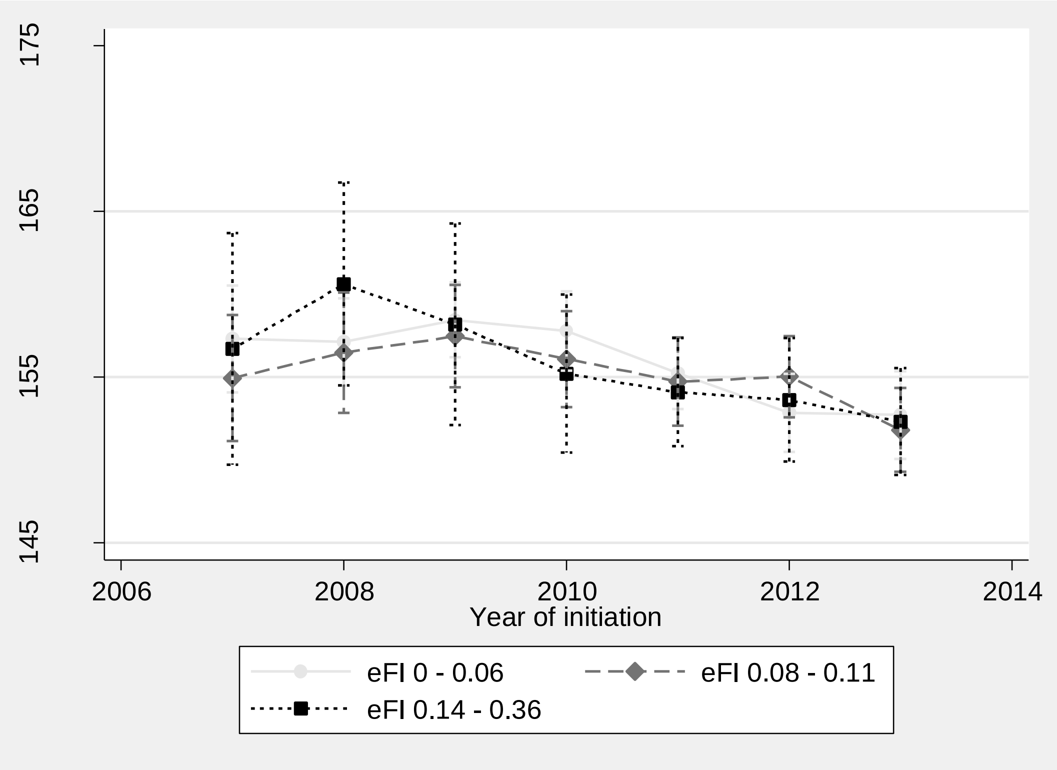

(C)

For peer review only

Supplementary material 1

Supplementary table 1: Characteristics of included patients over the years

	2007	2008	2009	2010	2011	2012	2013
**Number of patients**	328	423	564	591	811	735	718
**Females; N (%)**	154 (47)	178 (42)	270 (48)	277 (47)	360 (44)	355 (48)	355 (49)
**Age in years; N (%)**							
< 60	98 (30)	161 (38)	203 (36)	226 (38)	275 (34)	238 (32)	212 (30)
60 – 69	111 (34)	139 (33)	199 (35)	189 (32)	282 (35)	237 (32)	241 (34)
70 - 79	84 (26)	84 (20)	106 (19)	121 (20)	191 (24)	164 (22)	163 (23)
≥ 80	35 (11)	39 (9)	56 (10)	55 (9)	63 (8)	96 (13)	102 (14)
**Frailty in electronic Frailty Index score; N (%)**							
0 – 0.06	197 (60)	238 (56)	304 (54)	283 (48)	378 (47)	312 (42)	242 (34)
0.08 – 0.11	90 (27)	123 (29)	181 (32)	198 (34)	264 (33)	263 (36)	264 (37)
0.14 – 0.36	41 (13)	62 (15)	79 (14)	110 (19)	169 (21)	160 (22)	212 (30)
**Systolic BP at initiation in mmHg; mean ± SD**	157 ± 22	157 ± 21	158 ± 21	157 ± 22	155 ± 21	154 ± 21	152 ± 22
**Diastolic BP at initiation in mmHg; mean ± SD**	86 ± 12	87 ± 11	87 ± 12	87 ± 11	86 ± 12	85 ± 12	84 ± 12
**Diabetes duration < 2 years; N (%)**	96 (29)	137 (32)	139 (25)	168 (28)	236 (29)	198 (27)	154 (21)
**HbA1c < 7%; N (%)**	150 (59)	232 (61)	308 (59)	323 (60)	497 (64)	454 (65)	418 (60)
**BMI in kg/m^2; N (%)**							
< 24.9	49 (19)	67 (19)	66 (14)	79 (15)	114 (15)	132 (19)	126 (18)
25 – 29.9	100 (39)	142 (39)	218 (46)	222 (42)	312 (40)	280 (40)	283 (41)
> 30	108 (42)	151 (42)	191 (40)	229 (43)	345 (45)	284 (41)	283 (41)
**Dyslipidaemia; N(%)**	92 (46)	161 (50)	232 (55)	269 (58)	418 (60)	403 (62)	366 (57)
**eGFR ≤ 60 ml/min/1.73m2; N (%)**	37 (17)	45 (14)	69 (15)	44 (9)	70 (10)	93 (14)	100 (15)
**Albuminuria (%)**	5 (4)	9 (4)	12 (4)	10 (3)	15 (4)	16 (4)	18 (5)
**History of cardiovascular disease (N (%))**							
Myocardial disease	11 (3%)	21 (5%)	16 (3%)	21 (4%)	31 (4%)	50 (7%)	55 (8%)
Heart failure	5 (2%)	4 (1%)	6 (1%)	6 (1%)	20 (2%)	12 (2%)	15 (2%)
Stroke	13 (4%)	11 (3%)	19 (3%)	20 (3%)	25 (3%)	34 (5%)	45 (6%)

For peer review only - http://bmjopen.bmj.com/site/about/guidelines.xhtml

**Number of chronic medication at initiation; mean ± SD**	3.8 ± 2.3	3.3 ± 2.1	3.4 ± 2.4	3.5 ± 2.2	3.4 ± 2.3	3.7 ± 2.6	3.6 ± 2.5
**Glucose lowering medication at initiation (N (%))**							
No medication	77 (23)	90 (21)	149 (26)	137 (23)	200 (25)	228 (31)	217 (30)
1 oral	129 (39)	179 (42)	199 (35)	247 (42)	363 (45)	271 (37)	283 (39)
2 oral	71 (22)	103 (24)	144 (26)	139 (24)	161 (20)	143 (19)	121 (17)
3 oral or more and/or insulin	51 (16)	51 (12)	72 (13)	68 (12)	87 (11)	93 (13)	97 (14)
**Treated with a lipid lowering drug; N (%)**	219 (67)	255 (60)	298 (53)	343 (58)	447 (55)	417 (57)	408 (57)
**Initiated drug class; N (%)**							
Renin-angiotensin-aldosterone system inhibitor	185 (60)	241 (61)	330 (63)	319 (60)	453 (62)	415 (63)	379 (60)
Diuretic	59 (19)	73 (18)	101 (19)	104 (20)	123 (17)	108 (16)	89 (14)
Beta blocker	51 (17)	59 (15)	75 (14)	80 (15)	117 (16)	93 (14)	116 (18)
Calcium channel blocker	14 (5)	22 (6)	21 (4)	29 (5)	32 (4)	40 (6)	44 (7)
Combination of antihypertensives	19 (6)	28 (7)	37 (7)	59 (10)	86 (11)	79 (11)	90 (13)

review only

Supplementary material 2

Sensitivity analysis using mean of last two blood pressure measurements (N =2599)

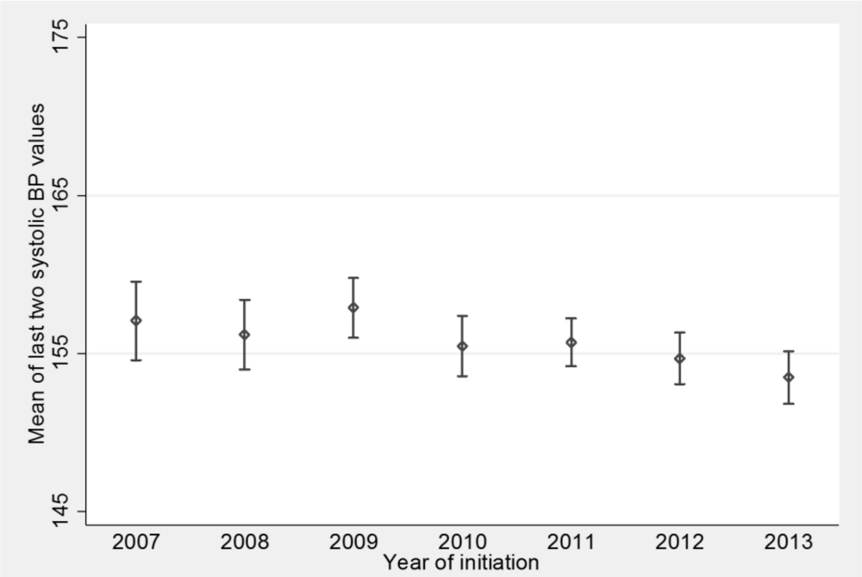

(A)

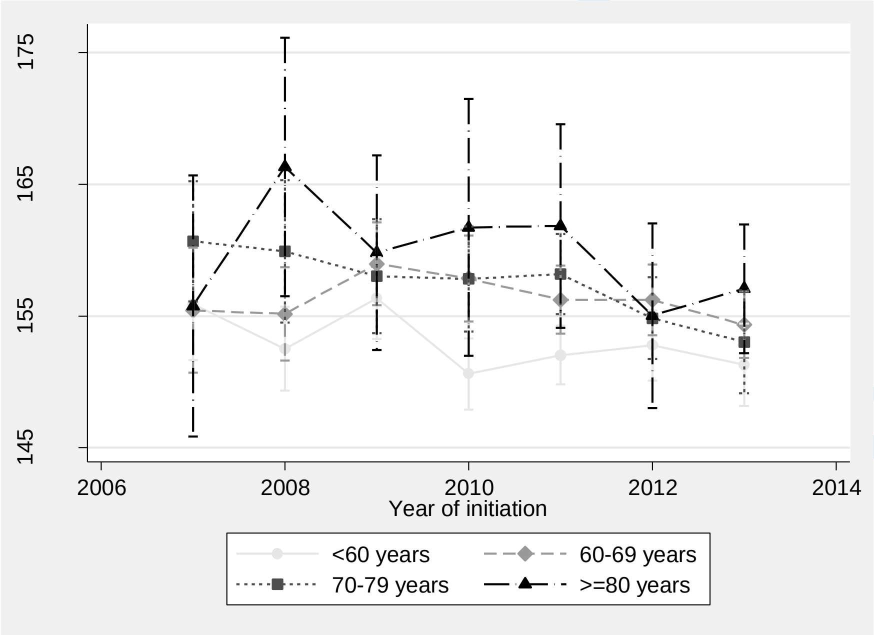

(B)

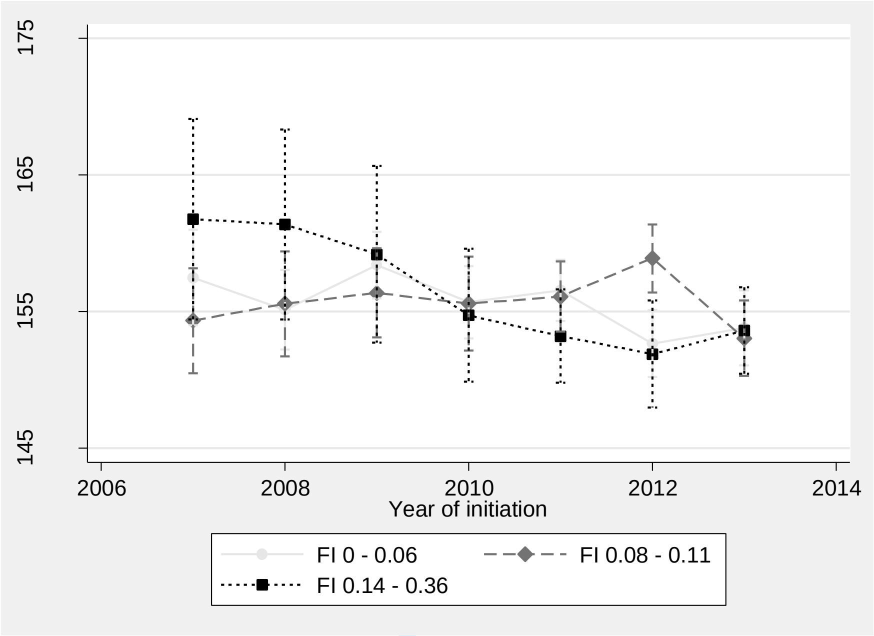

(C)

Supplementary figure 1: Mean of last two systolic blood pressure (BP) levels with 95% CIs before/at antihypertensive treatment initiation (A) through the years; (B) through the years in different age groups and (C) through the years in different frailty groups.

peer review only

Supplementary table 2: Influence of calendar year and age or frailty on blood pressure thresholds (multilevel analysis) using the mean of last two systolic blood pressure measurements

	β	95% CI	P	
**AGE***				
Calendar year	0.930	-1.525, 3.385	0.458	0.0011‡
Calendar year^2	-0.200	-0.480, 0.080	0.163	
Age <60 years	-9.873	-13.466, -6.280	0.000	
Age 60 – 69 years	-6.086	-9.598, -2.575	0.001	
Age 70 – 79 years	-4.363	-8.006, -0.720	0.019	
Age ≥80 years		reference group		
Interaction year*age		none are significant		
**FRAILTY†**				
Calendar year	-0.193	-1.985, 1.599	0.833	<0.001‡
Calendar year^2	-0.088	-0.296, 0.119	0.404	
Frailty 0 – 0.06	0.124	-1.780, 2.028	0.898	
Frailty 0.08 – 0.11	0.844	-1.0731, 2.760	0.388	
Frailty 0.14 – 0.36		reference group		
Interaction year*frailty		none are significant		

The intraclass correlation coefficient (ICC) calculated from the empty model was 0.057.
* the age model was adjusted for sex, duration of diabetes, number of chronic medication at initiation, number and/or type of glucose lowering therapy, lipid lowering therapy, presence of albuminuria, presence of dyslipidaemia, haemoglobin A1C, history of cardiovascular events, estimated glomerular filtration rate and BMI
† the frailty model was adjusted for sex, duration of diabetes, number and/or type of glucose lowering therapy, lipid lowering therapy and haemoglobin A1C
‡ joined significance of calendar year and calendar year^2 using Wald test

iew only

Supplementary material 3

Sensitivity analysis using electronic frailty index as a continuous variable

There were no statistically significant differences in blood pressure thresholds between patients with different frailty (Supplementary table 3).

Supplementary table 3: Influence of calendar year and frailty on blood pressure thresholds (multilevel analysis), using frailty index as a continuous variable

	β	95% CI	P	
**FRAILTY**[†]				
Calendar year	0.825	-0.867, 2.517	0.339	<0.001[‡]
Calendar year^2	-0.229	-0.427, -0.032	0.023	
Frailty	-8.713	-21.676, 4.249	0.188	
Interaction year*frailty		none are significant		

[†] the frailty model was adjusted for sex, duration of diabetes, number and/or type of glucose lowering therapy, lipid lowering therapy and haemoglobin A1C

[‡] joined significance of calendar year and calendar year^2 using Wald test

For peer review only

Supplementary material 4

Supplementary table 4: Characteristics of included patients: comparison of complete cases and cases with missing values

	complete cases	cases with missings
**Patients; N**	3 017	1 153
**Females; N (%)**	1 361 (45%)	588 (51%)
**Age in years; mean ± SD**	64 ± 11	65 ± 13
**Frailty index; median (Q1 - Q3)**	0.08 (0.06 – 0.11)	0.08 (0.03 – 0.11)
**SBP at initiation in mmHg; mean ± SD**	154 ± 21	158 ± 23
**DBP at initiation in mmHg; mean ± SD**	85 ± 11	87 ± 13
**Diabetes duration in years; mean ± SD**	5.3 ± 5.3	5.2 ± 5.2
**N of chronic medication at initiation; mean ± SD**	3.6 ± 2.3	3.4 ± 2.5
**Treated with a lipid lowering drug; N (%)**	1 829 (61%)	558 (48%)
**Initiated with one antihypertensive; N (%)**	2 747 (91%)	1 025 (89%)

For peer review only

Supplementary material 5

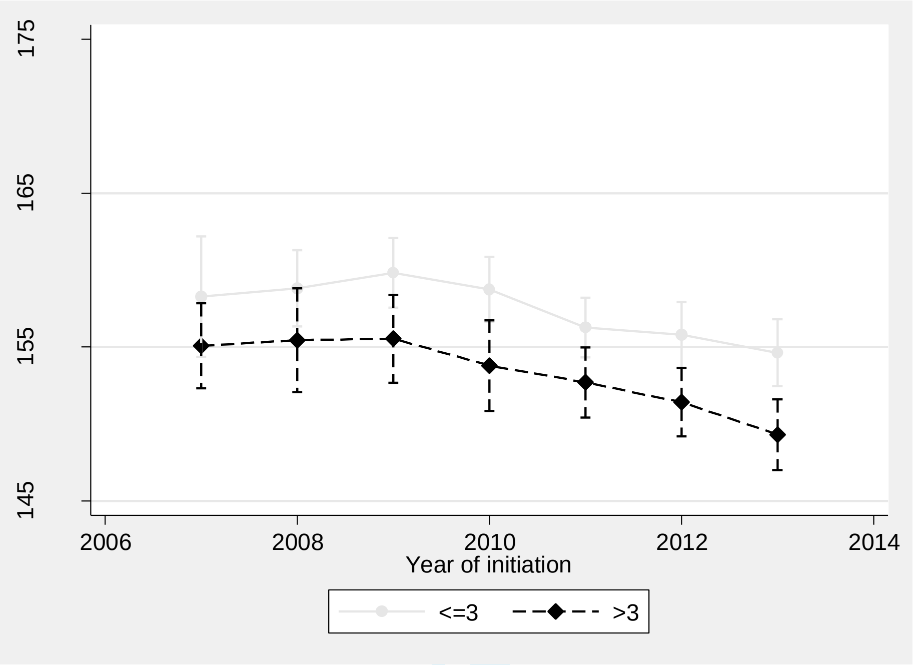

Supplementary figure 2: Mean last systolic blood pressure value with 95% CIs before/at antihypertensive treatment initiation through the years in patients with different number of chronic medication at initiation.

Supplementary table 5: Multilevel analysis of number of chronic medication at initiation

	β	95% CI (min, max)	P	
**Calendar year**	0.630	-1.774, 3.034	0.505	0.0002‡
**Calendar year^2**	-0.176	-0.453, 0.100	0.141	
**3 or less chronic medication**		reference group		
**More than 3 chronic medication**	-3.421	-5.356, -1.486	0.001	
**Interaction year*N of medication**		none are significant		

The model was adjusted for sex, duration of diabetes, number and/or type of glucose lowering therapy, lipid lowering therapy, presence of albuminuria, presence of dyslipidaemia, haemoglobin A1C, estimated glomerular filtration rate and BMI.

‡ joined significance of calendar year and calendar year^2 using Wald test

BMJ Open

Changes in blood pressure thresholds for initiating antihypertensive medication in diabetes patients: A repeated cross-sectional study focusing on the impact of age and frailty

Journal:	*BMJ Open*
Manuscript ID	bmjopen-2020-037694.R1
Article Type:	Original research
Date Submitted by the Author:	23-Jun-2020
Complete List of Authors:	Ambrož, Martina; University Medical Centre Groningen,<br>de Vries, Sieta; University Medical Centre Groningen, Clinical Pharmacy and Pharmacology<br>Sidorenkov, Grigory; University Medical Centre Groningen, Epidemiology<br>Hoogenberg, Klaas; Martini Ziekenhuis<br>Denig, Petra; University Medical Centre Groningen, Clinical Pharmacy & Pharmacology
**Primary Subject Heading**:	Diabetes and endocrinology
Secondary Subject Heading:	Cardiovascular medicine, Epidemiology, Evidence based practice, General practice / Family practice, Pharmacology and therapeutics
Keywords:	General diabetes < DIABETES & ENDOCRINOLOGY, PRIMARY CARE, Hypertension < CARDIOLOGY, EPIDEMIOLOGY, GERIATRIC MEDICINE, Protocols & guidelines < HEALTH SERVICES ADMINISTRATION & MANAGEMENT

SCHOLARONE™
Manuscripts

BMJ

I, the Submitting Author has the right to grant and does grant on behalf of all authors of the Work (as defined in the below author licence), an exclusive licence and/or a non-exclusive licence for contributions from authors who are: i) UK Crown employees; ii) where BMJ has agreed a CC-BY licence shall apply, and/or iii) in accordance with the terms applicable for US Federal Government officers or employees acting as part of their official duties; on a worldwide, perpetual, irrevocable, royalty-free basis to BMJ Publishing Group Ltd ("BMJ") its licensees and where the relevant Journal is co-owned by BMJ to the co-owners of the Journal, to publish the Work in this journal and any other BMJ products and to exploit all rights, as set out in our licence.

The Submitting Author accepts and understands that any supply made under these terms is made by BMJ to the Submitting Author unless you are acting as an employee on behalf of your employer or a postgraduate student of an affiliated institution which is paying any applicable article publishing charge ("APC") for Open Access articles. Where the Submitting Author wishes to make the Work available on an Open Access basis (and intends to pay the relevant APC), the terms of reuse of such Open Access shall be governed by a Creative Commons licence – details of these licences and which Creative Commons licence will apply to this Work are set out in our licence referred to above.

Other than as permitted in any relevant BMJ Author's Self Archiving Policies, I confirm this Work has not been accepted for publication elsewhere, is not being considered for publication elsewhere and does not duplicate material already published. I confirm all authors consent to publication of this Work and authorise the granting of this licence.

For peer review only

Changes in blood pressure thresholds for initiating antihypertensive medication in diabetes patients: A repeated cross-sectional study focusing on the impact of age and frailty

Martina Ambrož, MPharm[a]; Sieta T. de Vries, PhD[a]; Grigory Sidorenkov, PhD[b]; Klaas Hoogenberg, MD, PhD[c]; Petra Denig, PhD[a]

[a] Department of Clinical Pharmacy and Pharmacology, University of Groningen, University Medical Center Groningen, Hanzeplein 1, 9713 GZ, Groningen, The Netherlands

[b] Department of Epidemiology, University of Groningen, University Medical Center Groningen, Hanzeplein 1, 9713 GZ, Groningen, The Netherlands

[c] Department of Internal Medicine, Martini Hospital, Van Swietenplein 1, 9728 NT, Groningen, the Netherlands

Declarations of interest: none

Corresponding author: Martina Ambrož, PO Box 30.001, 9700 RB Groningen, +31 613914263, m.ambroz@umcg.nl

ORCID:

Martina Ambrož https://orcid.org/0000-0003-1319-4898

Sieta T. de Vries https://orcid.org/0000-0001-6090-2434

Grigory Sidorenkov https://orcid.org/0000-0002-1926-8862

Petra Denig https://orcid.org/0000-0002-7929-4739

Word count: 2 733

For peer review only - http://bmjopen.bmj.com/site/about/guidelines.xhtml

ABSTRACT

Objective

To assess trends in systolic blood pressure (SBP) thresholds at initiation of antihypertensive treatment in type 2 diabetes (T2D) patients and the impact of age and frailty on these trends.

Study Design and Setting

A repeated cross-sectional cohort study (2007-2014) using the Groningen Initiative to Analyze Type 2 diabetes Treatment (GIANTT) database was conducted. The influence of calendar year, age or frailty and the interaction between year and age or frailty on SBP thresholds were assessed using multilevel regression analyses adjusted for potential confounders.

Results

We included 4 819 patients. The mean SBP at treatment initiation was 157mmHg in 2007, rising to 158mmHg in 2009 and decreasing to 151mmHg in 2014. This quadratic trend was significant ($p<0.001$). Older patients initiated treatment at higher SBP, but similar decreasing trends after 2009 were observed in all age groups. There were no significant differences in SBP thresholds between patients with different frailty. The association between year and SBP threshold was not influenced by age or frailty.

Conclusion

After an initial rise, the observed SBP thresholds decreased over time and were not influenced by age or frailty. This is in contrast with changed guideline recommendations toward more personalized treatment during the study period and illustrates that changing prescribing practice may take considerable time. Patient-specific algorithms and tools focusing on when and when not to initiate treatment could be helpful to support personalized diabetes care.

Key words: diabetes mellitus, type 2; blood pressure; primary health care; drug utilization; elderly; frail

For peer review only

Strengths and limitations:

- Large number of patients
- Real-world data from primary care
- Study period focused on the time when guidelines became more personalized
- Changes in prescribing behavior might have happened after our study period
- Frailty might have been underestimated

For peer review only

For peer review only - http://bmjopen.bmj.com/site/about/guidelines.xhtml

INTRODUCTION

Treatment of hypertension in patients with type 2 diabetes (T2D) reduces their cardiovascular risk, but guideline recommendations on when to initiate antihypertensive treatment to best balance the benefits and risks of treatment have changed over time. In the past, the recommended systolic blood pressure (SBP) threshold for treatment initiation ranged from 130mmHg to 140mmHg (1-7). A personalized approach, however, has been advocated in the last decade for older and/or frail patients, who are at increased risk of adverse outcomes related to low BP levels (4,6,7). Since 2011, treatment guidelines started to recommend higher SBP thresholds in these patients (Figure 1; 3-11). A recent interview study showed that Dutch general practitioners were indeed somewhat reluctant to initiate antihypertensive treatment in older and/or frail patients (12). Studies showing at which SBP thresholds physicians initiate antihypertensive treatment in older or frail patients are lacking.

A Danish study observed an average SBP level in the general population of 148mmHg before they received antihypertensive treatment in the period from 1976 to 2004 (13). Trend studies on antihypertensive medication use and hypertension control in individuals with T2D show that the percentage of people achieving the recommended SBP target of <140mmHg increased over the last twenty years (14,15). An observational study conducted in the Netherlands showed that the mean achieved SBP decreased from 155mmHg in 1998 to 140mmHg in 2008 in all age groups, with a mean SBP being lower in younger patients. No relevant differences in trends were observed between different age groups (16). This indicates that risk factor control in patients with T2D has generally improved over time. The extent to which the more recent personalized guideline recommendations are followed, however, is not clear. This may depend on physician or practice characteristics (17,18), resulting in variability between treatment decisions (19).

For peer review only - http://bmjopen.bmj.com/site/about/guidelines.xhtml

The aim of this study was to assess trends in SBP thresholds for initiating antihypertensive medication in patients with T2D and the impact of changed treatment recommendations for older and frail patients. We looked at the period between 2007 and 2014, for which we hypothesized that SBP thresholds would remain similar among young and non-frail patients but would increase among older and/or frail patients. Our secondary aim was to assess to what extent SBP thresholds for treatment initiation varied across general practices.

METHODS

Study design and population

This was a repeated cross-sectional dynamic cohort study for the years 2007 to 2014. The Groningen Initiative to Analyse Type-2 diabetes Treatment (GIANTT; www.giantt.nl) database was used, which contains anonymous electronic medical records data of patients with T2D treated in primary care in the north of the Netherlands.

Patients were included per calendar year when they had a diagnosis of T2D and were ≥18 years. We excluded patients who were not included in the database for at least 365 days before antihypertensive treatment initiation and did not initiate treatment with an antihypertensive (Anatomical Therapeutic Chemical (ATC) codes C03, C04, C07, C08, C09) in that year. Antihypertensive treatment initiation was defined as an initial prescription without a prescription of any antihypertensive drug in the preceding 365 days. Furthermore, patients were excluded when they did not have a documented SBP level within 120 days before or at the day of treatment initiation or when they initiated treatment with three or more drug classes, since it is unlikely that this was a true initiation. It is assumed that these are patients with prevalent antihypertensive treatment, who entered the dynamic cohort during the study period. Moreover, we excluded patients initiating propranolol or a loop diuretic (furosemide, bumetanide), since these are commonly prescribed for other indications (i.e

For peer review only - http://bmjopen.bmj.com/site/about/guidelines.xhtml

migraine prophylaxis or short-term use in patients with edema, respectively). No approval from an ethics committee is needed for studies using data from anonymous medical records in the Netherlands. An exemption letter from University Medical Center Groningen Medical Ethics Review Board was obtained (reference number M19.235285).

Outcome variable

The outcome was the patient's most recent office SBP level in the 120 days before or on the day of antihypertensive treatment initiation.

Explanatory variables

The following explanatory variables were included: calendar year, age or frailty of the patient and the interaction between year and age or frailty. Age was calculated on January 1 of each year and was categorized in four groups (<60 years, 60–69 years, 70–79 years and ≥80 years) related to cut-off values mentioned in several guidelines (7,11,20). Frailty was calculated using a previously developed electronic frailty index (eFI), which is based on 140 International Classification of Primary Care (ICPC) coded symptoms and diagnoses from the medical history as well as the existence of polypharmacy (21). These ICPC codes are grouped into 36 deficits, for which patients get points. For chronic conditions, a diagnostic code anytime in the past is included, whereas for short-term or episodic conditions only diagnostic codes from the past year are included. The sum of the points from the deficits divided by 36 is the indication of frailty and can take a value between 0 (patient has no deficits) and 1 (patient has all possible deficits). Since all included patients had diabetes, we excluded diabetes from the eFI, thus focusing on additional frailty. There are no validated clinical cut-off values for the eFI, therefore, we categorized the scores in tertiles based on the eFI values in our study population to compare less-, medium- and more frail patients.

Confounders

For peer review only - http://bmjopen.bmj.com/site/about/guidelines.xhtml

The following patient characteristics were included as possible confounders: sex, diabetes duration (<2 years or ≥2 years), presence of dyslipidemia (LDL ≥2.5 mmol/L), glycated hemoglobin level (HbA_{1c} <7% or ≥7%), estimated glomerular filtration rate (eGFR; ≤60 mL/min or >60 mL/min), presence of elevated albuminuria (albumin creatinine ratio ≥30 mg/g or albumin in 24h urine ≥300 mg), history of cardiovascular events (presence yes/no of myocardial disease, heart failure or stroke), body mass index (BMI; <24.9 kg/m^2, 25–29.9 kg/m^2 or ≥30 kg/m^2), number of prescribed chronic medication (continuous variable), number and type of glucose lowering treatment (none, one oral, two oral, three or more oral and/or insulin), and lipid lowering treatment (none or one/more drug classes). The most recent laboratory values available in the 365 days before or seven days after treatment initiation were used. BMI was calculated from weight and height or extracted from the database in case these were not available. The eGFR was calculated from serum creatinine using the Modification of Diet in Renal Disease-4 equation for the years 2007 to 2009, and using the Chronic Kidney Disease Epidemiology Collaboration equation from 2010 onwards, since the standard way of calculating eGFR in the Netherlands changed during the study period (22). In case serum creatinine was not available, the eGFR was extracted from the database when available. Prescribed medication was assessed in the 120 days before or at the day of treatment initiation.

Missing data

There were no missing data for the explanatory variables. Values of confounders with <20% of missing values were imputed using multiple imputation by chained equation (23). For patients without albuminuria measurements (47%), we assumed that they did not have elevated albuminuria, since urine samples were less likely to be collected in our study period for patients without suspected renal function problems.

Analyses

For peer review only - http://bmjopen.bmj.com/site/about/guidelines.xhtml

Descriptive statistics were performed to examine patient characteristics per calendar year.

We conducted multilevel regression analyses with a two-level random intercept model to account for patients being nested within general practices. First, using the empty model, which includes only the outcome variable, we calculated the intraclass correlation coefficient to assess the variance that is attributed to general practices. Second, we added the confounders to assess the overall trend over the years. We compared a linear and a quadratic model using the Wald test to choose the best fitting final model. In the final model we assessed the effect of age or frailty on the trends by adding the explanatory variables and the interaction between year and age or frailty on SBP levels at treatment initiation.

Additional subgroup analyses were conducted for each age and frailty group to assess changes over time in these subpopulations using the final model. After applying Bonferroni correction for multiple testing, significance levels were set at $p<0.0125$ (per age group) and $p<0.0167$ (per frailty group).

Furthermore, we conducted sensitivity analyses using the average of the last two SBP levels instead of a single SBP measurement and using eFI as a continuous variable in the final model.

The analyses were conducted in Stata version 14 (Stata Corp., College Station, TX).

Patient and public involvement

Patients and public were not involved in this study.

RESULTS

A total of 4 819 patients initiating antihypertensive treatment in the period 2007 to 2014 were included (Table 1). A flow chart of excluded patients per calendar year is presented in Supplementary Figure 1. Patient characteristics were generally similar throughout the years (Supplementary Table1). Seventy-four percent of included patients had no missing values.

For peer review only - http://bmjopen.bmj.com/site/about/guidelines.xhtml

Table 1: Characteristics of included patients (N = 4 819)

**Calendar year**		**Included patients**
	2007	328
	2008	423
	2009	564
	2010	591
	2011	811
	2012	735
	2013	718
	2014	649
**Females; N (%)**		2 259 (47)
**Age in years; N (%)**		
	< 60	1620 (34)
	60 – 69	1 585 (33)
	70 – 79	1 068 (22)
	≥ 80	546 (11)
**Frailty in electronic Frailty Index score; N (%)**		
	Less frail: 0 – 0.03	2 070 (43)
	Medium frail: 0.06 – 0.09	1 628 (34)
	More frail: 0.11 – 0.40	1 121 (23)
**Systolic BP at initiation in mmHg; mean ± SD**		155 ± 22
**Diastolic BP at initiation in mmHg; mean ± SD**		85 ± 12
**Diabetes duration <2 years; N (%)**		1 259 (26)
**HbA_{1c} < 7%; N (%)** †		2 717 (61)
**BMI in kg/m^2; N (%)** ‡		
	< 24.9	751 (17)
	25 – 29.9	1 788 (41)
	≥ 30	1 853 (42)
**Dyslipidaemia; N (%)** §		2 263 (57)
**eGFR ≤ 60 ml/min/1.73m2; N (%)** ¶		542 (13)
**Elevated albuminuria (%)** ‖		101 (4)
**History of cardiovascular disease; N (%)**		
	Myocardial disease[1]	263 (5)
	Heart failure[2]	90 (2)
	Stroke[3]	212 (4)
**Number of chronic medication at initiation; mean ± SD**		3.6 ± 2.5
**Glucose lowering medication at initiation; N (%)**		
	No medication	1 269 (26)
	1 oral	1 937 (40)
	2 oral	982 (21)
	3 oral or more and/or insulin	631 (13)
**Treated with a lipid lowering drug; N (%)**		2 749 (57)
**Initiated drug class; N (%)**		
	Renin-angiotensin-aldosterone system inhibitor	2 645 (55)
	Diuretic	762 (16)
	Beta blocker	689 (14)
	Calcium channel blocker	240 (5)
	Combination of antihypertensives	474 (10)

For peer review only - http://bmjopen.bmj.com/site/about/guidelines.xhtml

†hemoglobin A1C (HbA_{1c}): 352 (7.3 %) missing values; ‡BMI: 427 (8.9 %) missing values; § LDL-cholesterol: 874 (18.1 %) missing values; ¶ estimated glomerular filtration rate (eGFR): 677 (14.0 %) missing values; || albuminuria: 2 274 (47.2%) missing values; [1] acute myocardial infarction (ICPC code K75) in the last year or other/chronic ischaemic heart disease (ICPC code K76) anytime in history; [2] heart failure (ICPC code K77) anytime in history; [3] transient cerebral ischemia (ICPC code K89) in the last year or stroke/cerebrovascular incident (ICPC code K90) anytime in history

Trends in SBP thresholds

The mean SBP level at antihypertensive treatment initiation significantly changed over time from 157mmHg (SD 22mmHg) in 2007, rising to 158mmHg (SD 21mmHg) in 2009 and thereafter decreasing to 151mmHg (SD 22mmHg) in 2014 (Figure 2A). This quadratic trend was statistically significant (p<0.001).

Age and frailty

Older patients initiated treatment at significantly higher SBP thresholds than younger patients but age did not significantly influence the relationship between calendar year and SBP threshold (Table 2, Figure 2B). In the analyses per age group, the SBP level at initiation changed significantly (quadratic model) over the years in patients aged between 60 and 69 years (p=0.001).

Table 2: Influence of calendar year and age or frailty on blood pressure thresholds (multilevel analysis)

	β	95% CI	P	
**AGE†**				
Calendar year	-0.107	-1.429, 1.215	0.874	<0.001§
Calendar year²	-0.111	-0.248, 0.027	0.114	
Age <60 years	-8.066	-10.411, -5.723	<0.001	
Age 60 – 69 years	-4.115	-6.369, -1.861	<0.001	
Age 70 – 79 years	-1.168	-3.407, 1.072	0.307	
Age ≥80 years		reference group		
Interaction year*age		none are significant		
**FRAILTY‡**				
Calendar year	0.247	-1.100, 1.593	0.719	<0.001§
Calendar year²	-0.159	-0.299, -0.018	0.027	
Frailty 0 – 0.03	-0.060	-1.734, 1.614	0.944	
Frailty 0.06 – 0.09	0.127	-1.519, 1.772	0.880	
Frailty 0.11 – 0.40		reference group		
Interaction year*frailty		none are significant		

The intraclass correlation coefficient (ICC) calculated from the empty model was 0.037
† the age model was adjusted for sex, duration of diabetes, number of chronic medication at initiation, number and/or type of glucose lowering therapy, lipid lowering therapy, presence of

For peer review only - http://bmjopen.bmj.com/site/about/guidelines.xhtml

albuminuria, presence of dyslipidaemia, haemoglobin A1C, history of cardiovascular events, estimated glomerular filtration rate and BMI
‡ the frailty model was adjusted for sex, duration of diabetes, number and/or type of glucose lowering therapy, lipid lowering therapy and HbA_{1c}
§ joined significance of calendar year and calendar $year^2$ using Wald test

Frailty did not influence SBP thresholds for treatment initiation and it did not significantly influence the relationship between calendar year and SBP threshold (Table 2, Figure 2C). In the analyses per frailty group, the SBP level at initiation changed significantly (quadratic model) over the years in the less frail (eFI 0-0.03; $p<0.001$) and more frail (eFI 0.11-0.40; $p=0.001$) patients.

The sensitivity analyses using the mean of the last two SBP levels (Supplementary Figure 2A-C and Supplementary Table 2) and using eFI as a continuous variable (Supplementary Table 3) showed similar results.

Variation between general practices

Of the total variation in SBP level at antihypertensive treatment initiation, 3.2% could be explained by differences between general practices (Table 2, ICC=0.032).

DISCUSSION

Summary

This study shows that, after an initial rise up to 2009, SBP thresholds for antihypertensive treatment initiation decreased over time in a large cohort of patients with T2D treated in primary care. This trend occurred regardless of age and frailty, which was in contrast to our hypothesis given the changes in guideline recommendations. The variation in SBP thresholds for treatment initiation that could be attributed to general practices was small.

Strengths and limitations

The strength of our study is the large number of patients included using real-world data. Furthermore, it is a first study investigating trends in SBP thresholds at initiation of antihypertensive treatment. We focussed on the period from 2007 to 2014, expecting that the

For peer review only - http://bmjopen.bmj.com/site/about/guidelines.xhtml

shift towards more personalized diabetes care around 2011 could be observed during this period. It is possible that changes occurred in more recent years. The first limitation is the fluctuating number of general practices over the years, with different numbers of practices included in each year cohort. Since little variation was explained at practice level, it is unlikely that this affected our primary findings. Second, there were some demographic differences between the patients with and without missing data (Supplementary Table 4). This bias was reduced by multiple imputation (24). Third, we could not include smoking or date of hypertension diagnosis as confounders due to amount and variability of missing values over the years. Furthermore, ICPC codes do not provide information about the severity of the comorbidities. Therefore, there may be some residual confounding which was not accounted for. Fourth, incomplete coding of ICPC diagnoses in electronic medical records may result in underestimation of frailty. Finally, SBP levels show intra-individual variability and may include higher values caused by 'white coat' hypertension (25). However, analysis of the mean last two SBP levels did not change our main findings.

Comparison with existing literature

The observed trends are not in line with changes in treatment guidelines, where higher thresholds were recommended in the older and/or frail patients, particularly in the later years. Several reasons can explain this discrepancy between our hypothesis and findings. First, Dutch healthcare practitioners may have felt pressured to initiate antihypertensive treatment at lower SBP levels in all diabetes patients after the introduction of performance indicators in 2007 (26). From 2008 onwards, they received yearly feedback on the percentage of patients achieving SBP levels of <140mmHg in their own practice as compared to other practices in the region. Although a previous study in this population did not show an increase in overtreatment after the introduction of performance indicators (27), concerns about the negative impact of such measures have been raised (28). In addition, nurse practitioners

For peer review only

increasingly became the pivot of diabetes care (26) and their educational material recommended a unified SBP target of 140mmHg at least until the end of our study period (29). It is also possible that the practitioners did not adhere to treatment guidelines either due to lack of familiarity, understanding or agreement with them (30-32). An interview study conducted in 2015 and 2016, however, suggests that Dutch general practitioners did support the idea of using a higher threshold for initiation of antihypertensive treatment in older and frail patients (12). Nevertheless, we demonstrated that guideline changes were not yet implemented more than three years after being published.

Antihypertensive treatment was initiated at relatively high SBP levels in patients of all ages but started to decline after 2009, suggesting that it took many years before the United Kingdom Prospective Diabetes Study (UKPDS) recommended threshold of 140mmHg for treatment initiation was implemented in practice (2). Other studies showed that the percentage of patients achieving an SBP <140mmHg increased in the last two decades but that this increase was smaller in older patients (15). A recent U.S. study showed that the trends in SBP levels in patients ≥60 years decreased until 2010 and remained relatively stable in the six years thereafter (33). Although this study was not restricted to patients with T2D, this seems in contrast with our findings where SBP levels after 2009 decreased in all age groups.

To our surprise, frailty did not influence the SBP threshold for treatment initiation. The frailty range in our study was rather low, which could indicate that antihypertensive treatment is initiated when the patients are still relatively fit. On the other hand, frailty can easily be overlooked due to subtle manifestations, lack of time, or a lack of consensus on the best way to assess it (34). Although the eFI was previously able to identify frailty comparable to the Groningen Frailty Index (21), it might not be in line with the practitioner's perception of a patient's frailty. Therefore, we conducted a post-hoc analysis using the number of chronic medication a patient was receiving at initiation as a proxy for frailty. We observed that the

For peer review only - http://bmjopen.bmj.com/site/about/guidelines.xhtml

For peer review only

patients being treated with more than three chronic medication (median) initiated treatment at lower SBP levels than those being treated with three or less (Supplementary Figure 3 and Supplementary Table 5). This finding suggests that lower instead of higher thresholds are used for frail patients.

Only a small part of the variation in our study could be attributed to differences between practices. This suggests that patient characteristics determine the threshold to a greater extent than practice characteristics. We only looked at variation between practices which may include decisions of two or more general practitioners within one practice. Unfortunately, we could not conduct analyses at the level of individual practitioners.

Conclusion and implications

The observed SBP thresholds at initiation of antihypertensive treatment decreased after 2009. This trend was not influenced by age or frailty, which is in contrast to changes in treatment recommendations. Our study illustrates that changing prescribing practice may take considerable time and only publishing new recommendations might not be sufficient for successful implementation. On the one hand, patient-specific algorithms and tools to support the timely start of antihypertensive treatment in younger patients are needed. On the other hand, also algorithms and tools to prevent the initiation of too early or strict antihypertensive treatment in older and frail patients should be developed. Further research is needed to assess the underlying reasons and extent of the delay in the implementation of personalized diabetes care and evaluate the impact of strategies to speed up the uptake of recommendations.

For peer review only

For peer review only

For peer review only - http://bmjopen.bmj.com/site/about/guidelines.xhtml

ACKNOWLEDGMENTS

Contributorship statement. M.A. contributed to the development and formulation of the research question, conducted the analysis, contributed to the interpretation of data, wrote the manuscript, and reviewed and edited the manuscript. S.T.d.V. contributed to the development and formulation of the research question, conducted the analysis, contributed to the interpretation of data, and reviewed and edited the manuscript. G.S. contributed to the development of the analysis, the interpretation of data, and reviewed and edited the manuscript. K.H. contributed to the development and formulation of the research question, the interpretation of data, and reviewed and edited the manuscript P.D. contributed to the development and formulation of the research question, development of the analysis, the interpretation of data, and reviewed and edited the manuscript. M.A. is the guarantor of this work and, as such, takes responsibility for the integrity of the data and the accuracy of the data analysis.

Competing interests. The authors have no conflicts of interest to disclose.

Funding. This project has received funding from the European Union's Horizon 2020 research and innovation programme under the Marie Skłodowska-Curie grant agreement No 754425. The funding source had no involvement in the study.

Data sharing statement. Data are available upon reasonable request.

For peer review only

REFERENCES

(1) Kwaliteitsinstituut voor de Gezondheidszorg, CBO. Multidisciplinaire richtlijn: Cardiovaculair risicomanagement 2006 [Multidisciplinary guidelines: Cardiovasular risk management 2006]. 2006.

(2) King P, Peacock I, Donnelly R. The UK Prospective Diabetes Study (UKPDS): clinical and therapeutic implications for type 2 diabetes. Br J Clin Pharmacol 1999;48(5):643-648.

(3) American Diabetes Association. Standards of medical care in diabetes 2011. Diabetes Care 2011;34 Suppl 1:11.

(4) Aronow WS, Fleg JL, Pepine CJ, et al. ACCF/AHA 2011 expert consensus document on hypertension in the elderly: a report of the American College of Cardiology Foundation Task Force on Clinical Expert Consensus Documents. Circulation 2011;123(21):2434-2506.

(5) Mancia G, Laurent S, Agabiti-Rosei E, et al. Reappraisal of European guidelines on hypertension management: a European Society of Hypertension Task Force document. J Hypertens 2009;27(11):2121-2158.

(6) Mancia G, Fagard R, Narkiewicz K, et al. 2013 ESH/ESC guidelines for the management of arterial hypertension: the Task Force for the management of arterial hypertension of the European Society of Hypertension (ESH) and of the European Society of Cardiology (ESC). J hypertens 2013;31(7):1281-1357.

(7) Verenso. Multidisciplinaire richtlijn diabetes. Verantwoorde diabeteszorg bij kwetsbare ouderen thuis en in verzorgings of Verpleeghuizen. Deel 1. [Multidisciplinary guideline diabetes. Responsible diabetes care in vulnerable elderly at home and in residential care or nursing homes. Part 1]. Utrecht, the Netherlands: Verenso; 2011.

(8) Warnes CA, Williams RG, Bashore TM, et al. ACC/AHA 2008 guidelines for the management of adults with congenital heart disease: a report of the American College of Cardiology/American Heart Association task force on practice guidelines (writing committee to develop guidelines on the management of adults with congenital heart disease). Circulation 2008;118(23):e714-e833.

(9) American Diabetes Association. Standards of medical care in diabetes. Diabetes Care 2013;36(1):S11.

(10) Mancia G, De Backer G, Dominiczak A, et al. 2007 guidelines for the management of arterial hypertension: the task force for the management of arterial hypertension of the European Society of Hypertension (ESH) and of the European Society of Cardiology (ESC). J Hypertens 2007;25(6):1105-1187.

(11) Wiersma T, Smulders YM, Stehouwer CDA, et al. Samenvatting van de multidisciplinaire richtlijn 'Cardiovasculair risicomanagement' (herziening 2011) [Recap of the multidisciplinary guidelines Cardiovascular risk management; review 2011] . Nederlands Huisartsen Genootschap 2012;156.

For peer review only

(12) van Middelaar T, Ivens SD, van Peet PG, et al. Prescribing and deprescribing antihypertensive medication in older people by Dutch general practitioners: a qualitative study. BMJ Open 2018;8(4):e020871.

(13) Andersen UO. Trends in population blood pressure and determinant factors for population blood pressure. Danish medical journal 2017;64(3):B5353.

(14) Nilsson PM, Cederholm J, Zethelius B, et al. Trends in blood pressure control in patients with type 2 diabetes - Data from the Swedish National Diabetes Register (NDR). Blood Press 2011;20(6):348-354.

(15) Sarganas G, Knopf H, Grams D, et al. Trends in antihypertensive medication use and blood bressure control among adults with hypertension in Germany. Am J Hypertens 2016;29(1):104-113.

(16) van Hateren KJ, Drion I, Kleefstra N, et al. A prospective observational study of quality of diabetes care in a shared care setting: trends and age differences (ZODIAC-19). BMJ Open 2012;2(4):10.1136/bmjopen-001387. Print 2012.

(17) Cho YY, Sidorenkov G, Denig P. Role of patient and practice characteristics in variance of treatment quality in type 2 diabetes between general practices. PLoS ONE 2016;11(11):e0166012.

(18) Tran AT, Bakke Å, Berg TJ, et al. Are general practitioners characteristics associated with the quality of type 2 diabetes care in general practice? Results from the Norwegian ROSA4 study from 2014. Scand J Prim Health Care 2018;36(2):170-179.

(19) Cahn A, Raz I, Kleinman Y, et al. Clinical assessment of individualized glycemic goals in patients with type 2 diabetes: formulation of an algorithm based on a survey among leading worldwide diabetologists. Diabetes care 2015;38(12):2293-2300.

(20) Perk J, De Backer G, Gohlke H, et al. European Guidelines on Cardiovascular Disease Prevention in Clinical Practice (Version 2012). Int J Behav Med 2012;19(4):403-488.

(21) Drubbel I, Bleijenberg N, Kranenburg G, et al. Identifying frailty: do the Frailty Index and Groningen Frailty Indicator cover different clinical perspectives? a cross-sectional study. BMC Fam Pract 2013;14(1):64.

(22) Medlon Medischne Diagnostiek. Geschatte GFR: van MDRD naar CKD-EPI [Estimated GFR: from MDRD to CKD-EPI]. 2019; Available at: https://www.medlon.nl/professional/MDRD-naar-CKD-EPI/.

(23) White IR, Royston P, Wood AM. Multiple imputation using chained equations: Issues and guidance for practice. Stat Med 2011;30(4):377-399.

(24) Nguyen DC, John BC, Lee JK. Model checking in multiple imputation: an overview and case study. Emerg Themes Epidemiol 2017;14(1):8-12.

For peer review only - http://bmjopen.bmj.com/site/about/guidelines.xhtml

(25) Kerr EA, Zikmund-Fisher BJ, Klamerus ML, et al. The role of clinical uncertainty in treatment decisions for diabetic patients with uncontrolled blood pressure. Ann Intern Med 2008;148(10):717.

(26) Struijs JN, van Til JT, Baan CA. Experimenteren met de keten-dbc diabetes: de eerste zichtbare effecten [Experimenting with a bundled payment system for diabetes care in the Netherlands: The first tangible effects]. Bilthoven, the Netherlands: National Institute for Public Health and the Environment (RIVM); 2009.

(27) de Vries ST, Voorham J, Haaijer-Ruskamp FM, et al. Potential overtreatment and undertreatment of diabetes in different patient age groups in primary care after the introduction of performance measures. Diabetes Care 2014;37(5):1312-1320.

(28) O'Connor PJ, Bodkin NL, Fradkin J, et al. Diabetes performance measures: current status and future directions. Diabetes care 2011;34(7):1651-1659.

(29) Houweling ST, Kleefstra N, Holleman F, et al Protocollaire diabeteszorg 2013/2014, mogelijkheden voor taakdelegatie [Protocol for diabetes care 2013/2014, opportunities for task delegation]. 5th ed.: Langerhans; 2013.

(30) Hyman DJ, Pavlik VN. Self-reported hypertension treatment practices among primary care physicians: blood pressure thresholds, drug choices, and the role of guidelines and evidence-based medicine. Arch Intern Med 2000;160(15):2281-2286.

(31) Mehta SS, Wilcox CS, Schulman KA. Treatment of hypertension in patients with comorbidities: Results from the study of hypertensive prescribing practices (SHyPP). Am J Hypertens 1999;12(4):333-340.

(32) Virani SS, Pokharel Y, Steinberg L, et al. Provider understanding of the 2013 ACC/AHA cholesterol guideline. J Clin Lipidol 2015;10(3):497-504.e4.

(33) Dorans KS, Mills KT, Liu Y, et al. Trends in prevalence and control of hypertension according to the 2017 American College of Cardiology/American Heart Association (ACC/AHA) guideline. J Am Heart Assoc 2018;7(11):n/a.

(34) Lee L, Patel T, Hillier LM, et al. Identifying frailty in primary care: A systematic review. Geriatr Gerontol Int 2017;17(10):1358-1377.

For peer review only - http://bmjopen.bmj.com/site/about/guidelines.xhtml

FIGURE LEGENDS

Figure 1: American, European, and Dutch guideline recommended systolic blood pressure (BP) values for initiation of antihypertensive treatment in type 2 diabetes patients over the years (1 = American College of Cardiology/American Heart Association (ACC/AHA) guidelines (8); 2 = ACC/AHA Expert Consensus Document on Hypertension in the Elderly (4); 3 = American Diabetes Association (ADA) Standards of Medical care in Diabetes (3,9); 4 = European Society of Cardiology/Euro Heart Care (ESC/EHC) guidelines for hypertension management (5,6,10); 5 = Dutch college of General practitioners (NHG) cardiovascular risk management guidelines (1,11); 6 = Verenso multidisciplinary guidelines for the management of diabetes (7))

Figure 2: Mean last systolic blood pressure (BP) value with 95% CIs before/at antihypertensive treatment initiation (A) through the years; (B) through the years in different age groups; (C) through the years in different frailty groups. eFI = electronic frailty index

For peer review only

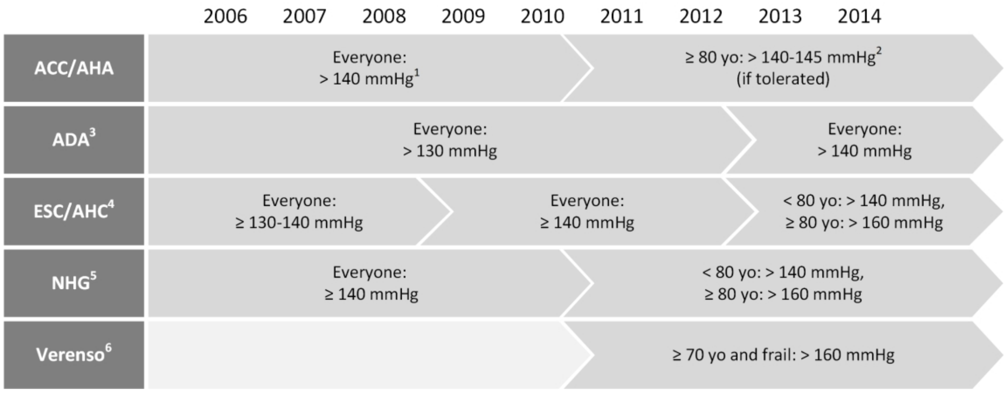

American, European, and Dutch guideline recommended systolic blood pressure (BP) values for initiation of antihypertensive treatment in type 2 diabetes patients over the years (1 = American College of Cardiology/American Heart Association (ACC/AHA) guidelines (8); 2 = ACC/AHA Expert Consensus Document on Hypertension in the Elderly (4); 3 = American Diabetes Association (ADA) Standards of Medical care in Diabetes (3,9); 4 = European Society of Cardiology/Euro Heart Care (ESC/EHC) guidelines for hypertension management (5,6,10); 5 = Dutch college of General practitioners (NHG) cardiovascular risk management guidelines (1,11); 6 = Verenso multidisciplinary guidelines for the management of diabetes (7))

192x75mm (300 x 300 DPI)

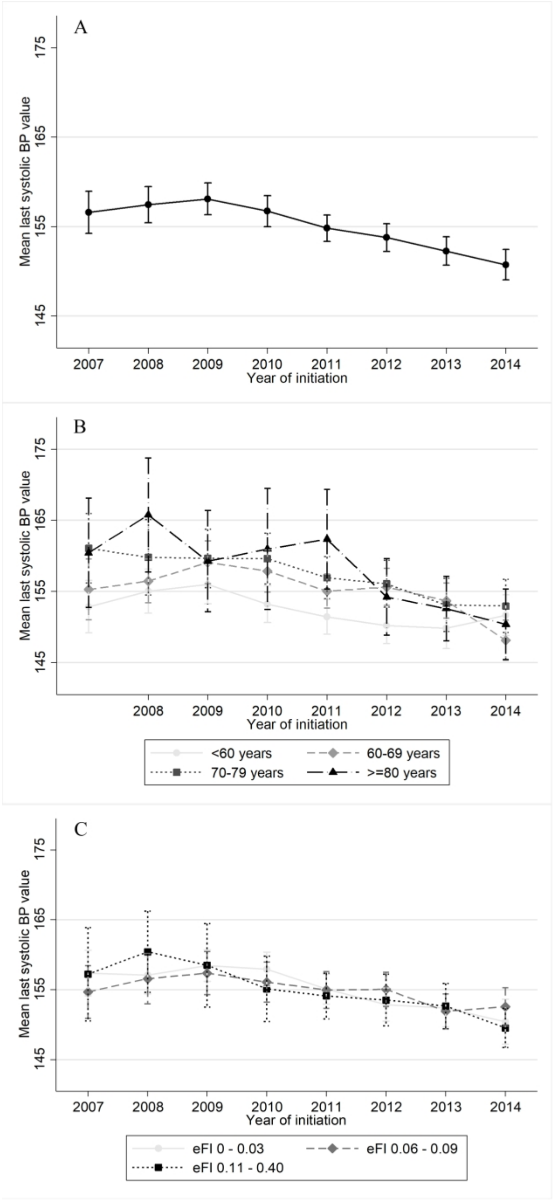

Mean last systolic blood pressure (BP) value with 95% CIs before/at antihypertensive treatment initiation (A) through the years; (B) through the years in different age groups; (C) through the years in different frailty groups. eFI = electronic frailty index

Supplementary material 1

Exclusion of patients in each calendar year

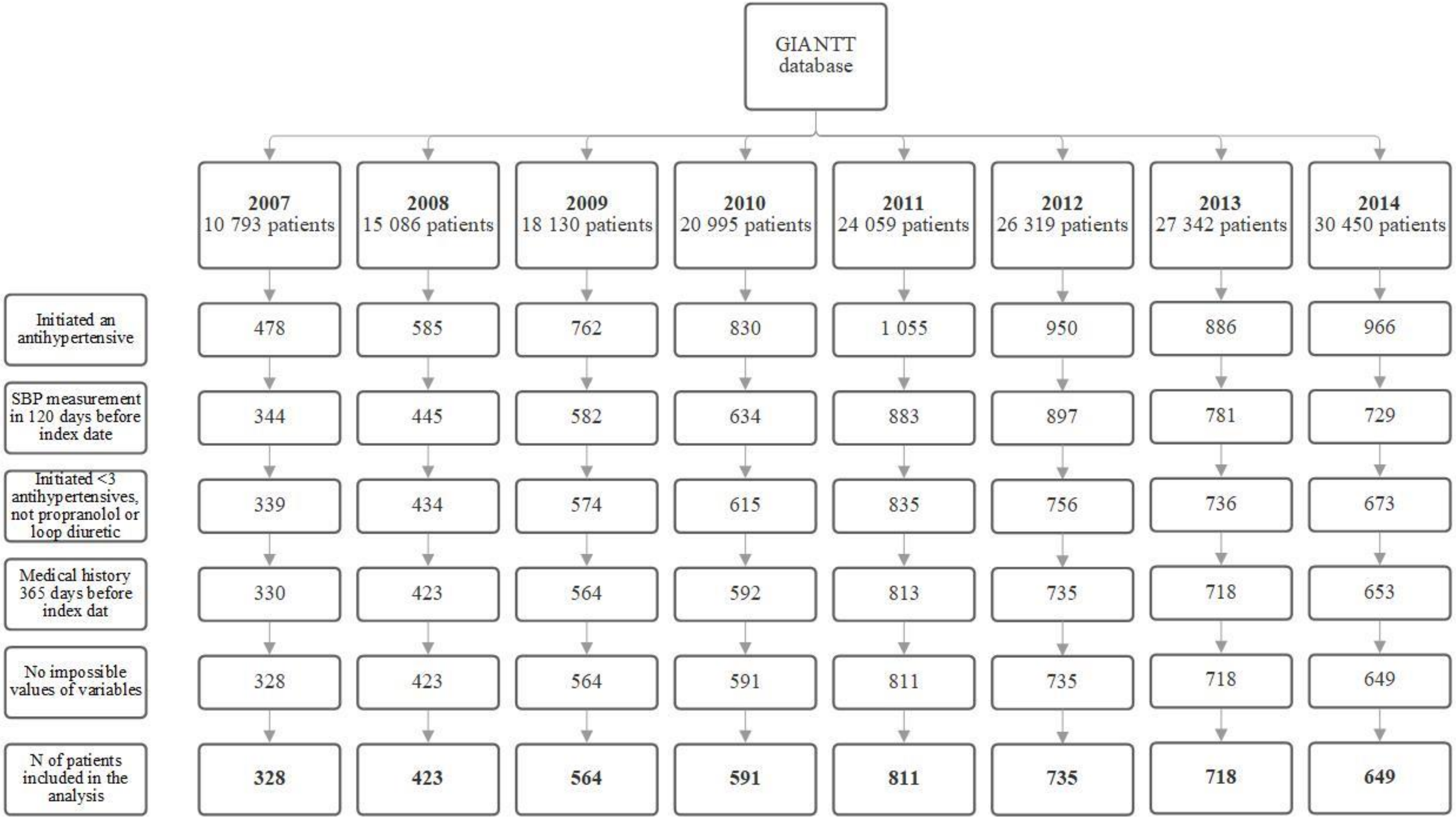

Supplementary Figure 1: Numbers of individuals available at each stage based on the inclusion and exclusion criteria per calendar year

Supplementary Table 1: Characteristics of included patients over the years

	2007	2008	2009	2010	2011	2012	2013	2014
**Number of patients**	328	423	564	591	811	735	718	649
**Females; N (%)**	154 (47)	178 (42)	270 (48)	277 (47)	360 (44)	355 (48)	355 (49)	310 (48)
**Age in years; N (%)**								
< 60	98 (30)	161 (38)	203 (36)	226 (38)	275 (34)	238 (32)	212 (30)	207 (32)
60 – 69	111 (34)	139 (33)	199 (35)	189 (32)	282 (35)	237 (32)	241 (34)	187 (29)
70 - 79	84 (26)	84 (20)	106 (19)	121 (20)	191 (24)	164 (22)	163 (23)	155 (24)
≥ 80	35 (11)	39 (9)	56 (10)	55 (9)	63 (8)	96 (13)	102 (14)	100 (15)
**Frailty in electronic Frailty Index score; N (%)**								
Less frail: 0 – 0.03	193 (59)	233 (55)	303 (54)	275 (47)	372 (46)	306 (42)	231 (32)	157 (24)
Medium frail: 0.06 – 0.09	92 (28)	124 (29)	180 (32)	204 (35)	270 (33)	267 (36)	273 (38)	218 (34)
More frail: 0.11 – 0.40	43 (13)	66 (16)	81 (14)	112 (19)	169 (21)	162 (22)	214 (30)	274 (42)
**Systolic BP at initiation in mmHg; mean ± SD**	157 ± 22	157 ± 21	158 ± 21	157 ± 22	155 ± 21	154 ± 21	152 ± 22	151 ± 22
**Diastolic BP at initiation in mmHg; mean ± SD**	86 ± 12	87 ± 11	87 ± 12	87 ± 11	86 ± 12	85 ± 12	84 ± 12	84 ± 13
**Diabetes duration < 2 years; N (%)**	96 (29)	137 (32)	139 (25)	168 (28)	236 (29)	198 (27)	154 (21)	131 (20)
**HbA1c < 7%; N (%)**	150 (59)	232 (61)	308 (59)	323 (60)	497 (64)	454 (65)	418 (60)	335 (55)
**BMI in kg/m^2; N (%)**								
< 24.9	49 (19)	67 (19)	66 (14)	79 (15)	114 (15)	132 (19)	126 (18)	118 (19)
25 – 29.9	100 (39)	142 (39)	218 (46)	222 (42)	312 (40)	280 (40)	283 (41)	231 (38)
> 30	108 (42)	151 (42)	191 (40)	229 (43)	345 (45)	284 (41)	283 (41)	262 (43)
**Dyslipidaemia; N (%)**	92 (46)	161 (50)	232 (55)	269 (58)	418 (60)	403 (62)	366 (57)	322 (58)
**eGFR ≤ 60 ml/min/1.73m2; N (%)**	37 (17)	45 (14)	69 (15)	44 (9)	70 (10)	93 (14)	100 (15)	84 (14)
**Albuminuria; N (%)**	5 (4)	9 (4)	12 (4)	10 (3)	15 (4)	16 (4)	18 (5)	16 (4)
**History of cardiovascular disease; N (%)**								
Myocardial disease	11 (3)	21 (5)	16 (3)	21 (4)	31 (4)	50 (7)	55 (8)	58 (9)
Heart failure	5 (2)	4 (1)	6 (1)	6 (1)	20 (2)	12 (2)	15 (2)	22 (3)
Stroke	13 (4)	11 (3)	19 (3)	20 (3)	25 (3)	34 (5)	45 (6)	45 (7)
**N of chronic medication at initiation; mean ± SD**	3.8 ± 2.3	3.3 ± 2.1	3.4 ± 2.4	3.5 ± 2.2	3.4 ± 2.3	3.7 ± 2.6	3.6 ± 2.5	4.0 ± 2.8

**Glucose lowering medication at initiation; N (%)**								
No medication	77 (23)	90 (21)	149 (26)	137 (23)	200 (25)	228 (31)	217 (30)	171 (26)
1 oral	129 (39)	179 (42)	199 (35)	247 (42)	363 (45)	271 (37)	283 (39)	266 (41)
2 oral	71 (22)	103 (24)	144 (26)	139 (24)	161 (20)	143 (19)	121 (17)	100 (15)
3 oral or more and/or insulin	51 (16)	51 (12)	72 (13)	68 (12)	87 (11)	93 (13)	97 (14)	112 (17)
**Treated with a lipid lowering drug; N (%)**	219 (67)	255 (60)	298 (53)	343 (58)	447 (55)	417 (57)	408 (57)	362 (56)
**Initiated drug class; N (%)**								
Renin-angiotensin-aldosterone system inhibitor	185 (60)	241 (61)	330 (63)	319 (60)	453 (62)	415 (63)	379 (60)	323 (56)
Diuretic	59 (19)	73 (18)	101 (19)	104 (20)	123 (17)	108 (16)	89 (14)	105 (18)
Beta blocker	51 (17)	59 (15)	75 (14)	80 (15)	117 (16)	93 (14)	116 (18)	107 (19)
Calcium channel blocker	14 (5)	22 (6)	21 (4)	29 (5)	32 (4)	40 (6)	44 (7)	38 (7)
Combination of antihypertensives	19 (6)	28 (7)	37 (7)	59 (10)	86 (11)	79 (11)	90 (13)	76 (12)

For peer review only

Supplementary material 2

Sensitivity analysis using mean of last two blood pressure measurements (N =2 947)

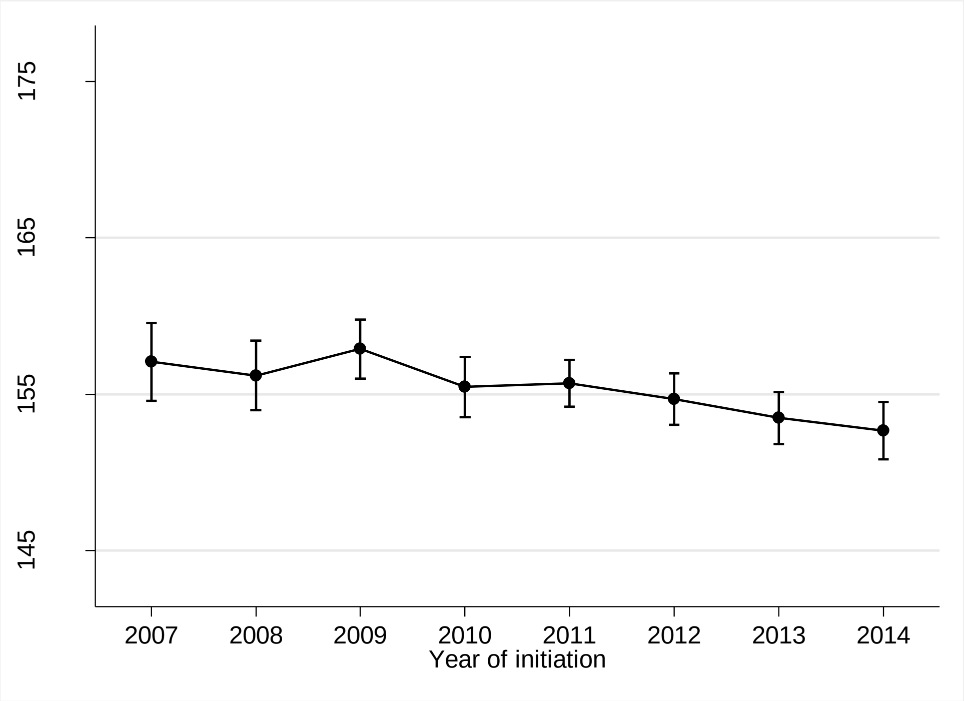

(A)

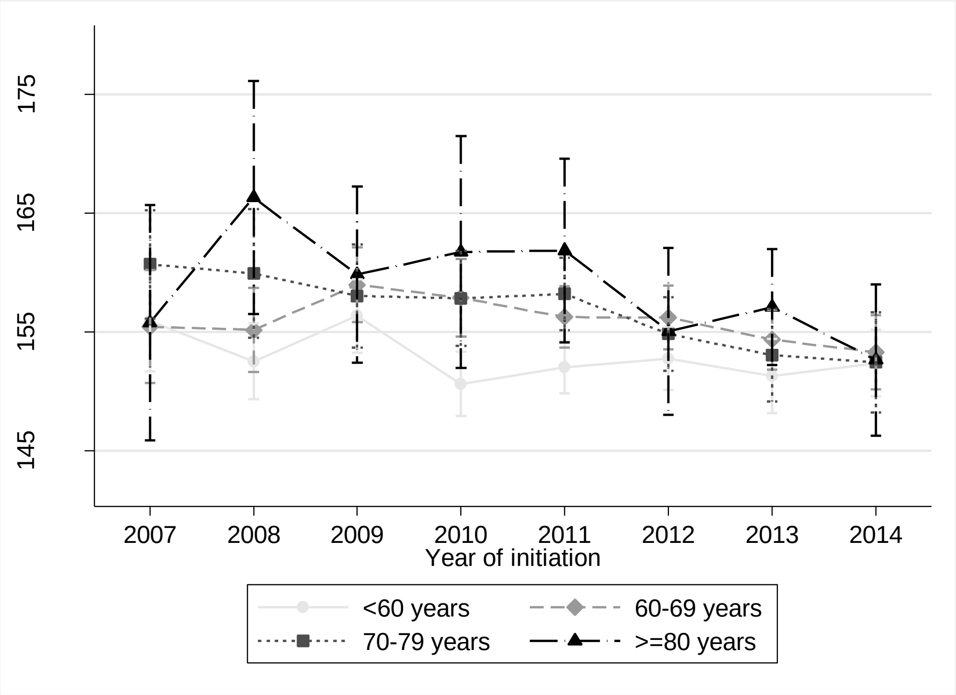

(B)

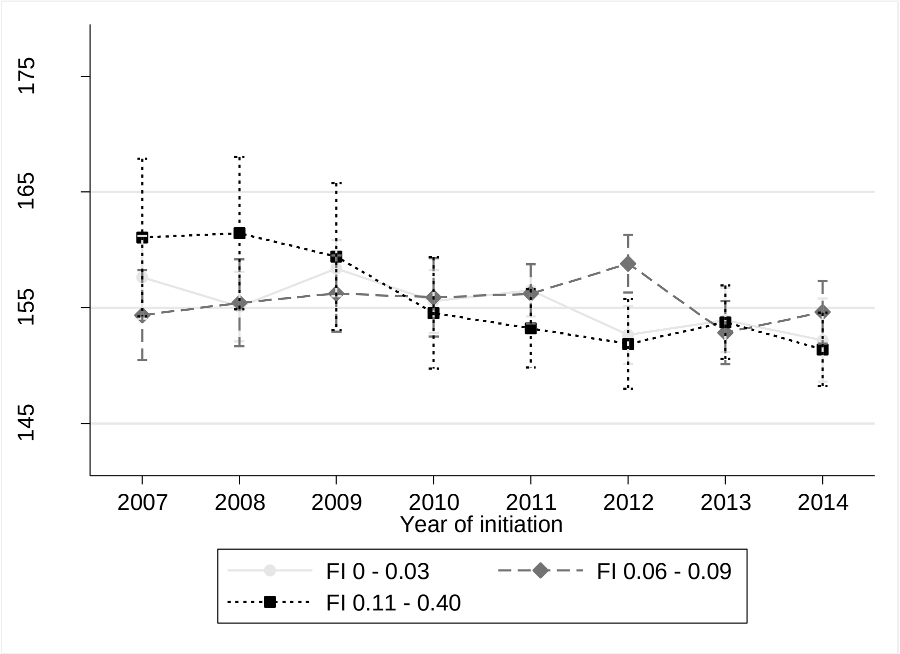

(C)

Supplementary Figure 2: Mean of last two systolic blood pressure (BP) levels with 95% CIs before/at antihypertensive treatment initiation (A) through the years; (B) through the years in different age groups and (C) through the years in different frailty groups.

Supplementary Table 2: Influence of calendar year and age or frailty on blood pressure thresholds (multilevel analysis) using the mean of last two systolic blood pressure measurements

	β	95% CI	P	
**AGE***				
Calendar year	-0.894	-2.307, 0.519	0.215	<0.001‡
Calendar year^2	-0.001	-0.146, 0.148	0.993	
Age <60 years	-8.367	-10.910, -5.824	<0.001	
Age 60 – 69 years	-4.271	-6.714, -1.828	0.001	
Age 70 – 79 years	-2.921	-5.349, -0.492	0.018	
Age ≥80 years		reference group		
Interaction year*age		none are significant		
**FRAILTY†**				
Calendar year	-0.867	-2.290, 0.556	0.232	<0.001‡
Calendar year^2	-0.013	-0.161, 0.136	0.866	
Frailty 0 – 0.03	-3.501	-5.390, -1.611	0.000	
Frailty 0.06 – 0.09	-1.455	-3.231, 0.322	0.109	
Frailty 0.11 – 0.40		reference group		
Interaction year*frailty		none are significant		

The intraclass correlation coefficient (ICC) calculated from the empty model was 0.050.

* the age model was adjusted for sex, duration of diabetes, number of chronic medication at initiation, number and/or type of glucose lowering therapy, lipid lowering therapy, presence of albuminuria, presence of dyslipidaemia, haemoglobin A1C, history of cardiovascular events, estimated glomerular filtration rate and BMI

† the frailty model was adjusted for sex, duration of diabetes, number and/or type of glucose lowering therapy, lipid lowering therapy and haemoglobin A1C

‡ joint significance of calendar year and calendar year^2 using Wald test

Supplementary material 3

Sensitivity analysis using electronic frailty index as a continuous variable

There were no statistically significant differences in blood pressure thresholds between patients with different frailty (Supplementary table 3).

Supplementary Table 3: Influence of calendar year and frailty on blood pressure thresholds (multilevel analysis), using frailty index as a continuous variable

	β	95% CI	P	
**FRAILTY**[†]				
Calendar year	0.211	-1.135, 1.557	0.759	<0.001[‡]
Calendar year^2	-0.147	-0.288, -0.007	0.039	
Frailty	-9.324	-20.747, 2.098	0.110	
Interaction year*frailty		none are significant		

[†] the frailty model was adjusted for sex, duration of diabetes, number and/or type of glucose lowering therapy, lipid lowering therapy and haemoglobin A1C
[‡] joint significance of calendar year and calendar year^2 using Wald test

For peer review only

Supplementary material 4

Supplementary Table 4: Characteristics of included patients: comparison of complete cases and cases with missing values

	**complete cases**	**cases with missings**
Patients; N (%)	3 545 (74)	1 274 (26)
Females; N (%)	1 602 (45)	675 (52)
Age in years; mean ± SD	64 ± 12	65 ± 13
Frailty index; median (Q1 - Q3)	0.08 (0.06 – 0.11)	0.08 (0.03 – 0.11)
SBP at initiation in mmHg; mean ± SD	154 ± 21	158 ± 23
DBP at initiation in mmHg; mean ± SD	85 ± 12	87 ± 13
Diabetes duration in years; mean ± SD	5.4 ± 5.3	5.4 ± 5.5
N of chronic medication at initiation; mean ± SD	3.6 ± 2.4	3.5 ± 2.6
Treated with a lipid lowering drug; N (%)	2 134 (60)	615 (48)
Initiated with one antihypertensive; N (%)	3 215 (91)	1 130 (89)

For peer review only

Supplementary material 5

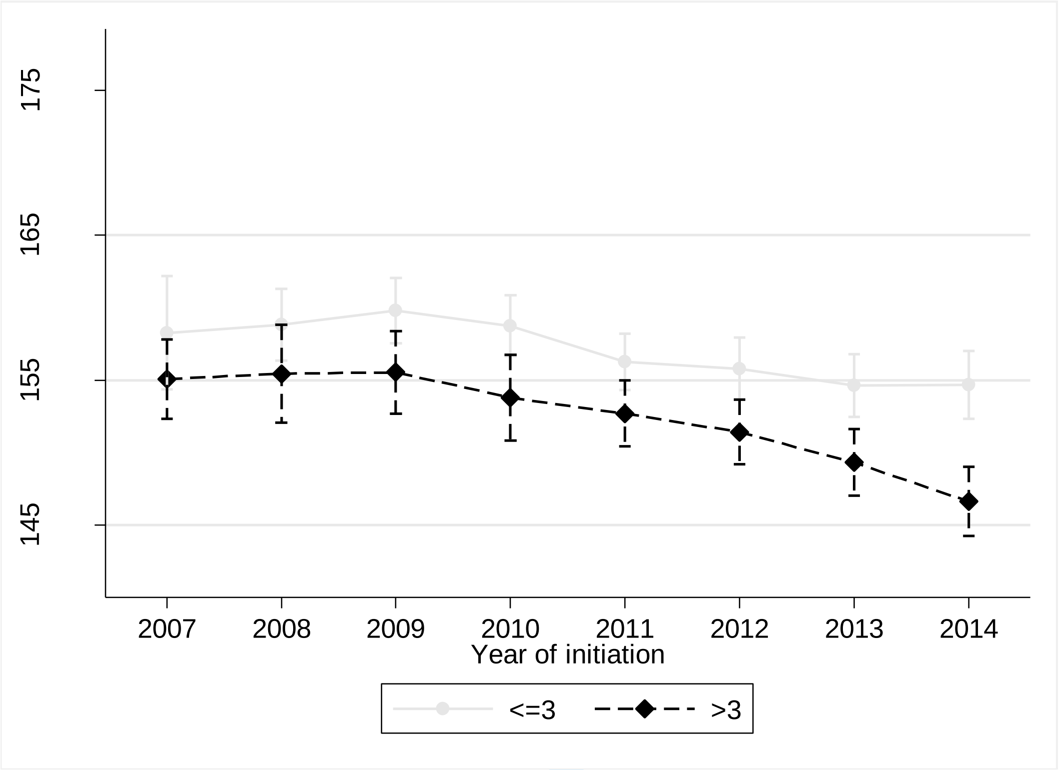

Supplementary Figure 3: Mean last systolic blood pressure value with 95% CIs before/at antihypertensive treatment initiation through the years in patients with different number of chronic medication at initiation.

Supplementary table 5: Multilevel analysis of number of chronic medication at initiation

	β	95% CI (min, max)	P	
**Calendar year**	-0.206	-1.543, 1.130	0.762	<0.001‡
**Calendar year^2**	-0.101	-0.240, 0.037	0.152	
**3 or less chronic medication**		reference group		
**More than 3 chronic medication**	-4.107	-5.428, -2.785	<0.001	
**Interaction year*N of medication**		none are significant		

The model was adjusted for sex, duration of diabetes, number and/or type of glucose lowering therapy, lipid lowering therapy, presence of albuminuria, presence of dyslipidaemia, haemoglobin A1C, estimated glomerular filtration rate and BMI.

‡ joint significance of calendar year and calendar year^2 using Wald test

review on

For peer review only

STROBE Statement—checklist of items that should be included in reports of observational studies

	Item No	Recommendation	Page
**Title and abstract**	1	(*a*) Indicate the study's design with a commonly used term in the title or the abstract	1, 2
		(*b*) Provide in the abstract an informative and balanced summary of what was done and what was found	2
**Introduction**			
Background/rationale	2	Explain the scientific background and rationale for the investigation being reported	4
Objectives	3	State specific objectives, including any prespecified hypotheses	5
**Methods**			
Study design	4	Present key elements of study design early in the paper	5
Setting	5	Describe the setting, locations, and relevant dates, including periods of recruitment, exposure, follow-up, and data collection	5, 6
Participants	6	(*a*) *Cohort study*—Give the eligibility criteria, and the sources and methods of selection of participants. Describe methods of follow-up<br>*Cross-sectional study*—Give the eligibility criteria, and the sources and methods of selection of participants	5, 6
		(*b*) *Cohort study*—For matched studies, give matching criteria and number of exposed and unexposed	n.a.
Variables	7	Clearly define all outcomes, exposures, predictors, potential confounders, and effect modifiers. Give diagnostic criteria, if applicable	6, 7
Data sources/ measurement	8*	For each variable of interest, give sources of data and details of methods of assessment (measurement). Describe comparability of assessment methods if there is more than one group	6, 7
Bias	9	Describe any efforts to address potential sources of bias	7, 8
Study size	10	Explain how the study size was arrived at	5
Quantitative variables	11	Explain how quantitative variables were handled in the analyses. If applicable, describe which groupings were chosen and why	6, 7
Statistical methods	12	(*a*) Describe all statistical methods, including those used to control for confounding	8
		(*b*) Describe any methods used to examine subgroups and interactions	8
		(*c*) Explain how missing data were addressed	7
		(*d*) *Cohort study*—If applicable, explain how loss to follow-up was addressed<br>*Cross-sectional study*—If applicable, describe analytical methods taking account of sampling strategy	n.a.
		(*e*) Describe any sensitivity analyses	8

Continued on next page

For peer review only

**Results**			
Participants	13*	(a) Report numbers of individuals at each stage of study—eg numbers potentially eligible, examined for eligibility, confirmed eligible, included in the study, completing follow-up, and analysed	Supplementary Figure 1
		(b) Give reasons for non-participation at each stage	
		(c) Consider use of a flow diagram	
Descriptive data	14*	(a) Give characteristics of study participants (eg demographic, clinical, social) and information on exposures and potential confounders	9, 10 Supplemenary Table 1
		(b) Indicate number of participants with missing data for each variable of interest	10
		(c) *Cohort study*—Summarise follow-up time (eg, average and total amount)	n.a.
Outcome data	15*	*Cohort study*—Report numbers of outcome events or summary measures over time	10, 11
		Cross-sectional study—Report numbers of outcome events or summary measures	10, 11
Main results	16	(*a*) Give unadjusted estimates and, if applicable, confounder-adjusted estimates and their precision (eg, 95% confidence interval). Make clear which confounders were adjusted for and why they were included	10, 11
		(*b*) Report category boundaries when continuous variables were categorized	9, 10
		(*c*) If relevant, consider translating estimates of relative risk into absolute risk for a meaningful time period	n.a.
Other analyses	17	Report other analyses done—eg analyses of subgroups and interactions, and sensitivity analyses	10, 11, Supplementary material
**Discussion**			
Key results	18	Summarise key results with reference to study objectives	11, 12
Limitations	19	Discuss limitations of the study, taking into account sources of potential bias or imprecision. Discuss both direction and magnitude of any potential bias	12
Interpretation	20	Give a cautious overall interpretation of results considering objectives, limitations, multiplicity of analyses, results from similar studies, and other relevant evidence	12 - 14
Generalisability	21	Discuss the generalisability (external validity) of the study results	14, 15
**Other information**			
Funding	22	Give the source of funding and the role of the funders for the present study and, if applicable, for the original study on which the present article is based	16

For peer review only

Changes in blood pressure thresholds for initiating antihypertensive medication in diabetes patients: A repeated cross-sectional study focusing on the impact of age and frailty

Journal:	*BMJ Open*
Manuscript ID	bmjopen-2020-037694.R2
Article Type:	Original research
Date Submitted by the Author:	16-Jul-2020
Complete List of Authors:	Ambrož, Martina; University Medical Centre Groningen,<br>de Vries, Sieta; University Medical Centre Groningen, Clinical Pharmacy and Pharmacology<br>Sidorenkov, Grigory; University Medical Centre Groningen, Epidemiology<br>Hoogenberg, Klaas; Martini Ziekenhuis<br>Denig, Petra; University Medical Centre Groningen, Clinical Pharmacy & Pharmacology
**Primary Subject Heading**:	Diabetes and endocrinology
Secondary Subject Heading:	Cardiovascular medicine, Epidemiology, Evidence based practice, General practice / Family practice, Pharmacology and therapeutics
Keywords:	General diabetes < DIABETES & ENDOCRINOLOGY, PRIMARY CARE, Hypertension < CARDIOLOGY, EPIDEMIOLOGY, GERIATRIC MEDICINE, Protocols & guidelines < HEALTH SERVICES ADMINISTRATION & MANAGEMENT

I, the Submitting Author has the right to grant and does grant on behalf of all authors of the Work (as defined in the below author licence), an exclusive licence and/or a non-exclusive licence for contributions from authors who are: i) UK Crown employees; ii) where BMJ has agreed a CC-BY licence shall apply, and/or iii) in accordance with the terms applicable for US Federal Government officers or employees acting as part of their official duties; on a worldwide, perpetual, irrevocable, royalty-free basis to BMJ Publishing Group Ltd ("BMJ") its licensees and where the relevant Journal is co-owned by BMJ to the co-owners of the Journal, to publish the Work in this journal and any other BMJ products and to exploit all rights, as set out in our licence.

The Submitting Author accepts and understands that any supply made under these terms is made by BMJ to the Submitting Author unless you are acting as an employee on behalf of your employer or a postgraduate student of an affiliated institution which is paying any applicable article publishing charge ("APC") for Open Access articles. Where the Submitting Author wishes to make the Work available on an Open Access basis (and intends to pay the relevant APC), the terms of reuse of such Open Access shall be governed by a Creative Commons licence – details of these licences and which Creative Commons licence will apply to this Work are set out in our licence referred to above.

Other than as permitted in any relevant BMJ Author's Self Archiving Policies, I confirm this Work has not been accepted for publication elsewhere, is not being considered for publication elsewhere and does not duplicate material already published. I confirm all authors consent to publication of this Work and authorise the granting of this licence.

For peer review only

Changes in blood pressure thresholds for initiating antihypertensive medication in diabetes patients: A repeated cross-sectional study focusing on the impact of age and frailty

Martina Ambrož, MPharm[a]; Sieta T. de Vries, PhD[a]; Grigory Sidorenkov, PhD[b]; Klaas Hoogenberg, MD, PhD[c]; Petra Denig, PhD[a]

[a] Department of Clinical Pharmacy and Pharmacology, University of Groningen, University Medical Center Groningen, Hanzeplein 1, 9713 GZ, Groningen, The Netherlands

[b] Department of Epidemiology, University of Groningen, University Medical Center Groningen, Hanzeplein 1, 9713 GZ, Groningen, The Netherlands

[c] Department of Internal Medicine, Martini Hospital, Van Swietenplein 1, 9728 NT, Groningen, the Netherlands

Declarations of interest: none

Corresponding author: Martina Ambrož, PO Box 30.001, 9700 RB Groningen, +31 613914263, m.ambroz@umcg.nl

ORCID:

Martina Ambrož https://orcid.org/0000-0003-1319-4898

Sieta T. de Vries https://orcid.org/0000-0001-6090-2434

Grigory Sidorenkov https://orcid.org/0000-0002-1926-8862

Petra Denig https://orcid.org/0000-0002-7929-4739

Word count: 2 763

For peer review only

ABSTRACT

Objective

To assess trends in systolic blood pressure (SBP) thresholds at initiation of antihypertensive treatment in type 2 diabetes (T2D) patients and the impact of age and frailty on these trends.

Study Design and Setting

A repeated cross-sectional cohort study (2007-2014) using the Groningen Initiative to Analyze Type 2 diabetes Treatment (GIANTT) database was conducted. The influence of calendar year, age or frailty and the interaction between year and age or frailty on SBP thresholds were assessed using multilevel regression analyses adjusted for potential confounders.

Results

We included 4 819 patients. The mean SBP at treatment initiation was 157mmHg in 2007, rising to 158mmHg in 2009 and decreasing to 151mmHg in 2014. This quadratic trend was significant ($p<0.001$). Older patients initiated treatment at higher SBP, but similar decreasing trends after 2009 were observed in all age groups. There were no significant differences in SBP thresholds between patients with different frailty groups. The association between year and SBP threshold was not influenced by age or frailty.

Conclusion

After an initial rise, the observed SBP thresholds decreased over time and were not influenced by age or frailty. This is in contrast with changed guideline recommendations towards more personalized treatment during the study period and illustrates that changing prescribing practice may take considerable time. Patient-specific algorithms and tools focusing on when and when not to initiate treatment could be helpful to support personalized diabetes care.

Key words: diabetes mellitus, type 2; blood pressure; primary health care; drug utilization; elderly; frail

For peer review only - http://bmjopen.bmj.com/site/about/guidelines.xhtml

Strengths and limitations:

- This is a first study investigating trends in systolic blood pressure thresholds at initiation of antihypertensive treatment in patients with type 2 diabetes.
- Real-world data from primary care of a large number of type 2 diabetes patients initiating antihypertensive treatment were used.
- The analyses were adjusted for a range of possible confounders and multiple imputation was used to reduce any possible bias that could have occurred due to missing data.
- The number of included general practices and patients fluctuated over the years.
- Frailty might have been underestimated due to incomplete cording of International Classification of Primary Care diagnoses in medical records.

For peer review only

INTRODUCTION

Treatment of hypertension in patients with type 2 diabetes (T2D) reduces cardiovascular risk, but guideline recommendations on when to initiate antihypertensive treatment to best balance the benefits and risks of treatment have changed over time. In the past, the recommended systolic blood pressure (SBP) threshold for treatment initiation ranged from 130mmHg to 140mmHg (1-7). A personalized approach, however, has been advocated in the last decade for older and/or frail patients, who are at increased risk of adverse outcomes related to low BP levels (4,6,7). Since 2011, treatment guidelines started to recommend higher SBP thresholds in these patients (Figure 1; 3-11). A recent interview study showed that Dutch general practitioners were indeed somewhat reluctant to initiate antihypertensive treatment in older and/or frail patients (12). Studies showing at which SBP thresholds physicians initiate antihypertensive treatment in older or frail patients are lacking.

A Danish study observed an average SBP level in the general population of 148mmHg before they received antihypertensive treatment in the period from 1976 to 2004 (13). Trend studies on antihypertensive medication use and hypertension control in individuals with T2D show that the percentage of people achieving the recommended SBP target of <140mmHg increased over the last twenty years (14,15). An observational study conducted in the Netherlands showed that the mean achieved SBP decreased from 155mmHg in 1998 to 140mmHg in 2008 in all age groups, with a mean SBP being lower in younger patients. No relevant differences in trends were observed between different age groups (16). This indicates that blood pressure control in patients with T2D has generally improved over time. The extent to which the more recent personalized guideline recommendations are followed, however, is not clear. This may depend on physician or practice characteristics (17,18), resulting in variability between treatment decisions (19).

For peer review only - http://bmjopen.bmj.com/site/about/guidelines.xhtml

For peer review only

The aim of this study was to assess trends in SBP thresholds for initiating antihypertensive medication in patients with T2D and the impact of changed treatment recommendations for older and frail patients. We looked at the period between 2007 and 2014, for which we hypothesized that SBP thresholds would remain similar among young and non-frail patients but would increase among older and/or frail patients. Our secondary aim was to assess to what extent SBP thresholds for treatment initiation varied across general practices.

METHODS

Study design and population

This was a repeated cross-sectional dynamic cohort study for the years 2007 to 2014. The Groningen Initiative to Analyse Type-2 diabetes Treatment (GIANTT; www.giantt.nl) database was used, which contains anonymous electronic medical records data of patients with T2D treated in primary care in the north of the Netherlands.

Patients were included per calendar year when they had a diagnosis of T2D and were ≥18 years. We excluded patients who were not included in the database for at least 365 days before antihypertensive treatment initiation and did not initiate treatment with an antihypertensive (Anatomical Therapeutic Chemical (ATC) codes C03, C04, C07, C08, C09) in that year. Antihypertensive treatment initiation was defined as an initial prescription without a prescription of any antihypertensive drug in the preceding 365 days. Furthermore, patients were excluded when they did not have a documented SBP level within 120 days before or at the day of treatment initiation or when they initiated treatment with three or more drug classes, since it is unlikely that this was a true initiation. It is assumed that these are patients with prevalent antihypertensive treatment, who entered the dynamic cohort during the study period. Moreover, we excluded patients initiating propranolol or a loop diuretic (furosemide, bumetanide), since these are commonly prescribed for other indications (i.e

For peer review only

migraine prophylaxis or short-term use in patients with edema, respectively). No approval from an ethics committee is needed for studies using data from anonymous medical records in the Netherlands. An exemption letter from University Medical Center Groningen Medical Ethics Review Board was obtained (reference number M19.235285).

Outcome variable

The outcome was the patient's most recent office SBP level in the 120 days before or on the day of antihypertensive treatment initiation.

Explanatory variables

The following explanatory variables were included: calendar year, age or frailty of the patient and the interaction between year and age or frailty. Age was calculated on January 1 of each year and was categorized in four groups (<60 years, 60–69 years, 70–79 years and ≥80 years) related to cut-off values mentioned in several guidelines (7,11,20). Frailty was calculated using a previously developed electronic frailty index (eFI), which is based on 140 International Classification of Primary Care (ICPC) coded symptoms and diagnoses from the medical history as well as the existence of polypharmacy (21). These ICPC codes are grouped into 36 deficits, for which patients get points. For chronic conditions, a diagnostic code anytime in the past is included, whereas for short-term or episodic conditions only diagnostic codes from the past year are included. The sum of the points from the deficits divided by 36 is the indication of frailty and can take a value between 0 (patient has no deficits) and 1 (patient has all possible deficits). Since all included patients had diabetes, we excluded diabetes from the eFI, thus focusing on additional frailty. There are no validated clinical cut-off values for the eFI, therefore, we categorized the scores in tertiles based on the eFI values in our study population to compare less-, medium- and more frail patients.

Confounders

For peer review only - http://bmjopen.bmj.com/site/about/guidelines.xhtml

The following patient characteristics were included as possible confounders: sex, diabetes duration (<2 years or ≥2 years), presence of dyslipidemia (LDL ≥2.5 mmol/L), glycated hemoglobin level (HbA_{1c} <7% or ≥7%), estimated glomerular filtration rate (eGFR; ≤60 mL/min or >60 mL/min), presence of elevated albuminuria (albumin creatinine ratio ≥30 mg/g or albumin in 24h urine ≥300 mg), history of cardiovascular events (presence yes/no of myocardial disease, heart failure or stroke), body mass index (BMI; <24.9 kg/m^2, 25–29.9 kg/m^2 or ≥30 kg/m^2), number of prescribed chronic medication (continuous variable), number and type of glucose lowering treatment (none, one oral, two oral, three or more oral and/or insulin), and lipid lowering treatment (none or one/more drug classes). The most recent laboratory values available in the 365 days before or seven days after treatment initiation were used. BMI was calculated from weight and height or extracted from the database in case these were not available. The eGFR was calculated from serum creatinine using the Modification of Diet in Renal Disease-4 equation for the years 2007 to 2009, and using the Chronic Kidney Disease Epidemiology Collaboration equation from 2010 onwards, since the standard way of calculating eGFR in the Netherlands changed during the study period (22). In case serum creatinine was not available, the eGFR was extracted from the database when available. Prescribed medication was assessed in the 120 days before or at the day of treatment initiation.

Missing data

There were no missing data for the explanatory variables. Values of confounders with <20% of missing values were imputed using multiple imputation by chained equation (23). For patients without albuminuria measurements (47%), we assumed that they did not have elevated albuminuria, since urine samples were less likely to be collected in our study period for patients without suspected renal function problems.

Analyses

For peer review only - http://bmjopen.bmj.com/site/about/guidelines.xhtml

For peer review only

Descriptive statistics were performed to examine patient characteristics per calendar year.

We conducted multilevel regression analyses with a two-level random intercept model to account for patients being nested within general practices. First, using the empty model, which includes only the outcome variable, we calculated the intraclass correlation coefficient to assess the variance that is attributed to general practices. Second, we added the confounders to assess the overall trend over the years. We compared a linear and a quadratic model using the Wald test to choose the best fitting final model. In the final model we assessed the effect of age or frailty on the trends by adding the explanatory variables and the interaction between year and age or frailty on SBP levels at treatment initiation.

Additional subgroup analyses were conducted for each age and frailty group to assess changes over time in these subpopulations using the final model. After applying Bonferroni correction for multiple testing, significance levels were set at $p<0.0125$ (per age group) and $p<0.0167$ (per frailty group).

Furthermore, we conducted sensitivity analyses using the average of the last two SBP levels instead of a single SBP measurement and using eFI as a continuous variable in the final model.

The analyses were conducted in Stata version 14 (Stata Corp., College Station, TX).

Patient and public involvement

Patients and public were not involved in this study.

RESULTS

A total of 4 819 patients initiating antihypertensive treatment in the period 2007 to 2014 were included (Table 1). A flow chart of excluded patients per calendar year is presented in Supplementary Figure 1. Patient characteristics were generally similar throughout the years (Supplementary Table1). Seventy-four percent of included patients had no missing values.

For peer review only - http://bmjopen.bmj.com/site/about/guidelines.xhtml

Table 1: Characteristics of included patients (N = 4 819)

**Calendar year**		**Included patients**
	2007	328
	2008	423
	2009	564
	2010	591
	2011	811
	2012	735
	2013	718
	2014	649
**Females; N (%)**		2 259 (47)
**Age in years; N (%)**		
	< 60	1620 (34)
	60 – 69	1 585 (33)
	70 – 79	1 068 (22)
	≥ 80	546 (11)
**Frailty in electronic Frailty Index score; N (%)**		
	Less frail: 0 – 0.03	2 070 (43)
	Medium frail: 0.06 – 0.09	1 628 (34)
	More frail: 0.11 – 0.40	1 121 (23)
**Systolic BP at initiation in mmHg; mean ± SD**		155 ± 22
**Diastolic BP at initiation in mmHg; mean ± SD**		85 ± 12
**Diabetes duration <2 years; N (%)**		1 259 (26)
**HbA_{1c} < 7%; N (%)** †		2 717 (61)
**BMI in kg/m^2; N (%)** ‡		
	< 24.9	751 (17)
	25 – 29.9	1 788 (41)
	≥ 30	1 853 (42)
**Dyslipidaemia; N (%)** §		2 263 (57)
**eGFR ≤ 60 ml/min/1.73m2; N (%)** ¶		542 (13)
**Elevated albuminuria (%)** ‖		101 (4)
**History of cardiovascular disease; N (%)**		
	Myocardial disease[1]	263 (5)
	Heart failure[2]	90 (2)
	Stroke[3]	212 (4)
**Number of chronic medication at initiation; mean ± SD**		3.6 ± 2.5
**Glucose lowering medication at initiation; N (%)**		
	No medication	1 269 (26)
	1 oral	1 937 (40)
	2 oral	982 (21)
	3 oral or more and/or insulin	631 (13)
**Treated with a lipid lowering drug; N (%)**		2 749 (57)
**Initiated drug class; N (%)**		
	Renin-angiotensin-aldosterone system inhibitor	2 645 (55)
	Diuretic	762 (16)
	Beta blocker	689 (14)
	Calcium channel blocker	240 (5)
	Combination of antihypertensives	474 (10)

For peer review only - http://bmjopen.bmj.com/site/about/guidelines.xhtml

†hemoglobin A1C (HbA_{1c}): 352 (7.3 %) missing values; ‡BMI: 427 (8.9 %) missing values; § LDL-cholesterol: 874 (18.1 %) missing values; ¶ estimated glomerular filtration rate (eGFR): 677 (14.0 %) missing values; || albuminuria: 2 274 (47.2%) missing values; [1] acute myocardial infarction (ICPC code K75) in the last year or other/chronic ischaemic heart disease (ICPC code K76) anytime in history; [2] heart failure (ICPC code K77) anytime in history; [3] transient cerebral ischemia (ICPC code K89) in the last year or stroke/cerebrovascular incident (ICPC code K90) anytime in history

Trends in SBP thresholds

The mean SBP level at antihypertensive treatment initiation significantly changed over time from 157mmHg (SD 22mmHg) in 2007, rising to 158mmHg (SD 21mmHg) in 2009 and thereafter decreasing to 151mmHg (SD 22mmHg) in 2014 (Figure 2A). This quadratic trend was statistically significant (p<0.001).

Age and frailty

Older patients initiated treatment at significantly higher SBP thresholds than younger patients but age did not significantly influence the relationship between calendar year and SBP threshold (Table 2, Figure 2B). In the analyses per age group, the SBP level at initiation changed significantly (quadratic model) over the years in patients aged between 60 and 69 years (p=0.001).

For peer review

Table 2: Influence of calendar year and age or frailty on blood pressure thresholds (multilevel analysis)

	**β**	**95% CI**	**P**	
**AGE**†				
Calendar year	-0.107	-1.429, 1.215	0.874	<0.001§
Calendar year^2	-0.111	-0.248, 0.027	0.114	
Age <60 years	-8.066	-10.411, -5.723	<0.001	
Age 60 – 69 years	-4.115	-6.369, -1.861	<0.001	
Age 70 – 79 years	-1.168	-3.407, 1.072	0.307	
Age ≥80 years		reference group		
Interaction year*age		none are significant		
**FRAILTY**‡				
Calendar year	0.247	-1.100, 1.593	0.719	<0.001§
Calendar year^2	-0.159	-0.299, -0.018	0.027	
Frailty 0 – 0.03	-0.060	-1.734, 1.614	0.944	
Frailty 0.06 – 0.09	0.127	-1.519, 1.772	0.880	
Frailty 0.11 – 0.40		reference group		
Interaction year*frailty		none are significant		

The intraclass correlation coefficient (ICC) calculated from the empty model was 0.032
† the age model was adjusted for sex, duration of diabetes, number of chronic medication at initiation, number and/or type of glucose lowering therapy, lipid lowering therapy, presence of albuminuria,

For peer review only - http://bmjopen.bmj.com/site/about/guidelines.xhtml

presence of dyslipidaemia, haemoglobin A1C, history of cardiovascular events, estimated glomerular filtration rate and BMI
‡ the frailty model was adjusted for sex, duration of diabetes, number and/or type of glucose lowering therapy, lipid lowering therapy and HbA_{1c}
§ joined significance of calendar year and calendar year² using Wald test

Frailty did not influence SBP thresholds for treatment initiation and it did not significantly influence the relationship between calendar year and SBP threshold (Table 2, Figure 2C). In the analyses per frailty group, the SBP level at initiation changed significantly (quadratic model) over the years in the less frail (eFI 0-0.03; p<0.001) and more frail (eFI 0.11-0.40; p=0.001) patients.

The sensitivity analyses using the mean of the last two SBP levels (Supplementary Figure 2A-C and Supplementary Table 2) and using eFI as a continuous variable (Supplementary Table 3) showed similar results.

Variation between general practices

Of the total variation in SBP level at antihypertensive treatment initiation, 3.2% could be explained by differences between general practices (Table 2, ICC=0.032).

DISCUSSION

Summary

This study shows that, after an initial rise up to 2009, SBP thresholds for antihypertensive treatment initiation decreased over time in a large cohort of patients with T2D treated in primary care. This trend occurred regardless of age and frailty, which was in contrast to our hypothesis given the changes in guideline recommendations. The variation in SBP thresholds for treatment initiation that could be attributed to general practices was small.

Strengths and limitations

The strength of our study is the large number of patients included using real-world data. Furthermore, it is a first study investigating trends in SBP thresholds at initiation of antihypertensive treatment. We focussed on the period from 2007 to 2014, expecting that the

For peer review only - http://bmjopen.bmj.com/site/about/guidelines.xhtml

shift towards more personalized diabetes care around 2011 could be observed during this period. It is possible that changes occurred in more recent years. The first limitation is the fluctuating number of general practices over the years, with different numbers of patients and practices included in each year cohort. Since little variation was explained at practice level, it is unlikely that this affected our primary findings. Second, there were some demographic differences between the patients with and without missing data (Supplementary Table 4). This bias was reduced by multiple imputation (24). Third, we could not include smoking or date of hypertension diagnosis as confounders due to amount and variability of missing values over the years. Furthermore, ICPC codes do not provide information about the severity of the comorbidities. Therefore, there may be some residual confounding which was not accounted for. Fourth, incomplete coding of ICPC diagnoses in electronic medical records may result in underestimation of frailty. Finally, SBP levels show intra-individual variability and may include higher values caused by 'white coat' hypertension (25). However, analysis of the mean last two SBP levels did not change our main findings.

Comparison with existing literature

The observed trends are not in line with changes in treatment guidelines, where higher thresholds were recommended in the older and/or frail patients, particularly in the later years. Several reasons can explain this discrepancy between our hypothesis and findings. First, Dutch healthcare practitioners may have felt pressured to initiate antihypertensive treatment at lower SBP levels in all diabetes patients after the introduction of performance indicators in 2007 (26). From 2008 onwards, they received yearly feedback on the percentage of patients achieving SBP levels of <140mmHg in their own practice as compared to other practices in the region. Although a previous study in this population did not show an increase in overtreatment after the introduction of performance indicators (27), concerns about the negative impact of such measures have been raised (28). In addition, nurse practitioners

For peer review only - http://bmjopen.bmj.com/site/about/guidelines.xhtml

increasingly became the pivot of diabetes care (26) and their educational material recommended a unified SBP target of 140mmHg at least until the end of our study period (29). It is also possible that the practitioners did not adhere to treatment guidelines either due to lack of familiarity, understanding or agreement with them (30-32). An interview study conducted in 2015 and 2016, however, suggests that Dutch general practitioners did support the idea of using a higher threshold for initiation of antihypertensive treatment in older and frail patients (12). Nevertheless, we demonstrated that guideline changes were not yet implemented more than three years after being published.

Antihypertensive treatment was initiated at relatively high SBP levels in patients of all ages but started to decline after 2009, suggesting that it took many years before the United Kingdom Prospective Diabetes Study (UKPDS) recommended threshold of 140mmHg for treatment initiation was implemented in practice (2). Other studies showed that the percentage of patients achieving an SBP <140mmHg increased in the last two decades but that this increase was smaller in older patients (15). A recent U.S. study showed that the trends in SBP levels in patients ≥60 years decreased until 2010 and remained relatively stable in the six years thereafter (33). Although this study was not restricted to patients with T2D, this seems in contrast with our findings where SBP levels after 2009 decreased in all age groups.

To our surprise, frailty did not influence the SBP threshold for treatment initiation. The frailty range in our study was rather low, which could indicate that antihypertensive treatment is initiated when the patients are still relatively fit. On the other hand, frailty can easily be overlooked due to subtle manifestations, lack of time, or a lack of consensus on the best way to assess it (34). Although the eFI was previously able to identify frailty comparable to the Groningen Frailty Index (21), it might not be in line with the practitioner’s perception of a patient’s frailty. Therefore, we conducted a post-hoc analysis using the number of chronic medication a patient was receiving at initiation as a proxy for frailty. We observed that the

For peer review only - http://bmjopen.bmj.com/site/about/guidelines.xhtml

patients being treated with more than three chronic medication (median) initiated treatment at lower SBP levels than those being treated with three or less (Supplementary Figure 3 and Supplementary Table 5). This finding suggests that lower instead of higher thresholds are used for frail patients.

Only a small part of the variation in our study could be attributed to differences between practices. This suggests that patient characteristics determine the threshold to a greater extent than practice characteristics. We only looked at variation between practices which may include decisions of two or more general practitioners within one practice. Unfortunately, we could not conduct analyses at the level of individual practitioners.

Conclusion and implications

The observed SBP thresholds at initiation of antihypertensive treatment decreased after 2009. This trend was not influenced by age or frailty, which is in contrast with changes in treatment recommendations, and may be explained by the introduction of performance indicators. Our study illustrates that changing prescribing practice may take considerable time and only publishing new recommendations might not be sufficient for their successful implementation. On the one hand, patient-specific algorithms and tools to support the timely start of antihypertensive treatment in younger patients are needed. On the other hand, also algorithms and tools to prevent the initiation of too early or strict antihypertensive treatment in older and frail patients should be developed. Furthermore, performance indicators should include the aspect of more personalized treatment recommendations. Further research is needed to assess the underlying reasons and extent of the delay in the implementation of personalized diabetes care and evaluate the impact of strategies to speed up the uptake of recommendations.

For peer review only - http://bmjopen.bmj.com/site/about/guidelines.xhtml

For peer review only

For peer review only - http://bmjopen.bmj.com/site/about/guidelines.xhtml

ACKNOWLEDGMENTS

Contributorship statement. M.A. contributed to the development and formulation of the research question, conducted the analysis, contributed to the interpretation of data, wrote the manuscript, and reviewed and edited the manuscript. S.T.d.V. contributed to the development and formulation of the research question, conducted the analysis, contributed to the interpretation of data, and reviewed and edited the manuscript. G.S. contributed to the development of the analysis, the interpretation of data, and reviewed and edited the manuscript. K.H. contributed to the development and formulation of the research question, the interpretation of data, and reviewed and edited the manuscript P.D. contributed to the development and formulation of the research question, development of the analysis, the interpretation of data, and reviewed and edited the manuscript. M.A. is the guarantor of this work and, as such, takes responsibility for the integrity of the data and the accuracy of the data analysis.

Competing interests. The authors have no conflicts of interest to disclose.

Funding. This project has received funding from the European Union's Horizon 2020 research and innovation programme under the Marie Skłodowska-Curie grant agreement No 754425. The funding source had no involvement in the study.

Data sharing statement. Data are available upon reasonable request.

For peer review only

REFERENCES

(1) Kwaliteitsinstituut voor de Gezondheidszorg, CBO. Multidisciplinaire richtlijn: Cardiovaculair risicomanagement 2006 [Multidisciplinary guidelines: Cardiovasular risk management 2006]. 2006.

(2) King P, Peacock I, Donnelly R. The UK Prospective Diabetes Study (UKPDS): clinical and therapeutic implications for type 2 diabetes. Br J Clin Pharmacol 1999;48(5):643-648.

(3) American Diabetes Association. Standards of medical care in diabetes 2011. Diabetes Care 2011;34 Suppl 1:11.

(4) Aronow WS, Fleg JL, Pepine CJ, et al. ACCF/AHA 2011 expert consensus document on hypertension in the elderly: a report of the American College of Cardiology Foundation Task Force on Clinical Expert Consensus Documents. Circulation 2011;123(21):2434-2506.

(5) Mancia G, Laurent S, Agabiti-Rosei E, et al. Reappraisal of European guidelines on hypertension management: a European Society of Hypertension Task Force document. J Hypertens 2009;27(11):2121-2158.

(6) Mancia G, Fagard R, Narkiewicz K, et al. 2013 ESH/ESC guidelines for the management of arterial hypertension: the Task Force for the management of arterial hypertension of the European Society of Hypertension (ESH) and of the European Society of Cardiology (ESC). J hypertens 2013;31(7):1281-1357.

(7) Verenso. Multidisciplinaire richtlijn diabetes. Verantwoorde diabeteszorg bij kwetsbare ouderen thuis en in verzorgings of Verpleeghuizen. Deel 1. [Multidisciplinary guideline diabetes. Responsible diabetes care in vulnerable elderly at home and in residential care or nursing homes. Part 1]. Utrecht, the Netherlands: Verenso; 2011.

(8) Warnes CA, Williams RG, Bashore TM, et al. ACC/AHA 2008 guidelines for the management of adults with congenital heart disease: a report of the American College of Cardiology/American Heart Association task force on practice guidelines (writing committee to develop guidelines on the management of adults with congenital heart disease). Circulation 2008;118(23):e714-e833.

(9) American Diabetes Association. Standards of medical care in diabetes. Diabetes Care 2013;36(1):S11.

(10) Mancia G, De Backer G, Dominiczak A, et al. 2007 guidelines for the management of arterial hypertension: the task force for the management of arterial hypertension of the European Society of Hypertension (ESH) and of the European Society of Cardiology (ESC). J Hypertens 2007;25(6):1105-1187.

(11) Wiersma T, Smulders YM, Stehouwer CDA, et al. Samenvatting van de multidisciplinaire richtlijn 'Cardiovasculair risicomanagement' (herziening 2011) [Recap of the multidisciplinary guidelines Cardiovascular risk management; review 2011] . Nederlands Huisartsen Genootschap 2012;156.

For peer review only - http://bmjopen.bmj.com/site/about/guidelines.xhtml

(12) van Middelaar T, Ivens SD, van Peet PG, et al. Prescribing and deprescribing antihypertensive medication in older people by Dutch general practitioners: a qualitative study. BMJ Open 2018;8(4):e020871.

(13) Andersen UO. Trends in population blood pressure and determinant factors for population blood pressure. Danish medical journal 2017;64(3):B5353.

(14) Nilsson PM, Cederholm J, Zethelius B, et al. Trends in blood pressure control in patients with type 2 diabetes - Data from the Swedish National Diabetes Register (NDR). Blood Press 2011;20(6):348-354.

(15) Sarganas G, Knopf H, Grams D, et al. Trends in antihypertensive medication use and blood bressure control among adults with hypertension in Germany. Am J Hypertens 2016;29(1):104-113.

(16) van Hateren KJ, Drion I, Kleefstra N, et al. A prospective observational study of quality of diabetes care in a shared care setting: trends and age differences (ZODIAC-19). BMJ Open 2012;2(4):10.1136/bmjopen-001387. Print 2012.

(17) Cho YY, Sidorenkov G, Denig P. Role of patient and practice characteristics in variance of treatment quality in type 2 diabetes between general practices. PLoS ONE 2016;11(11):e0166012.

(18) Tran AT, Bakke Å, Berg TJ, et al. Are general practitioners characteristics associated with the quality of type 2 diabetes care in general practice? Results from the Norwegian ROSA4 study from 2014. Scand J Prim Health Care 2018;36(2):170-179.

(19) Cahn A, Raz I, Kleinman Y, et al. Clinical assessment of individualized glycemic goals in patients with type 2 diabetes: formulation of an algorithm based on a survey among leading worldwide diabetologists. Diabetes care 2015;38(12):2293-2300.

(20) Perk J, De Backer G, Gohlke H, et al. European Guidelines on Cardiovascular Disease Prevention in Clinical Practice (Version 2012). Int J Behav Med 2012;19(4):403-488.

(21) Drubbel I, Bleijenberg N, Kranenburg G, et al. Identifying frailty: do the Frailty Index and Groningen Frailty Indicator cover different clinical perspectives? a cross-sectional study. BMC Fam Pract 2013;14(1):64.

(22) Medlon Medischne Diagnostiek. Geschatte GFR: van MDRD naar CKD-EPI [Estimated GFR: from MDRD to CKD-EPI]. 2019; Available at: https://www.medlon.nl/professional/MDRD-naar-CKD-EPI/.

(23) White IR, Royston P, Wood AM. Multiple imputation using chained equations: Issues and guidance for practice. Stat Med 2011;30(4):377-399.

(24) Nguyen DC, John BC, Lee JK. Model checking in multiple imputation: an overview and case study. Emerg Themes Epidemiol 2017;14(1):8-12.

For peer review only - http://bmjopen.bmj.com/site/about/guidelines.xhtml

(25) Kerr EA, Zikmund-Fisher BJ, Klamerus ML, et al. The role of clinical uncertainty in treatment decisions for diabetic patients with uncontrolled blood pressure. Ann Intern Med 2008;148(10):717.

(26) Struijs JN, van Til JT, Baan CA. Experimenteren met de keten-dbc diabetes: de eerste zichtbare effecten [Experimenting with a bundled payment system for diabetes care in the Netherlands: The first tangible effects]. Bilthoven, the Netherlands: National Institute for Public Health and the Environment (RIVM); 2009.

(27) de Vries ST, Voorham J, Haaijer-Ruskamp FM, et al. Potential overtreatment and undertreatment of diabetes in different patient age groups in primary care after the introduction of performance measures. Diabetes Care 2014;37(5):1312-1320.

(28) O'Connor PJ, Bodkin NL, Fradkin J, et al. Diabetes performance measures: current status and future directions. Diabetes care 2011;34(7):1651-1659.

(29) Houweling ST, Kleefstra N, Holleman F, et al Protocollaire diabeteszorg 2013/2014, mogelijkheden voor taakdelegatie [Protocol for diabetes care 2013/2014, opportunities for task delegation]. 5th ed.: Langerhans; 2013.

(30) Hyman DJ, Pavlik VN. Self-reported hypertension treatment practices among primary care physicians: blood pressure thresholds, drug choices, and the role of guidelines and evidence-based medicine. Arch Intern Med 2000;160(15):2281-2286.

(31) Mehta SS, Wilcox CS, Schulman KA. Treatment of hypertension in patients with comorbidities: Results from the study of hypertensive prescribing practices (SHyPP). Am J Hypertens 1999;12(4):333-340.

(32) Virani SS, Pokharel Y, Steinberg L, et al. Provider understanding of the 2013 ACC/AHA cholesterol guideline. J Clin Lipidol 2015;10(3):497-504.e4.

(33) Dorans KS, Mills KT, Liu Y, et al. Trends in prevalence and control of hypertension according to the 2017 American College of Cardiology/American Heart Association (ACC/AHA) guideline. J Am Heart Assoc 2018;7(11):n/a.

(34) Lee L, Patel T, Hillier LM, et al. Identifying frailty in primary care: A systematic review. Geriatr Gerontol Int 2017;17(10):1358-1377.

For peer review only

FIGURE LEGENDS

Figure 1: American, European, and Dutch guideline recommended systolic blood pressure (BP) values for initiation of antihypertensive treatment in type 2 diabetes patients over the years (1 = American College of Cardiology/American Heart Association (ACC/AHA) guidelines (8); 2 = ACC/AHA Expert Consensus Document on Hypertension in the Elderly (4); 3 = American Diabetes Association (ADA) Standards of Medical care in Diabetes (3,9); 4 = European Society of Cardiology/Euro Heart Care (ESC/EHC) guidelines for hypertension management (5,6,10); 5 = Dutch college of General practitioners (NHG) cardiovascular risk management guidelines (1,11); 6 = Verenso multidisciplinary guidelines for the management of diabetes (7))

Figure 2: Mean last systolic blood pressure (BP) value with 95% CIs before/at antihypertensive treatment initiation (A) through the years; (B) through the years in different age groups; (C) through the years in different frailty groups. eFI = electronic frailty index

For peer review only

For peer review only - http://bmjopen.bmj.com/site/about/guidelines.xhtml

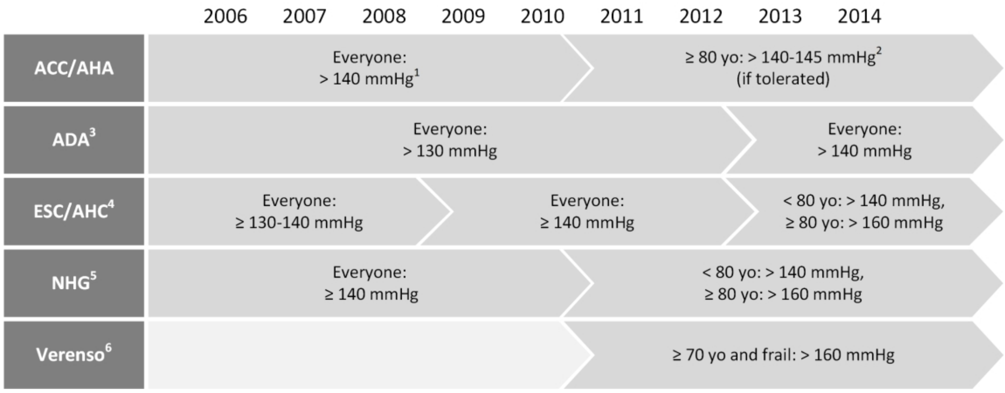

American, European, and Dutch guideline recommended systolic blood pressure (BP) values for initiation of antihypertensive treatment in type 2 diabetes patients over the years (1 = American College of Cardiology/American Heart Association (ACC/AHA) guidelines (8); 2 = ACC/AHA Expert Consensus Document on Hypertension in the Elderly (4); 3 = American Diabetes Association (ADA) Standards of Medical care in Diabetes (3,9); 4 = European Society of Cardiology/Euro Heart Care (ESC/EHC) guidelines for hypertension management (5,6,10); 5 = Dutch college of General practitioners (NHG) cardiovascular risk management guidelines (1,11); 6 = Verenso multidisciplinary guidelines for the management of diabetes (7))

192x75mm (300 x 300 DPI)

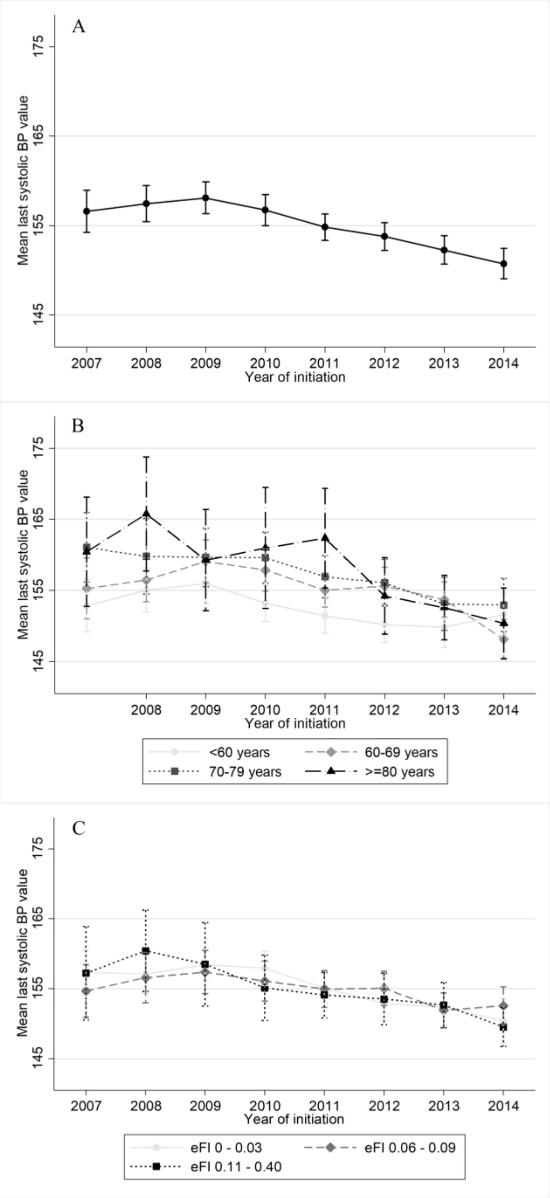

Mean last systolic blood pressure (BP) value with 95% CIs before/at antihypertensive treatment initiation (A) through the years; (B) through the years in different age groups; (C) through the years in different frailty groups. eFI = electronic frailty index

Supplementary material 1

Exclusion of patients in each calendar year

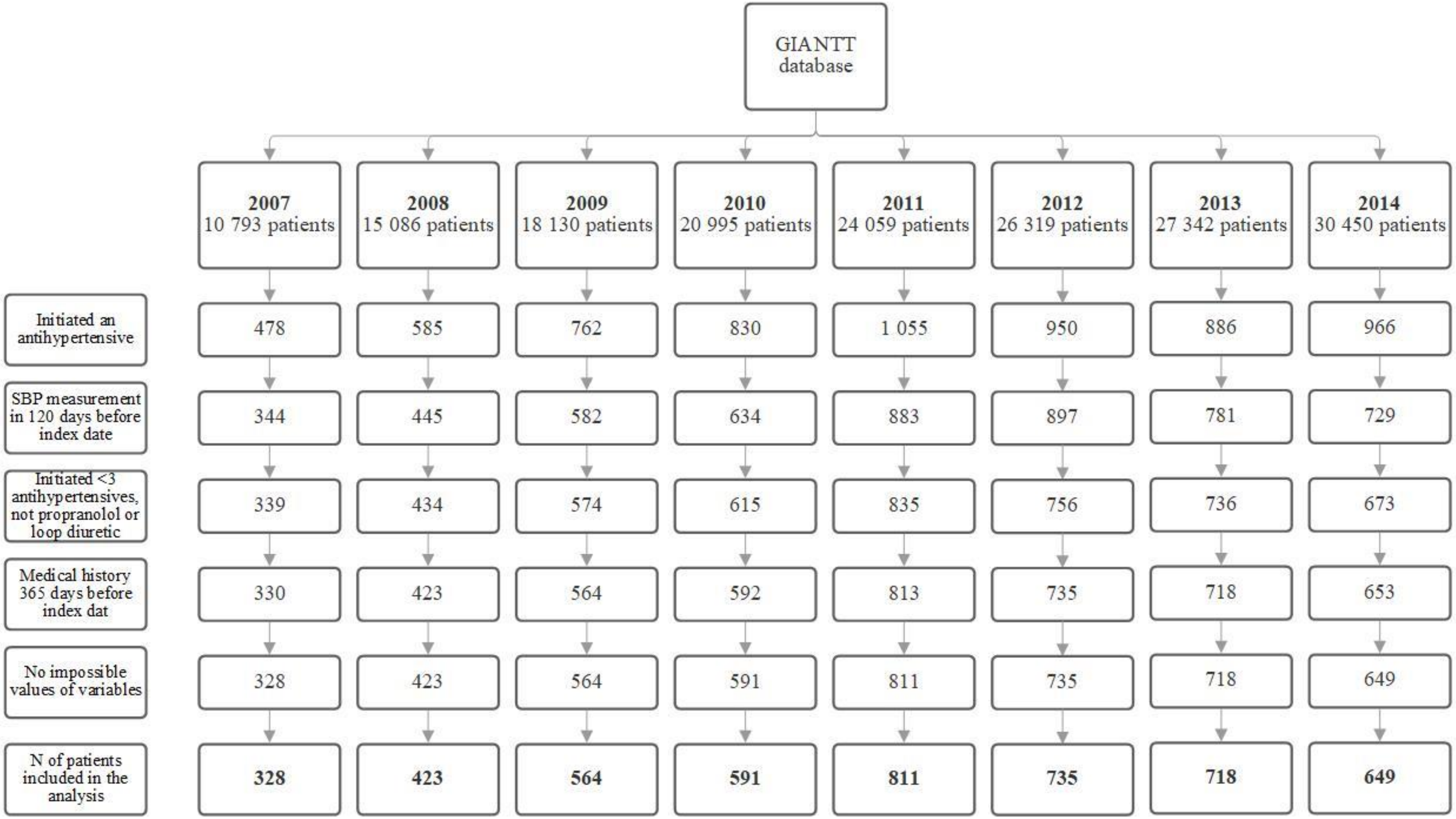

Supplementary Figure 1: Numbers of individuals available at each stage based on the inclusion and exclusion criteria per calendar year

Supplementary Table 1: Characteristics of included patients over the years

	2007	2008	2009	2010	2011	2012	2013	2014
**Number of patients**	328	423	564	591	811	735	718	649
**Females; N (%)**	154 (47)	178 (42)	270 (48)	277 (47)	360 (44)	355 (48)	355 (49)	310 (48)
**Age in years; N (%)**								
< 60	98 (30)	161 (38)	203 (36)	226 (38)	275 (34)	238 (32)	212 (30)	207 (32)
60 – 69	111 (34)	139 (33)	199 (35)	189 (32)	282 (35)	237 (32)	241 (34)	187 (29)
70 - 79	84 (26)	84 (20)	106 (19)	121 (20)	191 (24)	164 (22)	163 (23)	155 (24)
≥ 80	35 (11)	39 (9)	56 (10)	55 (9)	63 (8)	96 (13)	102 (14)	100 (15)
**Frailty in electronic Frailty Index score; N (%)**								
Less frail: 0 – 0.03	193 (59)	233 (55)	303 (54)	275 (47)	372 (46)	306 (42)	231 (32)	157 (24)
Medium frail: 0.06 – 0.09	92 (28)	124 (29)	180 (32)	204 (35)	270 (33)	267 (36)	273 (38)	218 (34)
More frail: 0.11 – 0.40	43 (13)	66 (16)	81 (14)	112 (19)	169 (21)	162 (22)	214 (30)	274 (42)
**Systolic BP at initiation in mmHg; mean ± SD**	157 ± 22	157 ± 21	158 ± 21	157 ± 22	155 ± 21	154 ± 21	152 ± 22	151 ± 22
**Diastolic BP at initiation in mmHg; mean ± SD**	86 ± 12	87 ± 11	87 ± 12	87 ± 11	86 ± 12	85 ± 12	84 ± 12	84 ± 13
**Diabetes duration < 2 years; N (%)**	96 (29)	137 (32)	139 (25)	168 (28)	236 (29)	198 (27)	154 (21)	131 (20)
**HbA1c < 7%; N (%)**	150 (59)	232 (61)	308 (59)	323 (60)	497 (64)	454 (65)	418 (60)	335 (55)
**BMI in kg/m^2; N (%)**								
< 24.9	49 (19)	67 (19)	66 (14)	79 (15)	114 (15)	132 (19)	126 (18)	118 (19)
25 – 29.9	100 (39)	142 (39)	218 (46)	222 (42)	312 (40)	280 (40)	283 (41)	231 (38)
> 30	108 (42)	151 (42)	191 (40)	229 (43)	345 (45)	284 (41)	283 (41)	262 (43)
**Dyslipidaemia; N (%)**	92 (46)	161 (50)	232 (55)	269 (58)	418 (60)	403 (62)	366 (57)	322 (58)
**eGFR ≤ 60 ml/min/1.73m2; N (%)**	37 (17)	45 (14)	69 (15)	44 (9)	70 (10)	93 (14)	100 (15)	84 (14)
**Albuminuria; N (%)**	5 (4)	9 (4)	12 (4)	10 (3)	15 (4)	16 (4)	18 (5)	16 (4)
**History of cardiovascular disease; N (%)**								
Myocardial disease	11 (3)	21 (5)	16 (3)	21 (4)	31 (4)	50 (7)	55 (8)	58 (9)
Heart failure	5 (2)	4 (1)	6 (1)	6 (1)	20 (2)	12 (2)	15 (2)	22 (3)
Stroke	13 (4)	11 (3)	19 (3)	20 (3)	25 (3)	34 (5)	45 (6)	45 (7)
**N of chronic medication at initiation; mean ± SD**	3.8 ± 2.3	3.3 ± 2.1	3.4 ± 2.4	3.5 ± 2.2	3.4 ± 2.3	3.7 ± 2.6	3.6 ± 2.5	4.0 ± 2.8

**Glucose lowering medication at initiation; N (%)**								
No medication	77 (23)	90 (21)	149 (26)	137 (23)	200 (25)	228 (31)	217 (30)	171 (26)
1 oral	129 (39)	179 (42)	199 (35)	247 (42)	363 (45)	271 (37)	283 (39)	266 (41)
2 oral	71 (22)	103 (24)	144 (26)	139 (24)	161 (20)	143 (19)	121 (17)	100 (15)
3 oral or more and/or insulin	51 (16)	51 (12)	72 (13)	68 (12)	87 (11)	93 (13)	97 (14)	112 (17)
**Treated with a lipid lowering drug; N (%)**	219 (67)	255 (60)	298 (53)	343 (58)	447 (55)	417 (57)	408 (57)	362 (56)
**Initiated drug class; N (%)**								
Renin-angiotensin-aldosterone system inhibitor	185 (60)	241 (61)	330 (63)	319 (60)	453 (62)	415 (63)	379 (60)	323 (56)
Diuretic	59 (19)	73 (18)	101 (19)	104 (20)	123 (17)	108 (16)	89 (14)	105 (18)
Beta blocker	51 (17)	59 (15)	75 (14)	80 (15)	117 (16)	93 (14)	116 (18)	107 (19)
Calcium channel blocker	14 (5)	22 (6)	21 (4)	29 (5)	32 (4)	40 (6)	44 (7)	38 (7)
Combination of antihypertensives	19 (6)	28 (7)	37 (7)	59 (10)	86 (11)	79 (11)	90 (13)	76 (12)

For peer review only

Supplementary material 2

Sensitivity analysis using mean of last two blood pressure measurements (N =2 947)

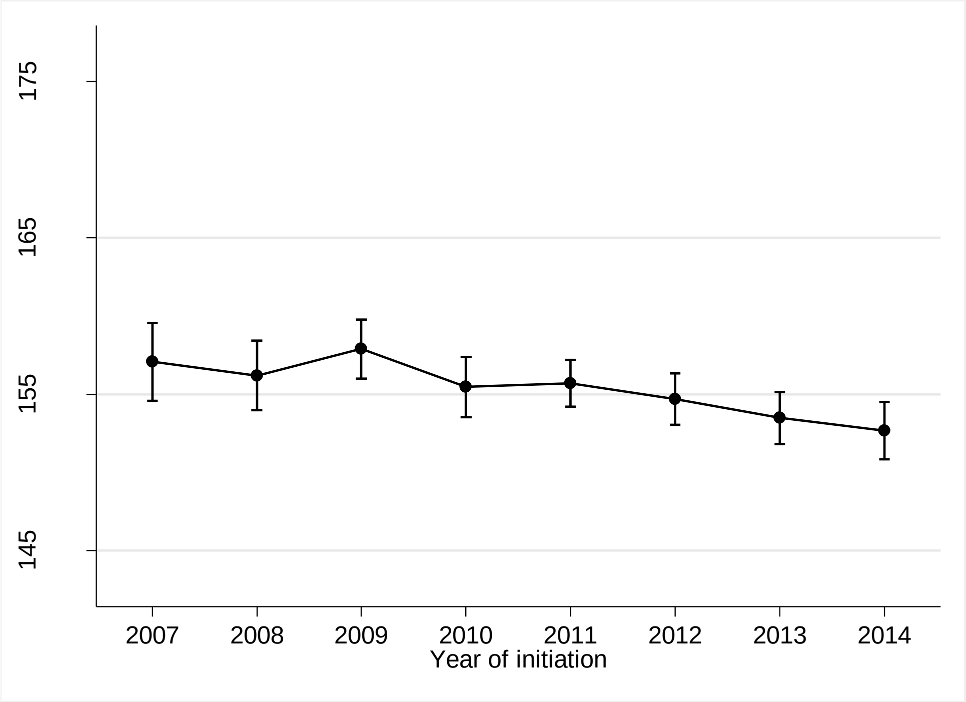

(A)

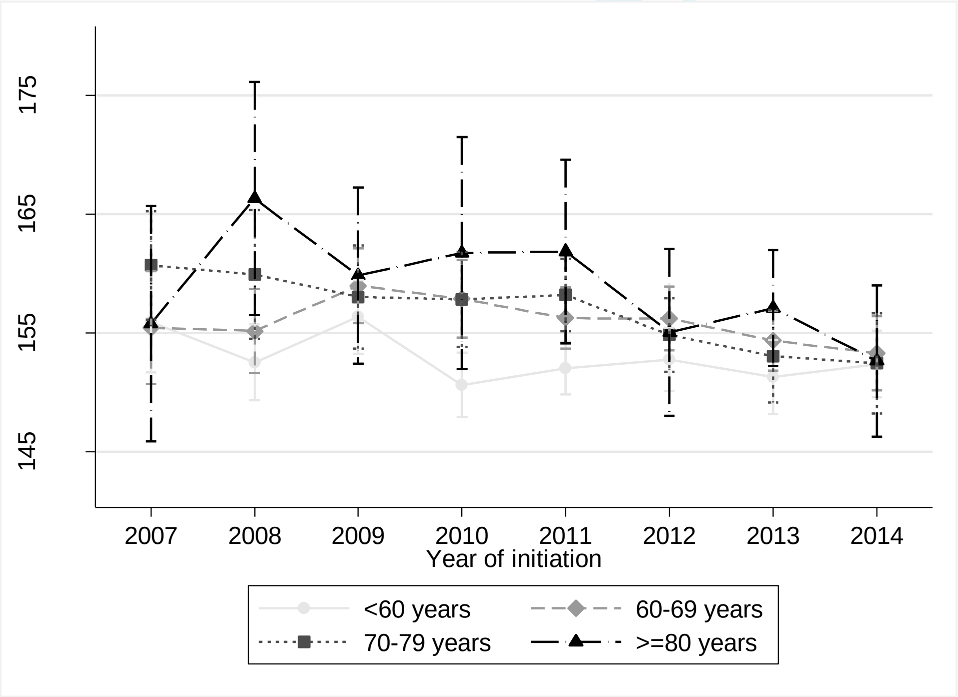

(B)

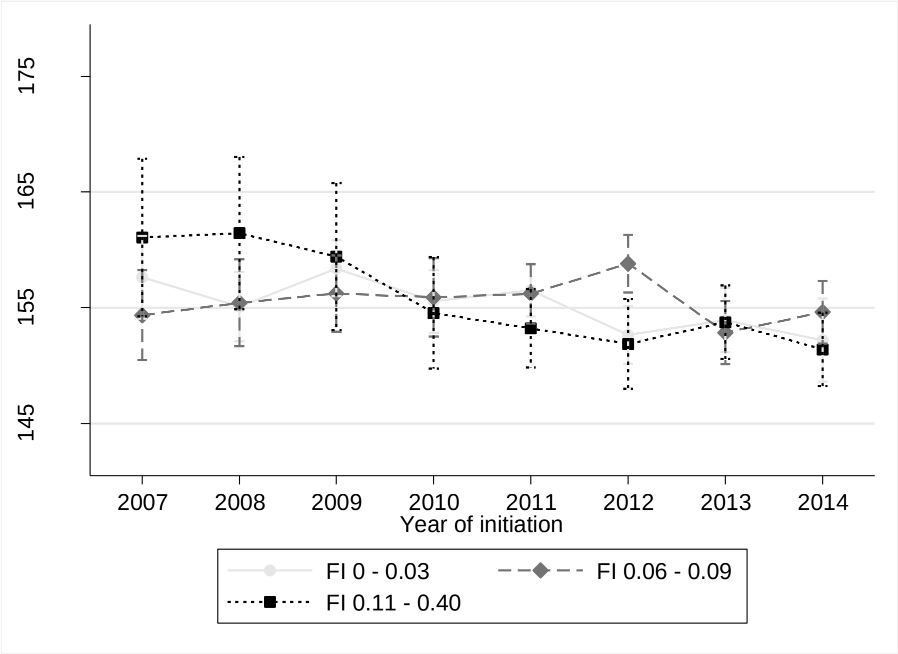

(C)

Supplementary Figure 2: Mean of last two systolic blood pressure (BP) levels with 95% CIs before/at antihypertensive treatment initiation (A) through the years; (B) through the years in different age groups and (C) through the years in different frailty groups.

Supplementary Table 2: Influence of calendar year and age or frailty on blood pressure thresholds (multilevel analysis) using the mean of last two systolic blood pressure measurements

	β	95% CI	P	
**AGE***				
Calendar year	-0.894	-2.307, 0.519	0.215	<0.001‡
Calendar year^2	-0.001	-0.146, 0.148	0.993	
Age <60 years	-8.367	-10.910, -5.824	<0.001	
Age 60 – 69 years	-4.271	-6.714, -1.828	0.001	
Age 70 – 79 years	-2.921	-5.349, -0.492	0.018	
Age ≥80 years		reference group		
Interaction year*age		none are significant		
**FRAILTY**†				
Calendar year	-0.867	-2.290, 0.556	0.232	<0.001‡
Calendar year^2	-0.013	-0.161, 0.136	0.866	
Frailty 0 – 0.03	-3.501	-5.390, -1.611	0.000	
Frailty 0.06 – 0.09	-1.455	-3.231, 0.322	0.109	
Frailty 0.11 – 0.40		reference group		
Interaction year*frailty		none are significant		

The intraclass correlation coefficient (ICC) calculated from the empty model was 0.050.

* the age model was adjusted for sex, duration of diabetes, number of chronic medication at initiation, number and/or type of glucose lowering therapy, lipid lowering therapy, presence of albuminuria, presence of dyslipidaemia, haemoglobin A1C, history of cardiovascular events, estimated glomerular filtration rate and BMI

† the frailty model was adjusted for sex, duration of diabetes, number and/or type of glucose lowering therapy, lipid lowering therapy and haemoglobin A1C

‡ joint significance of calendar year and calendar year^2 using Wald test

Supplementary material 3

Sensitivity analysis using electronic frailty index as a continuous variable

There were no statistically significant differences in blood pressure thresholds between patients with different frailty (Supplementary table 3).

Supplementary Table 3: Influence of calendar year and frailty on blood pressure thresholds (multilevel analysis), using frailty index as a continuous variable

	β	95% CI	P	
**FRAILTY**†				
Calendar year	0.211	-1.135, 1.557	0.759	<0.001‡
Calendar year^2	-0.147	-0.288, -0.007	0.039	
Frailty	-9.324	-20.747, 2.098	0.110	
Interaction year*frailty		none are significant		

† the frailty model was adjusted for sex, duration of diabetes, number and/or type of glucose lowering therapy, lipid lowering therapy and haemoglobin A1C

‡ joint significance of calendar year and calendar year^2 using Wald test

peer review only

Supplementary material 4

Supplementary Table 4: Characteristics of included patients: comparison of complete cases and cases with missing values

	**complete cases**	**cases with missings**
Patients; N (%)	3 545 (74)	1 274 (26)
Females; N (%)	1 602 (45)	675 (52)
Age in years; mean ± SD	64 ± 12	65 ± 13
Frailty index; median (Q1 - Q3)	0.08 (0.06 – 0.11)	0.08 (0.03 – 0.11)
SBP at initiation in mmHg; mean ± SD	154 ± 21	158 ± 23
DBP at initiation in mmHg; mean ± SD	85 ± 12	87 ± 13
Diabetes duration in years; mean ± SD	5.4 ± 5.3	5.4 ± 5.5
N of chronic medication at initiation; mean ± SD	3.6 ± 2.4	3.5 ± 2.6
Treated with a lipid lowering drug; N (%)	2 134 (60)	615 (48)
Initiated with one antihypertensive; N (%)	3 215 (91)	1 130 (89)

For peer review only

Supplementary material 5

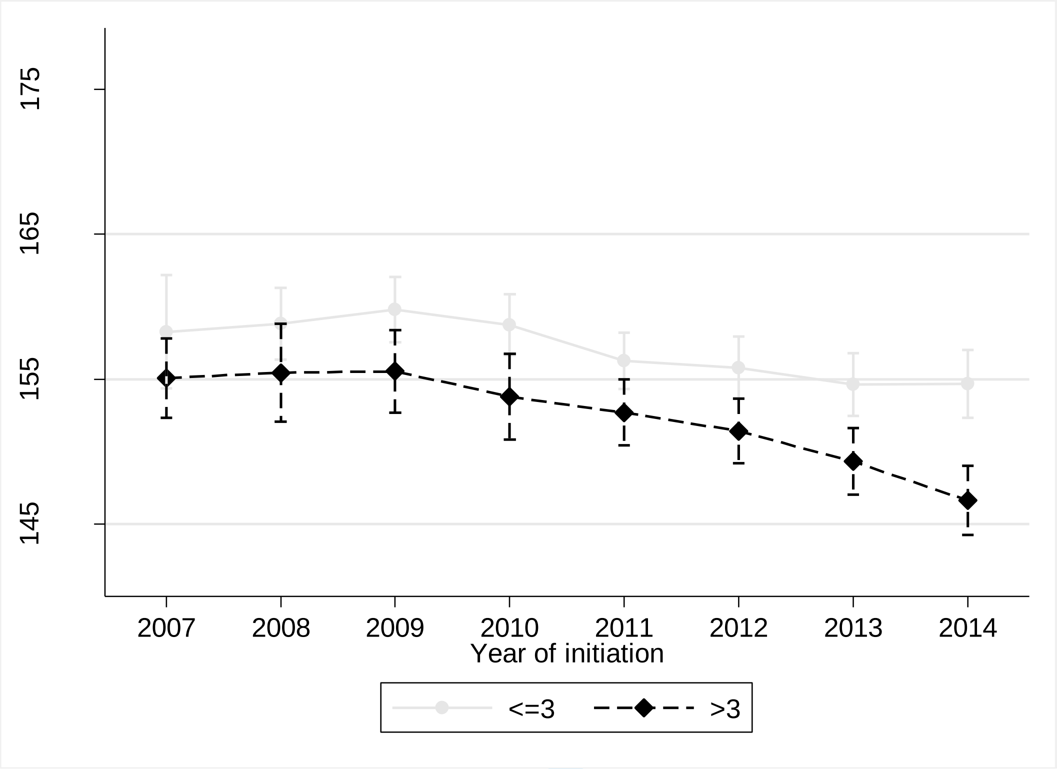

Supplementary Figure 3: Mean last systolic blood pressure value with 95% CIs before/at antihypertensive treatment initiation through the years in patients with different number of chronic medication at initiation.

Supplementary table 5: Multilevel analysis of number of chronic medication at initiation

	β	95% CI (min, max)	P	
**Calendar year**	-0.206	-1.543, 1.130	0.762	<0.001‡
**Calendar year^2**	-0.101	-0.240, 0.037	0.152	
**3 or less chronic medication**		reference group		
**More than 3 chronic medication**	-4.107	-5.428, -2.785	<0.001	
**Interaction year*N of medication**		none are significant		

The model was adjusted for sex, duration of diabetes, number and/or type of glucose lowering therapy, lipid lowering therapy, presence of albuminuria, presence of dyslipidaemia, haemoglobin A1C, estimated glomerular filtration rate and BMI.
‡ joint significance of calendar year and calendar year^2 using Wald test

For peer review only

STROBE Statement—checklist of items that should be included in reports of observational studies

	Item No	Recommendation	Page
**Title and abstract**	1	(*a*) Indicate the study's design with a commonly used term in the title or the abstract	1, 2
		(*b*) Provide in the abstract an informative and balanced summary of what was done and what was found	2
**Introduction**			
Background/rationale	2	Explain the scientific background and rationale for the investigation being reported	4
Objectives	3	State specific objectives, including any prespecified hypotheses	5
**Methods**			
Study design	4	Present key elements of study design early in the paper	5
Setting	5	Describe the setting, locations, and relevant dates, including periods of recruitment, exposure, follow-up, and data collection	5, 6
Participants	6	(*a*) *Cohort study*—Give the eligibility criteria, and the sources and methods of selection of participants. Describe methods of follow-up<br>*Cross-sectional study*—Give the eligibility criteria, and the sources and methods of selection of participants	5, 6
		(*b*) *Cohort study*—For matched studies, give matching criteria and number of exposed and unexposed	n.a.
Variables	7	Clearly define all outcomes, exposures, predictors, potential confounders, and effect modifiers. Give diagnostic criteria, if applicable	6, 7
Data sources/ measurement	8*	For each variable of interest, give sources of data and details of methods of assessment (measurement). Describe comparability of assessment methods if there is more than one group	6, 7
Bias	9	Describe any efforts to address potential sources of bias	7, 8
Study size	10	Explain how the study size was arrived at	5
Quantitative variables	11	Explain how quantitative variables were handled in the analyses. If applicable, describe which groupings were chosen and why	6, 7
Statistical methods	12	(*a*) Describe all statistical methods, including those used to control for confounding	8
		(*b*) Describe any methods used to examine subgroups and interactions	8
		(*c*) Explain how missing data were addressed	7
		(*d*) *Cohort study*—If applicable, explain how loss to follow-up was addressed<br>*Cross-sectional study*—If applicable, describe analytical methods taking account of sampling strategy	n.a.
		(*e*) Describe any sensitivity analyses	8

Continued on next page

For peer review only - http://bmjopen.bmj.com/site/about/guidelines.xhtml

**Results**			
Participants	13*	(a) Report numbers of individuals at each stage of study—eg numbers potentially eligible, examined for eligibility, confirmed eligible, included in the study, completing follow-up, and analysed	Supplementary Figure 1
		(b) Give reasons for non-participation at each stage	
		(c) Consider use of a flow diagram	
Descriptive data	14*	(a) Give characteristics of study participants (eg demographic, clinical, social) and information on exposures and potential confounders	9, 10 Supplemenary Table 1
		(b) Indicate number of participants with missing data for each variable of interest	10
		(c) *Cohort study*—Summarise follow-up time (eg, average and total amount)	n.a.
Outcome data	15*	*Cohort study*—Report numbers of outcome events or summary measures over time	10, 11
		Cross-sectional study—Report numbers of outcome events or summary measures	10, 11
Main results	16	(*a*) Give unadjusted estimates and, if applicable, confounder-adjusted estimates and their precision (eg, 95% confidence interval). Make clear which confounders were adjusted for and why they were included	10, 11
		(*b*) Report category boundaries when continuous variables were categorized	9, 10
		(*c*) If relevant, consider translating estimates of relative risk into absolute risk for a meaningful time period	n.a.
Other analyses	17	Report other analyses done—eg analyses of subgroups and interactions, and sensitivity analyses	10, 11, Supplementary material
**Discussion**			
Key results	18	Summarise key results with reference to study objectives	11, 12
Limitations	19	Discuss limitations of the study, taking into account sources of potential bias or imprecision. Discuss both direction and magnitude of any potential bias	12
Interpretation	20	Give a cautious overall interpretation of results considering objectives, limitations, multiplicity of analyses, results from similar studies, and other relevant evidence	12 - 14
Generalisability	21	Discuss the generalisability (external validity) of the study results	14, 15
**Other information**			
Funding	22	Give the source of funding and the role of the funders for the present study and, if applicable, for the original study on which the present article is based	16

For peer review only
